# The information geometry of two-field functional integrals

**DOI:** 10.1007/s41884-022-00071-z

**Published:** 2022-10-19

**Authors:** Eric Smith

**Affiliations:** 1 Earth-Life Science Institute, Tokyo Institute of Technology, 2-12-1-IE-1 Ookayama, Meguro-ku, Tokyo, 152-8550 Japan; 2 The Center for the Origin of Life, School of Chemistry and Biochemistry, Georgia Institute of Technology, 315 Ferst Drive NW, Atlanta, GA 30332 USA; 3 Santa Fe Institute, 1399 Hyde Park Road, Santa Fe, NM 87501 USA; 4 Ronin Institute, 127 Haddon Place, Montclair, NJ 07043 USA

**Keywords:** Information geometry, Doi-Peliti theory, Liouville’s theorem, Fisher information, importance sampling, duality

## Abstract

Two-field functional integrals (2FFI) are an important class of solution methods for generating functions of dissipative processes, including discrete-state stochastic processes, dissipative dynamical systems, and decohering quantum densities. The stationary trajectories of these integrals describe a conserved current by Liouville’s theorem, despite the absence of a conserved kinematic phase space current in the underlying stochastic process. We develop the information geometry of generating functions for discrete-state classical stochastic processes in the Doi-Peliti 2FFI form, and exhibit two quantities conserved along stationary trajectories. One is a Wigner function, familiar as a semiclassical density from quantum-mechanical time-dependent density-matrix methods. The second is an overlap function, between directions of variation in an underlying distribution and those in the directions of relative large-deviation probability that can be used to interrogate the distribution, and expressed as an inner product of vector fields in the Fisher information metric. To give an interpretation to the time invertibility implied by current conservation, we use generating functions to represent importance sampling protocols, and show that the conserved Fisher information is the differential of a sample volume under deformations of the nominal distribution and the likelihood ratio. We derive a pair of dual affine connections particular to Doi-Peliti theory for the way they separate the roles of the nominal distribution and likelihood ratio, distinguishing them from the standard dually-flat connection of Nagaoka and Amari defined on the importance distribution, and show that dual flatness in the affine coordinates of the coherent-state basis captures the special role played by coherent states in Doi-Peliti theory.

## Introduction: understanding the Liouville theorems that emerge in the probability analysis of dissipative systems

### Hamiltonian state spaces, conserved phase-space densities, and two-field functional integral representations

The defining feature of dissipative systems, whether classical or quantum, is that trajectories initially distinct can merge and that distributions or densities that differ at their initial conditions become more similar over time as they are increasingly governed by local generating parameters at the expense of memory. Because such systems are intrinsically irreversible, they obey no kinematic Liouville theorem (see [35]) describing phase space densities that are conserved along flow lines. For Markov jump processes on discrete state spaces, a kinematic phase space is not defined, while for stochastically perturbed dynamical systems, its volume is not preserved under time evolution. Yet in the study of the time evolution of probability distributions for stochastic processes, a conserved volume element does arise, through a Hamilton–Jacobi theory that evolves the gradients of log-probabilities together with their values [13, 69].

In the classical dynamics of Hamiltonian systems, conserved densities in phase space are associated with reversibility and made possible by the presence of canonically conjugate pairs of momentum and position coordinates specifying states. Convergence of trajectories in the projection onto either component in such a pair is disambiguated by separation in the conjugate variable, such that time evolution defines a symplectomorphism on the state space, and the data specifying a state or distribution at one time are the image under a coordinate transformation of the data at any time.

In the Hamilton–Jacobi theory that describes distributions evolving under stochastic processes, the role of the log-probability gradient as a conjugate momentum suggests that it is the resolution in continuous-valued probability distributions that can preserve the data required for backward- as well as forward-time evolution from any instant. Two questions that remain to be clarified, then, are: (1) what is the general dynamical role of these gradients as conjugate variables in a state space, in relation to the “coordinate” variables that appear in the Hamilton–Jacobi theory; and (2) what is the conceptual interpretation of conserved densities in this phase space?

An important insight into the abstract role of dual fields was given in early work by Martin, Siggia, and Rose [52], who observed that a complete system of equations similar to the Dyson equations of quantum field theory could not be formulated for stochastically perturbed dynamical systems from functions only of the coordinate field that is dynamically evolved. Their solution—appealing explicitly to quantum field theory as a model—was to re-interpret the coordinate variables in a perturbed dynamical system as operator-valued fields and to introduce a parallel set of dual fields, non-commuting with the coordinates, with the interpretation of sources of perturbation. From the two fields together, they obtained a system of relations equivalent to a Dyson equation between the advanced and retarded “response functions” to perturbations, and the correlation function of fluctuations of the coordinate variables.

The coordinate field along with the response field introduced in [52] can be re-cast from operators back to ordinary classical fields if the classical fields become variables of integration in a path integral, through the equivalence of operator formulations and path-integral formulations known in quantum mechanics since Feynman and Hibbs [28]. An especially useful observation by Kamenev [42] is that the response and correlation functions of classical stochastic processes have exactly the algebra of “observable” and “response” fields defined by Keldysh [43] as a rotation of field variables from the pairs of forward- and backward-evolving state fields in Schwinger’s time-loop formulation [63] of time-dependent quantum density matrices. Through this algebraic equivalence the role of systems of response fields introduced as duals to coordinate fields is represented generally in what we may call *two-field functional integrals* (2FFI). The path-integral formulation of the Schwinger time-loop was the original example, which the operator methods of [52] show may be extended to quite general stochastically perturbed dynamical systems.

For a certain class of Markov jump processes on discrete state spaces, which we may generally describe as stochastic population processes, a direct construction of the 2FFI representation for generating functions, from the transition matrix of the master equation, was worked out by Doi [21, 22] and Peliti [57, 58]. (This case was the path integral studied in [42].) The direct construction [44, 68, 71] makes clear the correspondence between the response field and the jump operator in the master equation, giving a mechanistic meaning to the operator-field interpretation of [52]. The Doi-Peliti integral representation also shows that it is the Liouville operator—the representation of the generator of time translation acting in the space of generating functions [74]—that is the Hamiltonian of the Hamilton–Jacobi theory [13, 69].

In the large-deviation limit, which will be the subject of this paper, the Liouville operator can be replaced with a function on deterministic phase-space trajectories, which becomes the generating function for the Lagrange–Hamilton duality between the tangent and cotangent bundles on the coordinate manifold of first moments of the dynamical distribution. Doi-Peliti theory is particularly expressive of the origin and nature of two-field duality because Peliti’s construction [57, 58] of the functional integral employs a representation of unity in the space of generating functions in terms of outer products of basis functions parametrized by the observable field, and projection operators parametrized by the response field introduced in [52]. The conservation of “data” entailed by a Liouville theorem is nothing other than the requirement of completeness in a representation of unity in a Hilbert space of generating functions.

### The information geometry induced by symplectomorphisms acting on probability distributions evolved under stochastic processes

The 2FFI representation of Hamilton–Jacobi theory will give a direct mathematical interpretation of the conserved phase space density, as the *Wigner function* of the two-field integral (see Sect. 3). This function is known in quantum mechanics as a semiclassical approximation to quantum density matrices in time-dependent thermal ensembles.

We may better understand its statistical interpretation and its relation to time reversal, however, by going beyond the simple Hamiltonian volume conservation already inherent in 2FFI theories, to derive dual parallel transport relations for vector fields induced by symplectomorphisms on the cotangent bundle, as these relate to the Riemannian geometry induced by cumulant generating functions and their Legendre duals, the large-deviation functions [73]. The construction of those aspects of the information geometry of two-field integrals is the main work of this paper.

Information geometry derives from divergence functions between probability distributions [2, 7], and provides coordinate-independent constructions for the metric distance between pairs of distributions, or the overlap between two directions of change in the form of the extended Pythagorean theorem [3, 54]. In particular, these measures are invariant under the symplectomorphisms generated by time translation, so that dynamical conservation is implied by coordinate invariance.

The probability distributions corresponding to phase-space points along the large-deviation trajectories in Doi-Peliti theory may vary either through change of the underlying probability distribution (e.g., through change in initial conditions) or through change of the argument of the generating function at which that underlying distribution is being studied. The second source of change has an interpretation in terms of biased sampling from the underlying distribution, and establishes a connection between generating functions and methods of importance sampling [56] in statistical inference.

Inference is the pertinent context within which to understand the time-reversal of Hamilton–Jacobi equations for stochastic processes, because it relates directly to the adjoint duality between the Kolmogorov forward and backward equations [33], equivalently either evolving probability distributions forward in time or operators corresponding to observables backward in time. The representation of adjoint duality in terms of time-reversed evolution has been studied extensively [37, 38, 69] in the context of fluctuation theorems [64].

Here we will study shifts in the underlying distributions evolving under some stochastic process, and shifts in the sampling bias in generating functions, as sources of vector fields which are parallel transported under the canonical transformations on phase space induced by time translation. In particular we derive affine connections under which, as differences in underlying distributions are attenuated over time due to dissipation, differences between degrees of sampling bias are amplified in the Doi-Peliti integral. The exact compensation of these two effects in Hamilton–Jacobi theory identifies conserved phase-space densities as densities of the *information relevant to inference* between protocols for biased sampling and features of underlying distributions.

We derive a particular form of this relation, in which the information delivered under varying degrees of sampling bias about the difference between nearby distributions is the inner product of two vector fields in the Fisher information metric. The conserved densities of sample-bias information under time translation are none other than the eigenvalues of the Fisher information. Dual parallel transport of the vector fields describing shifts in the underlying distribution and shifts in the sample-bias factor preserves this inner product along the one-parameter family of symplectomorphisms in the phase space generated by time translation, making the sample information about distribution change, like the phase-space density, an invariant of motion.

### Organization of the presentation

Section 2 opens with brief reviews of the three background areas needed in this work, beginning with spaces and dualities defined at a single time and then continuing to those added with time evolution. We first consider the way general probability distributions are represented by Laplace transforms as contours in phase space, on which the fundamental Riemannian metric of information geometry, and Legendre duality between conjugate phase-space coordinates, are defined. We then review the correspondence of a generating function to an exponential family of tilted distributions with the interpretation of importance distributions produced by biased sampling of the base distribution. Third we introduce time evolution under a stochastic process generator. We review the construction of the Doi-Peliti two-field integral representation of generating functions, followed by the large-deviation limit in which the Liouville operator becomes a classical Hamiltonian and the generating function for duality between coordinates in the tangent and cotangent bundles over the configuration space.

The main results of the paper are derived in Sect. 3. The Wigner function is defined and shown to be the phase space density conserved under Liouville’s theorem. The two vector fields associated with the dual coordinates in phase space are constructed, to describe variations within exponential families through change of the sample bias, and change across families through change in the base distribution. The transport law for the Fisher metric, and dual affine connections respecting the symplectic structure, are then derived.

Section 4 contains a simple worked example illustrating all aspects of the 2FFI Liouville theorem and its associated dual geometry. Section 5 concludes, relating the familiar treatment of adjoint duality in terms of time reversal [37] to the interpretation developed here as a duality between dynamics and statistical inference.

## Review: Legendre duality and information geometry, importance sampling, and Lagrange/Hamilton duality

Phase spaces with conjugate coordinates play several roles in the constructions to follow. At single times, they host the Legendre duality and Riemannian geometry of surfaces representing probability distributions through their Laplace transforms. Within the exponential family over any one distribution, conjugate fields separate the nominal distribution from other members of the family that take on the interpretation of importance distributions. When probability distributions are evolved in time, the phase spaces in which they are embedded become cotangent bundles of the dynamics, with Lagrangian–Hamiltonian dualities to the tangent bundles that depend on the generator of time translation but not on the states being evolved. In this section we establish notations for all three roles and show how they are instantiated in Doi-Peliti functional integrals.

### Phase space, foliation by exponential families, Legendre duality, and geometric relations

#### Embedding of probability distributions as surfaces in phase space, and the geometry in those surfaces

The state spaces on which the Doi-Peliti construction can be carried out to produce generating functions and functionals are non-negative integer lattices in some number of dimensions *D*, which here will be assumed finite for simplicity. Dimensions are indexed $i \in 1 , \ldots , D$, and lattice points in the state space are indexed by vectors with integer components $\textrm{n} \equiv \left( \textrm{n}_i \right)$ with each $\textrm{n}_i \ge 0$. Examples include such phenomena as stochastic population processes, in which *i* indexes the types of members in the population and each $\textrm{n}_i$ is the count of individuals of type *i*. Well-developed applications include evolutionary populations [70] and chemical reaction networks [44, 71]. The object of study will be continuous-valued probability distributions $\rho$ over states $\textrm{n}$, indexed ${\rho }_\textrm{n}$.

The Laplace transform (for a countable basis $\left\{ \textrm{n} \right\}$, also termed the *z*-*transform*) of $\rho$ is the moment-generating function

1 $$\begin{aligned} \Psi \! \left( z \right)&\equiv \sum _{\textrm{n}} \prod _i z_i^{{\textrm{n}}_i} {\rho }_{\textrm{n}} \equiv \sum _{\textrm{n}} z^{\textrm{n}} {\rho }_{\textrm{n}} \equiv e^{ \psi \left( \theta \right) } = \sum _{\textrm{n}} {\rho }_{\textrm{n}} e^{\theta \textrm{n}} \end{aligned}$$

If we write each component $z_i \equiv e^{{\theta }_i}$, $\psi \! \left( \theta \right) = \log \Psi \! \left( z \right)$ is the cumulant-generating function (CGF), which will be convex when written in coordinates $\theta$. As a shorthand we have written $z^\textrm{n}$ as the component-wise $\textrm{n}$th power of *z*. If $\textrm{n}$ is regarded as a column vector and $\theta$ a row vector, the Cartesian inner product is abbreviated $\theta \textrm{n} \equiv {\theta }^i \textrm{n}_i$, adopting the Einstein summation convention.

The gradient of $\psi \! \left( \theta \right)$, which we denote

2 $$\begin{aligned} n \! \left( \theta \right) \equiv { \left\langle \textrm{n}\right\rangle }_{ {\tilde{\rho }}^{\left( \theta \right) } } = \frac{\partial \psi }{\partial \theta } , \end{aligned}$$

is the expectation of $\textrm{n}$ in a distribution

3 $$\begin{aligned} {\tilde{\rho }}^{\left( \theta \right) }_{\textrm{n}} \equiv {\rho }_{\textrm{n}} e^{ {\theta } \textrm{n}- \psi \left( \theta \right) } \end{aligned}$$

*tilted* with weight $z^\textrm{n}$, which we will interpret later as a sampling bias or likelihood function.

The continuous coordinate vectors *n* make up the points in a manifold *Q* that will be the coordinate manifold with respect to which geometries and dynamics are defined. Pairs of values $\left( \theta , n \right)$ define a 2*D*-dimensional *phase space* over the coordinate manifold *Q*. Later, when dynamics is introduced, that phase space will become the cotangent bundle $T^{*} \! Q$ over manifold *Q*, on which a Lagrangian–Hamiltonian duality is defined. The first relation we study in this phase space, however, does not depend on its interpretation as a cotangent bundle; it is Legendre duality between values $\theta$ and *n* induced by a convex CGF $\psi$.

For convex $\psi \! \left( \theta \right)$, the function $n \! \left( \theta \right)$ is invertible, and the inverse function $\theta \! \left( n \right)$ is a *D*-dimensional surface, single-valued at each *n*, in the phase space. Each such surface has an intrinsic Riemannian geometry induced by the Hessian of $\psi$,

4 $$\begin{aligned} \frac{ {\partial }^2 \psi \! \left( \theta \right) }{ \partial {\theta }^i \partial {\theta }^j }&\equiv g_{ij} \! \left( \theta \right) , \end{aligned}$$

known as the Fisher tensor, introduced in the interpretation as a Riemannian metric by Rao [61] (reprinted as [62]). Using the differential geometry notation in which ${\left\{ \partial / \partial {\theta }^i \right\} }_{i = 1}^D$ is the set of basis elements in the tangent space to the exponential family, the Fisher metric is an inner product, which we denote

5 $$\begin{aligned} g_{ij}&\equiv \left\langle \frac{\partial }{\partial {\theta }^i} , \frac{\partial }{\partial {\theta }^j} \right\rangle \end{aligned}$$

The manifold *Q* with metric (4) admits a variety of dual transport relations induced by pairs of connections compatible with that metric, as developed by Amari and Nagaoka [3, 54]. Each hypersurface $\theta \! \left( n \right)$ is generated by a single underlying distribution $\rho$ (which we will call the “base distribution”). The whole CGF $\psi \! \left( \theta \right)$ is thus identified with an exponential family of distributions tilted from $\rho$ at each value of $z = e^{\theta }$. The dual connections of information geometry provide a way to understand the information divergences of distributions in such a family with others either inside or outside the family, in familiar geometric terms such as least-distance projections onto the family.

#### Foliations in phase space and the inner product between variations within and between exponential families

**Fig. 1:**
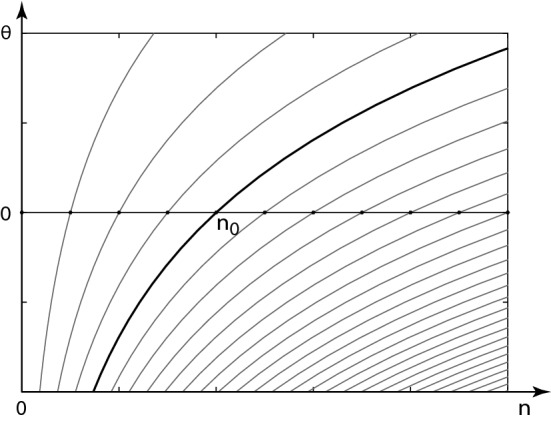
Foliation of a 1-species phase space $\left( \theta , n \right)$ by the contours $\theta \! \left( n \right) = \log \left( n / n_0 \right)$ in the exponential families over each of a sequence of Poisson base distributions with mean values $n_0$ (at $\theta = 0$). The exponential family and its associated Legendre-dual function $\theta \! \left( n \right)$ for one value of $n_0$ is drawn bold and labeled. Overcomplete bases of such Poisson distributions with continuous Poisson parameter are used in the construction of a representation of unity in the Peliti [57, 58] functional integral construction. Covariant vector fields corresponding to $\partial / \partial \theta$ (within leaves) characterize change in tilted distributions driven through sampling bias, while contravariant vector fields corresponding to $\partial / \partial n_0$ (across leaves) characterize change in the base distribution. The inner product $\delta \theta \, {\delta }_{n_0} n$ will be preserved by parallel transport of these vectors under maps of the phase space generated by time translation

We will be interested here in families of base distributions, denoted $\mathrm{\rho }_\textrm{n}^{\left( n_0 \right) }$, for which the exponential families defining the CGFs, over all values of the index $n_0$, provide a *foliation* of the phase space, as shown in Fig. 1. The CGFs and tilted distributions that make up each leaf are denoted

6 $$\begin{aligned} e^{ \psi \left( \theta , n_0 \right) }&\equiv \sum _{\textrm{n}} {\rho }^{\left( n_0 \right) }_{\textrm{n}} e^{{\theta }_{\textrm{n}}}&{\tilde{\rho }}^{\left( \theta , n_0 \right) }_{\textrm{n}}&\equiv {\rho }^{\left( n_0 \right) }_{\textrm{n}} e^{ {\theta }_{\textrm{n}} - \psi \left( \theta , n_0 \right) } \end{aligned}$$

The index $n_0$ is the value of $n \! \left( \theta \right)$ at $\theta = 0$ in the distribution ${\rho }^{\left( n_0 \right) }$.

Equation (2) implies that the Fisher metric (4) is a coordinate transformation from contravariant to covariant coordinates:

7 $$\begin{aligned} g_{ij} \! \left( \theta \right) = \frac{ \partial n_i \! \left( \theta \right) }{ \partial {\theta }^j } \end{aligned}$$

If $\psi \! \left( \theta \right)$ is convex, the transformation (7) is invertible. The inverse of the coordinate transform, and with it the Fisher metric, is obtained from the Legendre transform of $\psi \! \left( \theta \right)$,

8 $$\begin{aligned} {\psi }^{*} \! \left( n \right) \equiv { \left[ {\theta } n - \psi \! \left( \theta \right) \right] }_{\theta \left( n \right) } \end{aligned}$$

where $\theta \! \left( n \right)$ is the maximizer of the argument in Eq. (8) over $\theta$ values. ${\psi }^{*} \! \left( n \right)$ is the *Large-Deviation function* (LDF). By construction its gradient gives the inverse function

9 $$\begin{aligned} \frac{\partial {\psi }^{*} \! \left( n \right) }{\partial n_i} = {\theta }^i \! \left( n \right) . \end{aligned}$$

From the definition (1) of the generating function and the extremization condition (8), $\psi \! \left( \theta \right)$ is seen to be, in leading exponential approximation, a kind of convex approximation to $- \log {\rho }_\textrm{n}$ for $\textrm{n} \approx n$, of which $\theta$ is the gradient. These interpretations help to give meaning to the evolution of $\psi \! \left( \theta \right)$ under a Hamilton–Jacobi equation derived below in Sect. 2.3.4.

From Eq. (9), the inverse coordinate transform

10 $$\begin{aligned} \frac{\partial {\theta }^i}{\partial n_j} = \frac{ {\partial }^2 {\psi }^{*} \! \left( n \right) }{ \partial n_i \partial n_j } \equiv g^{ij} \! \left( n \right) \end{aligned}$$

is the inverse of $g \! \left( \theta \right)$ from Eq. (7). The distance between two distributions in the exponential family is then written in either contravariant coordinate differentials $\delta \theta$ or covariant coordinate differentials $\delta n$ as

11 $$\begin{aligned} \delta s^2 = \delta {\theta }^i \delta {\theta }^j g_{ij} \! \left( \theta \right) = \delta n_i \delta n_j g^{ij} \! \left( n \right) . \end{aligned}$$

The Fisher metric can be obtained as the projection of the Euclidean metric in ${\mathbb R}^D$ under a spherical embedding of the distribution ${\rho }_{\textrm{n}}$, briefly reviewed in Appendix A.1, providing a third set of coordinates for the tilted distribution ${\tilde{\rho }}^{\left( \theta \right) }$. We note this embedding because for the exponential families that will play an important role in stationary-point methods later, the distance between distributions on the Fisher sphere in potentially infinite dimensions $\left\{ \textrm{n} \right\}$ reduce to divergences of the same form on the phase space, as shown in Appendix A.2.

The distance element (11) measures the divergence between two nearby distributions in an exponential family in the geometry derived from $\psi \! \left( \theta \right)$. For two distributions related to a common base distribution through shifts respectively in $\theta$ or in $n_0$, we seek a measure of the degree to which the two changes overlap. The extended Pythagorean theorem [3, 54] for Kullback–Leibler (KL) divergences provides such a measure, where in the small-coordinate limit it becomes an inner product of the two coordinate displacements in the Fisher metric:12Recognizing that $\partial \psi / \partial {\theta }^i = n_i \! \left( \theta , n_0 \right)$, the inner product in the final line of Eq. (12) is just the sensitivity

13 $$\begin{aligned} \left\langle \frac{\partial }{\partial {\theta }^i} , \frac{\partial }{\partial n_{0j}} \right\rangle = \frac{\partial n_i}{\partial {n_0}_j} \end{aligned}$$

of the mean in the tilted distribution to variations in the base.

#### Preservation of the inner product in connection with Liouville’s theorem

A coordinate change from the mean in the base distribution to the mean in the tilted exponential distribution produces the metric in mixed coordinates that by construction is the Kronecker $\delta$,

14 $$\begin{aligned} \left\langle \frac{\partial }{\partial {\theta }^i} , \frac{\partial }{\partial n_j} \right\rangle = {\delta }_i^j , \end{aligned}$$

and the coordinate inner product

15 $$\begin{aligned} \delta {\theta }^i \delta n_{0j} \left\langle \frac{\partial }{\partial {\theta }^i} , \frac{\partial }{\partial n_{0j}} \right\rangle&\equiv \delta {\theta }^i \delta n_i . \end{aligned}$$

We may ask, for what one-parameter families of coordinate systems $\left( \theta \! \left( t \right) , n \! \left( t \right) \right)$ is the coordinate inner product (14) conserved across the family? A one-parameter family of coordinates generates a one-parameter family of maps of vector fields $\delta \theta$ and $\delta n$ by the action

16 $$\begin{aligned} { \left( \frac{d}{dt} \delta \theta \right) }^i&= \delta {\theta }^j \frac{\partial }{\partial {\theta }^j} {\dot{\theta }}^i&{ \left( \frac{d}{dt} \delta n \right) }_i&= \delta n_j \frac{\partial }{\partial n_j} {\dot{n}}_i . \end{aligned}$$

The condition

17 $$\begin{aligned} \frac{d}{dt} \left( \delta {\theta }^i \, \delta n_i \right) = 0 \end{aligned}$$

will be met whenever

18 $$\begin{aligned} \frac{ \partial {\dot{\theta }}^i }{ \partial {\theta }^j } = - \frac{ \partial {\dot{n}}_j }{ \partial n_i } , \end{aligned}$$

where $\cdot$ denotes *d*/*dt*. Eq. (18) is satisfied if there is a symplectic form $\mathcal {L} \! \left( \theta , n \right)$ in terms of which the velocity vectors along trajectories can be written

19 $$\begin{aligned} {\dot{\theta }}_i&= \frac{\partial }{\partial n_i} \mathcal {L} \! \left( \theta , n \right)&{\dot{n}}_i&= - \frac{\partial }{\partial {\theta }_i} \mathcal {L} \! \left( \theta , n \right) . \end{aligned}$$

Alternatively, if Eq. (18) holds everywhere, the form $\mathcal {L} \! \left( \theta , n \right)$ can be constructed by integration.

Eq. (18) relates the dual contravariant and covariant coordinates under the Fisher metric as canonically conjugate variables in a Hamiltonian dynamical system. We return in Sect. 2.3 to derive symplectic forms $\mathcal {L} \! \left( \theta , n \right)$ from the generators of stochastic processes.

### Tilted distributions as importance distributions, and particular application to large deviations

In order to furnish an interpretation of the 2FFI symplectic transport structure, we appeal to statistical inference to assign informational meanings to the conjugate fields *z* that exponentially tilt the base distributions in generating functions. In importance sampling, *z* takes on the interpretation of a sampling bias known as a likelihood ratio, which transforms the measure for samples.

In the terminology of importance sampling [56], the base distribution ${\rho }^{\left( n_0 \right) }$ corresponds to the *nominal* distribution, and the tilted distribution ${\tilde{\rho }}_{\textrm{n}}^{\left( \theta , n_0 \right) }$ of Eq. (6) plays the role of an *importance distribution*. The normalized exponential tilt $e^{{\theta } \textrm{n}- \psi \left( \theta , n_0 \right) }$ is the corresponding *likelihood ratio*, also called the *Radon–Nikodym derivative* of the measure between the base and the importance distributions.

Importance distributions ${\tilde{\rho }}^{\left( \theta , n_0 \right) }$ can be chosen to concentrate the density of samples away from the mode of ${\rho }^{\left( n_0 \right) }$ to values of $\textrm{n}$ that are more informative about observables of interest. Tilts are typically chosen to minimize some cost function, such as the variance of samples. The large-deviation function can be derived as a leading exponential approximation to the tail weight of the base distribution, in a protocol tuned to minimize sample variance, as shown in the following construction from [66].

To illustrate with an example in one dimension, an estimate of the probability that a particle count $\textrm{n}$ exceeds some bound $\bar{n}$ can be obtained by sampling values of the random variable

20 $$\begin{aligned} h^{\left( \bar{n} \right) }_{\textrm{n}} \equiv I \! \left\{ \textrm{n}> \bar{n} \right\} , \end{aligned}$$

the indicator function for $\textrm{n}> \bar{n}$. In the base distribution ${\rho }^{\left( n_0 \right) }$, the probability for $\textrm{n}> \bar{n}$ is

21 $$\begin{aligned} P \! \left( \textrm{n}> \bar{n} \mid n_0 \right) = {\left\langle h^{\left( \bar{n} \right) } \right\rangle }_{\left( n_0 \right) } . \end{aligned}$$

An unbiased estimator for $P \! \left( \textrm{n}> \bar{n} \mid n_0 \right)$ can be obtained by using the tilted distribution ${\tilde{\rho }}_{\textrm{n}}^{\left( \theta , n_0 \right) }$ of Eq. (6) and instead of accumulating the values $\left\{ 0 , 1 \right\}$ of the indicator $h^{\left( \bar{n} \right) }$, accumulating values of the tilted observable

22 $$\begin{aligned} {\tilde{h}}^{\left( \theta , \bar{n} \right) }_{\textrm{n}}&\equiv e^{ \psi \left( \theta , n_0 \right) - \theta \textrm{n}} h_{\textrm{n}} . \end{aligned}$$

The tilted estimator is unbiased because

23 $$\begin{aligned} { \left\langle {\tilde{h}}^{\left( \theta , \bar{n} \right) } \right\rangle }_{ \left( \theta , n_0 \right) } = {\left\langle h^{\left( \bar{n} \right) } \right\rangle }_{\left( n_0 \right) } . \end{aligned}$$

A few lines of algebra, provided in Appendix B, show that an exponential bound for the estimator at any choices of $\theta$ and $\bar{n}$ is given by

24 $$\begin{aligned} { \left\langle {\tilde{h}}^{\left( \theta , \bar{n} \right) } \right\rangle }_{ \left( \theta , n_0 \right) }&\le e^{ \psi \left( \theta , n_0 \right) - \theta \bar{n} } . \end{aligned}$$

The variance of the same sample estimator has a corresponding bound (see Eq. (146))

25 $$\begin{aligned} { \left\langle { \left( {\tilde{h}}^{\left( \theta , \bar{n} \right) } \right) }^2 \right\rangle }_{\left( \theta , n_0 \right) } - { \left\langle {\tilde{h}}^{\left( \theta , \bar{n} \right) } \right\rangle }_{ \left( \theta , n_0 \right) }^2 \le e^{ \psi \left( \theta , n_0 \right) - \theta \bar{n} } {\left\langle h^{\left( \bar{n} \right) } \right\rangle }_{\left( n_0 \right) } - {\left\langle h^{\left( \bar{n} \right) } \right\rangle }_{\left( n_0 \right) }^2 . \end{aligned}$$

The parameter $\theta$ that minimizes the bound on sample variance (25) also gives the tightest bound (24) on the tail weight. It is the minimizing argument $\theta \! \left( \bar{n} \right)$ of Eq. (8), so the bound is given in terms of the LDF as.

26 $$\begin{aligned} {\left\langle h^{\left( \bar{n} \right) } \right\rangle }_{\left( n_0 \right) }&\le e^{- {\psi }^{*} \left( \bar{n} \right) } . \end{aligned}$$

Without further assumptions about ${\rho }^{\left( n_0 \right) }$ it is not possible to say more about the ratio ${\left\langle h^{\left( \bar{n} \right) } \right\rangle }_{\left( n_0 \right) } / e^{- {\psi }^{*} \left( \bar{n} \right) }$. The relevant additional property, which is also associated with the use and tightness of saddle-point approximations [36] in Doi-Peliti theory, is the onset of large-deviations scaling; that is, if $n_0$ and $\bar{n}$ are increased together in proportion to some scale factor *N* as $\bar{n} = N \bar{\nu }$, $n_0 = N {\nu }_0$, the following two limits should exist:

27 $$\begin{aligned} \lim _{N \rightarrow \infty } \frac{1}{N} {\psi }^{*} \! \left( N \bar{\nu } \right)&= {\bar{\psi }}^{*} \! \left( \bar{\nu } \right)&\lim _{N \rightarrow \infty } \frac{1}{N} \psi \! \left( \theta \! \left( N \bar{\nu } \right) ; N {\nu }_0 \right)&= \bar{\psi } \! \left( \bar{\theta } \right) . \end{aligned}$$

Then the variance-minimizing tilt $\theta$ likewise has a limit, the variance ${\partial }^2 \psi / \partial {\theta }^i \partial {\theta }^j$ in Eq. (4) scales as *N*, and the relative variance scales as 1/*N*. Appendix B shows that in this limit the log ratio $\log \left[ {\left\langle h^{\left( \bar{n} \right) } \right\rangle }_{\left( n_0 \right) } / e^{- {\psi }^{*} \left( \bar{n} \right) } \right] \le \mathcal {O} \! \left( N^{1/2} \right)$, compared to ${\psi }^{*} \! \left( \bar{n} \right) \sim N$.

### The Doi-Peliti integral construction of generating functions for time-dependent probability distributions

Canonical transformations on phase space [35] are introduced when distributions $\rho$ are evolved under the generators of stochastic processes. A formally exact representation of the time-translation of distributions over finite intervals, acting directly in the representation by generating functions, is given by the Doi-Peliti construction [21, 22, 57, 58]. (See [9, 42, 68] for other self-contained introductions.) Phase-space coordinates in Doi-Peliti theory take on interpretations in terms of distributions and dual projection operators, equivalent in the path integral to the non-commuting operator fields of [52], through Peliti’s construction [57, 58] of a representation of the identity in the Hilbert space of generating functions.

In the path integral, tangent vectors $\dot{n}$ to configurations, and phase-space dual coordinates $\theta$, are independent dummy variables of integration. It is the large-deviation limit, realized in Doi-Peliti theory by saddle-point evaluation of the functional integral, that couples coordinate pairs $\left( \dot{n} , n \right)$ and $\left( \theta , n \right)$ as Lagrangian and Hamiltonian dual coordinate systems, with the Liouville operator-representation of the stochastic process generator as the Hamiltonian and the generating function of the coordinate transformation. The tangent vectors to stationary trajectories generate a family of symplectomorphisms in phase space, and the LDF evolves as a solution to the corresponding Hamilton–Jacobi equation [13, 69].

A tutorial on the defining steps in Doi-Peliti theory is given in this subsection. Supporting algebra, where needed to provide a complete self-contained definition of the method, is provided in Appendix C. Doi-Peliti theory was invented to solve Markov jump processes on integer lattices of the kind that describe population processes, and while providing a definite notation and formally-exact integral solution, neither the indexing nor the particular exponential generating functions used here are meant to be a general notation describing all 2FFIs. Appendix D, working in the simplifying limit of Gaussian fluctuations, provides derivations starting from Doi-Peliti theory of equivalent representations in terms of the Langevin stochastic differential equation and the Fokker–Planck equation [74], which manifestly have no restriction to the specific assumptions made in the Doi-Peliti construction. The derivations leading to them may be “inverted” to equivalence classes of 2FFI path integrals in the Gaussian limit, defined by a common integral kernel form explained in [42], and equivalent to the Dyson equations for general stochastic processes derived in [52]. Saddle-point methods in Doi-Peliti theory are seen to be one instance of the eikonal approximations for large-deviation functions, developed in general form by Freidlin and Wentzell [30], and the forms derived from the action here are related to specific forms arising in that work.

#### Generator, Hilbert space, and quadrature

The starting representation for time evolution is the continuous-time master equation of the probability distribution $\rho$

28 $$\begin{aligned} \frac{d {\rho }_{\textrm{n}}}{dt}&= \sum _{{\textrm{n}}^{\prime }} \textrm{T}_{\textrm{n}{\textrm{n}}^{\prime }} {\rho }_{{\textrm{n}}^{\prime }} . \end{aligned}$$

$\textrm{T}_{\textrm{n}{\textrm{n}}^{\prime }}$ is known as the *transition matrix*, and is the representation of the generator of the stochastic process acting in the space of probability distributions. It is left-stochastic, meaning $\sum _{\textrm{n}} \textrm{T}_{\textrm{n}{\textrm{n}}^{\prime }} = 1$, $\forall {\textrm{n}}^{\prime }$, ensuring conservation of probability. The matrix elements $\textrm{T}_{\textrm{n}{\textrm{n}}^{\prime }}$ can depend on the time *t*, though in the example developed in Sect. 4 we will use a time-independent generator for simplicity.

The *z*-transform (1) induces a representation of the generator of time translation acting in the space of moment generating functions in the form of a Liouville equation

29 $$\begin{aligned} \frac{\partial }{\partial t} \Psi \! \left( z \right)&= - \mathcal {L} \! \left( z , \frac{\partial }{\partial z} \right) \Psi \! \left( z \right) , \end{aligned}$$

$\mathcal {L} \! \left( z , \partial / \partial z \right)$, called the *Liouville operator*, is conventionally defined with the minus sign of Eq. (29) because its spectrum is non-negative.

For many purposes, the properties of the generating function as an analytic function of a complex variable [29] are not needed, and the algebra of the MGF as a formal power series [77] is sufficient. An operator algebra due to Doi [21, 22] replaces the variable *z* and derivative $\partial / \partial z$ with formal raising and lowering operators

30 $$\begin{aligned} z_i&\rightarrow a^{\dagger }_i ;&\frac{\partial }{\partial z_i}&\rightarrow a^i . \end{aligned}$$

In the condensed notation (1) for vector inner products, we will regard *a* as a column vector and $a^{\dagger }$ as a row vector.

Associated with operators $a^{\dagger }_i$ and $a^i$ are bilinear *number operators* ${\hat{n}}_i \equiv a^{\dagger }_i a^i$ (no Einstein sum), of which the basis monomials $z^{\textrm{n}}$ under the mapping (30) correspond to number eigenstates. Number states are denoted $\left| \textrm{n}\right)$, and the MGF $\Psi \! \left( z \right)$ is represented as a state vector $\left| \Psi \right)$ defined in terms of number states as

31 $$\begin{aligned} \sum _{\textrm{n}} {\rho }_{\textrm{n}} \left| \textrm{n}\right) \equiv \left| \Psi \right) . \end{aligned}$$

The Liouville equation (29) becomes

32 $$\begin{aligned} \frac{d}{dt} \left| \Psi \right)&= - \mathcal {L} \! \left( a^{\dagger } , a \right) \left| \Psi \right) , \end{aligned}$$

in which the the Liouville operator $\mathcal {L} \! \left( a^{\dagger } , a \right)$ is the former function $\mathcal {L} \! \left( z , \partial / \partial z \right)$ under the substitution (30).

An inner product must be defined to make generating functions states in a Hilbert space. In the Doi theory this is done formally by introducing a dual state $\left( 0 \right|$ satisfying $\left( 0 \mid 0 \right) = 1$. Conjugate number states $\left( \textrm{m} \right|$ with inner product $\left( \textrm{m} \mid \textrm{n} \right) = {\delta }_{\textrm{m} \textrm{n}}$ are then built up using lowering operators. The correspondence of the Doi dual ground state with a projector on analytic generating functions, and the inner product known as the *Glauber norm*, corresponding to the evaluation of any MGF at argument $z = 1$, are given in Appendix C.1. The analytic form (1) of the MGF can be recovered using a variant of the Glauber norm, as

33 $$\begin{aligned} \left( 0 \right| e^{z a} \left| \Psi \right) = \Psi \! \left( z \right) . \end{aligned}$$

The objective in introducing the Doi operator formalism is to more conveniently compute the quadrature of the Liouville equation (29), formally written

34 $$\begin{aligned} {\left| \Psi \right) }_T&= \mathcal {T} e^{ - \int _0^T dt \mathcal {L} \left( a^{\dagger }, a \right) } {\left| \Psi \right) }_0 \nonumber \\&\equiv \lim _{\delta t \rightarrow 0} \mathcal {T} \prod _{k = 1}^{T / \delta t} e^{ - \delta t { \mathcal {L} \left( a^{\dagger }, a \right) }_{k \delta t} } {\left| \Psi \right) }_0 . \end{aligned}$$

${\left| \Psi \right) }_T$ is the generating function for the distribution ${\rho }_{\textrm{n}}$ evolved to time $t = T$ from the generating function ${\left| \Psi \right) }_0$ for an initial distribution given at time $t = 0$. $\mathcal {T}$ denotes time-ordering of the exponential integral, defined operationally in the second line of Eq. (34), in terms of a time-ordered product of applications of $\mathcal {L} \! \left( a^{\dagger }, a \right)$ evaluated at the sequence of times $k \delta t$.

#### The Peliti coherent-state expansion and two-field functional integral

The implications of the time-ordered operator algebra in the quadrature (34) for correlation functions may not be at all easy to derive or approximate, and the purpose of Peliti’s functional-integral construction is to supply evaluation methods, and even more importantly, approximation methods including saddle-point evaluations and a systematic approach to perturbation theory such as exists for path-integral formulations in quantum mechanics [28].

A straightforward step in the Peliti construction is to expand arbitrary generating functions in a basis of eigenstates of the lowering operator *a*. However, whereas the indices $\left\{ \textrm{n} \right\}$ are countable, the eigenvalues of *a* are continuous, so such a basis is overcomplete. The important and non-trivial step in the Peliti construction is to identify a representation of the identity operator in the space of generating functions, which requires defining projectors that are left-eigenstates of the raising operator $a^{\dagger }$, and showing that an expansion in these two continuously-indexed sets of states is equivalent to the identity operator on number states $\textrm{n}$. It is in this second step that the response field in phase space enters the functional-integral construction for stochastic processes.

The eigenstates of the lowering operator will be the moment-generating functions of products of Poisson distributions, and these distributions will play a central role in the interpretation of stationary-point approximations in the functional integral. In the Poisson distribution with mean *n*,

35 $$\begin{aligned} {\rho }^{\left( n_i \right) }_{{\textrm{n}}_i} = e^{-n_i} \frac{ n_i^{{\textrm{n}}_i} }{ {\textrm{n}}_i ! } , \end{aligned}$$

the expectations of *factorial moments* [10], defined (again, component-wise) as ${\textrm{n}}^{\underline{k}} \equiv {\textrm{n}} ! / {\left( \textrm{n}- k \right) } !$, are $n^k$ for all *k*. Products of Poisson marginals, or multinomial distributions that are sections through such products at fixed $N \equiv \sum _i n_i$,

36 $$\begin{aligned} {\rho }^{\left( n \right) }_{\textrm{n}} = \frac{1}{N^N} \left( \frac{ N ! }{ {\textrm{n}}_1 ! , \ldots , {\textrm{n}}_D ! } \right) \prod _{i = 1}^D n_i^{{\textrm{n}}_i} , \end{aligned}$$

both serve as saddle-point approximations to more general distributions in the Doi-Peliti integral, and also form an important class of exact solutions for some applications such as chemical reaction network models [4]. Tilts of Poisson (35) or multinomial (36) distributions remain of the same form, so that nominal and importance distributions will have a uniform relation to phase-space points at all pairs $\left( \theta , n \right)$ in Fig. 1.

The fixed form of all moments in Poisson and multinomial distributions as functions of the mean makes these *minimum-information* distributions. For these distributions the Fisher spherical embedding of divergences of distributions $\rho$ on the infinite index set $\left\{ \textrm{n} \right\}$ can be reduced to only *D* dimensions in the coordinates $\left\{ n_i / N \right\}$, as shown in Appendix A.2. Related simplifications, from forms of adjoint duality [38] that generally require infinitely many parameters, to simple coordinate transforms in the Doi-Peliti functional integral, are reviewed below in Sect. 2.3.6.

The generating function of a product distribution with a vector of Poisson parameters $\phi \equiv \left( {\phi }^i \right)$ corresponds to the state

37 $$\begin{aligned} \left| \phi \right)&\equiv e^{ \left( a^{\dagger } - 1 \right) \phi } \left| 0 \right) , \end{aligned}$$

as may be checked by an elementary expansion of the Taylor’s series for the exponential. These states are eigenstates of the lowering operators:

38 $$\begin{aligned} a^i \left| \phi \right) = {\phi }^i \left| \phi \right) . \end{aligned}$$

From the inner product of the base projection operator $\left( 0 \right|$ and number states $\left( \textrm{m} \right|$ introduced above, a similar Taylor’s series expansion of the exponential verifies that left eigenstates of the raising operator must take the form

39 $$\begin{aligned} \left( {\phi }^{\dagger } \right|&\equiv e^{ \left( 1 - {\phi }^{\dagger } \right) \phi } \left( 0 \right| e^{ {\phi }^{\dagger } a } . \end{aligned}$$

The normalization in Eq. (39) has been chosen for convenience, so that the inner product of two such coherent states will evaluate to

40 $$\begin{aligned} \Big ( {\phi }^{\dagger }_2 \, \Big | {\phi }_1 \Big )&= e^{ \left( 1 - {\phi }^{\dagger }_2 \right) \left( {\phi }_2 - {\phi }_1 \right) } , \end{aligned}$$

and in particular $\big ( {\phi }^{\dagger } \big | \phi \big ) = 1$.

Note that the inner product (40), if expanded in the power series for the two coherent states, identifies a tilted distribution

41 $$\begin{aligned} {\tilde{\rho }}_\textrm{n} = e^{- {\phi }^{\dagger }_2 {\phi }_1} \frac{ {\left( {\phi }^{\dagger }_2 {\phi }_1 \right) }^\textrm{n} }{ \textrm{n} ! } , \end{aligned}$$

so we recognize that the coherent-state parameters $\phi$ index a space of base distributions, and the dual fields ${\phi }^{\dagger }$ are the biased-sampling weights in an exponential family of generating functions in which $\log {\phi }^{\dagger }$ takes the place of the coordinate $\theta$ in Eq. (3). In the language of importance sampling from Sect. 2.2, Eq. (41) are importance distributions with mean parameters $n_i = {\phi }^{*}_{2i} {\phi }_{1i}$.

The result enabling the Peliti functional integral construction is that the following integral of outer products constitutes a representation of the identity in the space of generating functions:

42 $$\begin{aligned} \int \frac{ d^D \! {\phi }^{\dagger } d^D \! \phi }{ {\pi }^D } \Big | \phi \Bigg ) \Bigg ( {\phi }^{\dagger } \Big |&= I , \end{aligned}$$

a result demonstrated in Eq. (160) of Appendix C.2.

From the insertion of copies of the identity (42) at a sequence of times $t = k \delta t$ for some small $\delta t$ into the quadrature (34), some algebra gives the generating function (1) in the form of a functional integral with what Feynman and Hibbs [28] term a “skeletonized” functional measure (defined in Eq. (162)), as

43 $$\begin{aligned} e^{{\psi }_T \left( \theta \right) } = \int _0^T {\mathcal {D}}^D \! {\phi }^{\dagger } {\mathcal {D}}^D \! {\phi } \, e^{ \left( z - {\phi }^{\dagger }_T \right) {\phi }_T - S + {\psi }_0 \left( \log {\phi }^{\dagger }_0 \right) }. \end{aligned}$$

$e^{{\psi }_0 \left( \log {\phi }^{\dagger }_0 \right) }$ is the initial generating function ${\left| \Psi \right) }_0$ in Eq. (34). *S* in Eq. (43) has the form of a Lagrange–Hamilton *action* functional,

44 $$\begin{aligned} S&= \int _0^T dt \left\{ - \left( d_t {\phi }^{\dagger } \right) \phi + \mathcal {L} \! \left( {\phi }^{\dagger } , \phi \right) \right\} , \end{aligned}$$

in which the “kinetic term” that creates a Doi-Peliti Lagrangian comes from the inner product (40) between projectors and states in two expansions for the identity operator at closely-spaced times.

While the CGF (43) is formally a function of the coordinate $\theta = \log z$, the functional integral (44) and action (43) are at the moment expressed in terms of coherent-state fields $\left( {\phi }^{\dagger } , \phi \right)$. As noted in Eq. (41), these variables are on one hand mathematically expressive, as they distinguish the mean in a base distribution from the likelihood ratio in an importance distribution. On the other hand they make calculation of the mean in the importance distribution inconvenient because it is a bilinear form in ${\phi }^{\dagger }$ and $\phi$, and more importantly, the Hessian of the function ${\psi }_T$ is not generally positive-definite in coordinates *z*.

Therefore we introduce the first canonical transformation from the Peliti coherent-state coordinates that will be of interest in this work, which is a simple logarithmic point transformation

45 $$\begin{aligned} {\phi }_i^{\dagger }&\equiv e^{{\theta }^i}&{\phi }_i&\equiv e^{-{\theta }^i} n_i . \end{aligned}$$

The fields *n* and $\theta$ would have the interpretation of a mean molecule number and conjugate chemical potential in a chemical-reaction model, so we refer to these as number-potential coordinates, distinguishing them from coherent-state coordinates. The action (44) becomes

46 $$\begin{aligned} S&= \int _0^T dt \left\{ - \left( d_t {\theta } \right) n + \mathcal {L} \! \left( \theta , n \right) \right\} , \end{aligned}$$

in which $\mathcal {L} \! \left( \theta , n \right)$ is $\mathcal {L} \! \left( {\phi }^{\dagger } , \phi \right)$ with ${\phi }^{\dagger }$ and $\phi$ written as functions of *n* and $\theta$ by Eq. (45).

#### The large-deviation limit and Hamiltonian stationary trajectories

The large-deviation limit of the path integral (43) is a leading-exponential approximation in some value such as $\phi$ that becomes large to reflect large population size or a similar scale variable. In that limit the integral is approximated by the value of the exponential kernel at its saddle point, which is any stationary solution of the action *S* and the initial and final boundary terms. The stationary trajectories for *S* in the form (44) satisfy the pair of equations of motion

47 $$\begin{aligned} {\dot{\phi }^{\dagger }}_i&= \frac{\partial }{\partial {\phi }_i} \mathcal {L} \! \left( {\phi }^{\dagger } , \phi \right)&{\dot{\phi }}_i&= - \frac{\partial }{\partial {\phi }^{\dagger }_i} \mathcal {L} \! \left( {\phi }^{\dagger } , \phi \right) . \end{aligned}$$

The final-time boundary value for the field ${\phi }^{\dagger }$ is given by the vanishing derivative of the exponent in Eq. (43) with respect to ${\phi }_T$, resulting in ${\phi }^{\dagger }_T = z$. The initial time boundary value for $\phi$ is given, after an integration by parts, by the vanishing derivative with respect to ${\phi }^{\dagger }_0$, and depends on the form of the CGF ${\psi }_0$ and the stationary-path value of its argument ${\phi }^{\dagger }_0$. The two conditions are solved self-consistently through the Eq. (47).

Joint stationary values for $\left( {\phi }^{\dagger } , \phi \right)$ at intermediate times *t*, in a generating function with argument *z* imposed at a final time *T*, represent the bundle of rays in the statistical model that dominate the contribution to the importance distribution at later times. For this reason the stationary trajectory in the base distribution is not generally independent of the trajectory for the tilt in systems with non-linear equations of motion.

The stationary-path equations corresponding to Eq. (47) in the logarithmic number-potential coordinates (45) are

48 $$\begin{aligned} {\dot{\theta }}_i&= \frac{\partial }{\partial n_i} \mathcal {L} \! \left( \theta , n \right)&{\dot{n}}_i&= - \frac{\partial }{\partial {\theta }_i} \mathcal {L} \! \left( \theta , n \right) . \end{aligned}$$

These equations are instances of the symplectomorphisms (19). The triangle inequality of divergences (12) within and across exponential families is therefore an invariant of motion.

#### Duality with the Lagrangian, and the Hamilton–Jacobi equation

The equation of motion (48) for *n* introduces a coordinate transform from phase-space coordinates $\left( \theta , n \right)$ to coordinates $\left( \dot{n} , n \right)$ in the tangent bundle *TQ* over manifold *Q* of *n* values. $- \mathcal {L} \! \left( \theta , n \right)$ is the generating function for the transformation and the Hamiltonian on phase space.

The Lagrangian dual to $-\mathcal {L}$,

49 $$\begin{aligned} L \! \left( \dot{n} , n \right)&\equiv { \left. \left\{ {\theta } \dot{n} + \mathcal {L} \! \left( \theta , n \right) \right\} \right| }_{\theta \left( \dot{n} , n \right) } , \end{aligned}$$

is the argument of the action *S* up to a total time derivative in ${\theta } n$. By construction, *L* is the generating function of the inverse coordinate transform, satisfying

50 $$\begin{aligned} { \left. \frac{\partial }{\partial {\dot{n}}_i} L \! \left( \dot{n} , n \right) \right| }_n&= {\theta }_i&{ \left. \frac{\partial }{\partial {n}_i} L \! \left( \dot{n} , n \right) \right| }_{\dot{n}}&= { \left. \frac{\partial }{\partial n_i} \mathcal {L} \! \left( \theta , n \right) \right| }_{\theta } . \end{aligned}$$

Thus Lagrange–Hamilton duality identifies the phase space with the cotangent bundle $T^{*} \! Q$ to the coordinate manifold.

For any initial distribution $\rho$ encoded in the initial-time generating function ${\psi }_0 \! \left( {\theta }_0 \right)$ in Eq. (43), we may write the action (46) evaluated along a stationary trajectory as

51 $$\begin{aligned} S&= - { \left. {\theta } n \right| }_0^T + \int _{n_0}^{n_T} {\theta } dn + \int _0^T \mathcal {L} \! \left( \theta , n \right) dt . \end{aligned}$$

After performing the Legendre transform (8) to the LDF to cancel the surface term in Eq. (51), we find that ${\psi }^{*} \! \left( n \right)$, at any time, satisfies the pair of equations

52 $$\begin{aligned} \frac{\partial {\psi }^{*}}{\partial n}&= {\theta }&\frac{\partial {\psi }^{*}}{\partial t}&= \mathcal {L} \! \left( \frac{\partial {\psi }^{*}}{\partial n} , n \right) . \end{aligned}$$

The Hamiltonian equations (48) generate a family of maps of phase space, and under any of these maps the initial-value contour ${\theta }_0 \! \left( n \right)$ for any distribution $\rho$ is mapped to a contour ${\theta }_t \! \left( n \right)$, along which ${\psi }^{*}_t \! \left( n \right)$ is a solution to the Hamilton–Jacobi equation (52).

#### The commutative diagram of maps from Legendre and Lagrangian–Hamiltonian dualities

The Legendre and Lagrange–Hamilton dualities of Doi-Peliti theory define a system of *D*-dimensional coordinate transformations that form a commutative diagram, summarized by Loek and Zhang [47]. Equations (48, 49) define a map $\left( \dot{n} , n \right) \leftrightarrow \left( \theta , n \right)$ between the tangent and cotangent bundles, $TQ \leftrightarrow T^{*} \! Q$, describing the same trajectories in respectively Lagrangian and Hamiltonian coordinates. Integration of these equations over any finite time interval *t* defines a map from pairs of initial and final points $\left( n_t, n_0 \right)$ in $Q \times Q$ to points in *TQ* at either initial or final times, by assigning initial and final velocities $\left( {\dot{n}}_t, {\dot{n}}_0 \right)$ at those points. Combined with the maps $TQ \leftrightarrow T^{*} \! Q$ at both times, the Lagrangian map between times generates a symplectomorphism in the cotangent bundle $T^{*} \! Q \leftrightarrow T^{*} \! Q$ equal to the direct integration along stationary trajectories (48).

The Legendre duality $\theta \! \left( n \right)$ at a single time is defined separately on the exponential families over each of the base distributions ${\rho }^{\left( n_0 \right) }$ that form the leaves in the foliation of Fig. 1, but does not depend on the generator of time translation. The Lagrange–Hamilton duality depends on the generator of time translations and not on the choice of exponential families used to foliate the cotangent bundle. Because points $\left( \theta , n \right)$ on stationary trajectories in Doi-Peliti theory correspond to tilted distributions over Poisson base distributions (35), the canonical foliation of phase space in Fig. 1 is singled out by the Peliti representation of unity (42). Note that these distributions constitute a 2*D*-dimensional subspace of the space of all distributions on the lattice $\left\{ \textrm{n} \right\}$. It is shown in [69] that these special distributions, because they are minimum-information distributions, have the interpretation of *macrostates* for a general equilibrium or non-equilibrium thermodynamics.

In the framework of [47], the Doi-Peliti integral (43) and other two-field functional integrals are defined on the Pontryagin bundle $TQ \oplus T^{*} \! Q$ of triples $\left( \theta , \dot{n} , n \right)$ in which all three variables are independent. It is only with the reduction to saddle-point trajectories (and only if the Doi-Peliti integral is evaluated in its continuous-time limit, and not with a finite time-step in the inner product (40)), that these triples are reduced to Lagrangian and Hamiltonian descriptions of the same trajectories with no “duality gap”.

#### Coordinate transformations in phase space expressing adjoint duality as time reversal

A large body of work on “fluctuation theorems” [64], although not required to derive the foregoing dualities or their implied conservation laws, is nonetheless helpful in interpreting the meaning of the conserved volumes in Doi-Peliti theory. Fluctuation theorems may be seen as extensions to finite time intervals of the study of instantaneous adjoint duality between the Kolmogorov-backward and -forward evolution [33] under the transition matrix $\textrm{T}_{\textrm{n}{\textrm{n}}^{\prime }}$.

Hatano and Sasa [38] recognized that application of a suitable similarity transform to the transition matrix exchanges the forms of $\textrm{T}_{\textrm{n}{\textrm{n}}^{\prime }}$ and its adjoint: that is, the similarity transform exchanges the forms of the Kolmogorov-backward and -forward equations, making the evolution of distributions appear like that of observables and vice versa. Performing the similarity transform at every moment in an extended-time quadrature of the form (34) has the effect of multiplying each path of the stochastic coordinate $\textrm{n}$ by a weight [65], creating an extended-time generating functional for paths.^1^ The required similarity transform is generally non-locally determined and requires solution for stationary distributions on the whole state space.

An exception arises for cases in which the stationary distributions are minimum-information distributions such as the Poisson (35), for which the phase-space coordinates $\left( \theta , n \right)$ determine all fluctuation moments. In these cases the similarity transform in the potentially-infinite-dimensional state space $\left\{ \textrm{n} \right\}$ can be projected to a coordinate transform in the phase space [69]. Moreover, the required path weights of [65] for all paths are given by a *D*-dimensional, time-local correction to the Liouville function $\mathcal {L}$, making evolution under the adjoint generator appear like ordinary stochastic evolution in reverse time.

One such coordinate transformation, exchanging the roles of coordinate and response fields in phase space, was first used in Doi-Peliti integrals by Baish [11]. Let ${\varvec{n}}$ be the saddle-point value of the field *n* in Eq. (45) in the steady state that would be annihilated by $\textrm{T}_{\textrm{n}{\textrm{n}}^{\prime }}$ at the parameters it possesses at some time. If $\textrm{T}_{\textrm{n}{\textrm{n}}^{\prime }}$ and thus $\mathcal {L}$ is explicitly time-dependent, then the scale factor ${\varvec{n}}$ will generally be different for each time. Define coherent state fields rescaled locally by ${\varvec{n}}$ as

53 $$\begin{aligned} {\phi }^{\dagger }_i {\varvec{n}}_i&\equiv {\varphi }^{\dagger }_i&\frac{ {\phi }_i }{ {\varvec{n}}_i }&\equiv {\varphi }_i . \end{aligned}$$

The form of the action in fields $\left( {\varphi }^{\dagger } , \varphi \right)$ remains as in Eq. (44) but the Liouville function $\mathcal {L}$ is replaced by a possibly-shifted function

54 $$\begin{aligned} \mathcal {\tilde{L}} \! \left( {\varphi }^{\dagger } , \varphi \right) \equiv \mathcal {L} \! \left( {\phi }^{\dagger } , \phi \right) + \sum _i \left( {\varphi }^{\dagger }_i {\varphi }^i \right) d_t \log {\varvec{n}}_i . \end{aligned}$$

If we follow the Baish transformation (53) with a logarithmic transform equivalent to (45), but in the descaled fields,

55 $$\begin{aligned} {\varphi }^{\dagger }_i&\equiv e^{-{\eta }_i} n_i&{\varphi }_i&\equiv e^{{\eta }_i} , \end{aligned}$$

we coordinatize the base distributions in Fig. 1 as exponential families, and the dependence of number *n* on $\theta$ in mixture families. The evaluation of the triangle inequality (12) is unchanged, but the coordinates in which vector fields are expanded are transformed.

The action (44) in the new variables becomes

56 $$\begin{aligned} S = \int _0^T dt \left\{ \left( d_t \eta \right) n + \mathcal {\tilde{L}} \! \left( n , \eta \right) \right\} \end{aligned}$$

where the modified Liouville function $\mathcal {\tilde{L}}$ from Eq. (54) must be used, such that in the new variables

57 $$\begin{aligned} \mathcal {\tilde{L}} \! \left( n , \eta \right) \equiv \mathcal {L} \! \left( \theta , n \right) + \sum _{i = 1}^D n_i d_t \log {\varvec{n}}_i \end{aligned}$$

The symmetric exponential families on phase space represented by the coordinate systems $\left( \theta , n \right)$ from Eq. (45) and $\left( n , \eta \right)$ from Eq. (55) provide natural variables in which to expand the coefficients of affine connections constructed from the $\psi$-divergence below in Sect. 3.3.

## The Liouville theorem connecting dynamics to inference induced by two-field stationary trajectories

In the review of standard results in Sect. 2, we have shown where a phase space with conserved volume under mappings along Hamiltonian stationary trajectories originates within Doi-Peliti theory, and identified a particular form (15) for the volume element preserved by a mapping of coordinate differentials along these trajectories. In this section we compute two quantities that are conserved under time translation and develop their interpretations as information measures. The first is a scalar phase-space density, which we identify as the Wigner function of semiclassical Liouville evolution. The second is an inner product of vector fields, which we express in coordinate-invariant terms by defining affine connections for dual parallel transport compatible with the Riemannian metric (4).

The distinctive feature of the parallel transport derived here is that it is affine-flat in the coherent-state variables of Sect. 2.3.2, rather than in the exponential coordinates $\theta$ normally used to define dually-flat parallel transport, as in Ch. 6 of [3, 54]. The importance-sampling interpretation of Sect. 2.2 identifies a *geometric* role for coherent-state variables that we believe has not been recognized. Dual parallel transport under these affine connections expresses conservation of the Liouville volume element as a result of compensation, in the flow of phase-space trajectories, between the resolution of underlying base distributions and the discrimination imposed by sampling bias. The information available from the large-deviation probability as a sample estimator, about differences between possible initial distributions, is carried by the eigenvalues of the Fisher information metric, which are transported as invariants along stationary trajectories.

The canonical transformation of Sect. 2.3.6, which exchanges the roles of fields representing the base distribution and the sampling bias in the Doi-Peliti integral, is used to express the conserved information volume in symmetric form in terms of the Fisher metric. The symmetric representation of adjoint duality [37, 38] in terms of apparent time-reversal in the Doi-Peliti integral makes clear that the “reversibility” entailed by conserved phase-space volumes in the Hamilton–Jacobi equation should be understood as a reflection of duality between dynamics (the forward propagation of base distributions) and inference (the backward propagation of sample biases).

We begin with the conserved scalar density and its interpretation as a statistical model in the terms of importance sampling, and then define vector fields corresponding to the extended Pythagorean theorem (12) and derive appropriate affine connections for them.

### The Peliti functional integral as a statistical model

The Peliti basis of coherent states (37) defines a *statistical model* for the stochastic process. The role of the projection operators in the representation of unity (42) in populating the model can be clarified by splitting the functional integral (44) at any intermediate time *t*, in the same fashion as the Chapman–Kolmogorov equation splits the time evolution of a probability distribution by a sum over intermediate states. This is done by integrating Eq. (43) up to time *t*, inserting an explicit representation of unity in terms of a pair of fields $\left( {\phi }_{\ddagger }^{\dagger } , {\phi }_{\ddagger } \right)$, and resuming the functional integral on the generating function extracted by that representation of unity:

58 $$\begin{aligned} e^{{\psi }_T \left( \theta \right) } =&\int _{t + \delta t}^T {\mathcal {D}}^D \! {\phi }^{\dagger } {\mathcal {D}}^D \! {\phi } \, e^{ \left( z - {\phi }^{\dagger }_T \right) {\phi }_T - S } \int d^D \! {\phi }_{\ddagger } e^{ \left( {\phi }^{\dagger }_{t + \delta t} - 1 \right) {\phi }_{\ddagger } }\nonumber \\ {}&\times \int \frac{ d^D \! {\phi }^{\dagger }_{\ddagger } }{ {\pi }^D } e^{ - \left( {\phi }^{\dagger }_{\ddagger } - 1 \right) {\phi }_{\ddagger } } e^{ {\psi }_t \left( \log {\phi }^{\dagger }_{\ddagger } \right) } . \end{aligned}$$

The argument $\log {\phi }^{\dagger }_{\ddagger }$ is the CGF coordinate for an exponential family of distributions tilted from whatever base distribution the functional integral produces at time *t*. The inner product ${\phi }^{\dagger }_{t + dt} {\phi }_{\ddagger }$ is a dual mixture coordinate, corresponding to the mean of *n* in the importance distribution with $\left| {\phi }_{\ddagger } \right)$ as the base distribution and ${\phi }^{\dagger }_{t + dt}$ as the likelihood ratio. It is the mean of this importance distribution, together with the log-likelihood that is its dual coordinate in the exponential family, that must transform under symplectomorphism to satisfy the condition (19) for preservation of inner products. We show next that the stationary-path conditions provide the necessary mapping.

### The Wigner function from the two-field identity operator plays the role of a phase-space density

The scalar density in 2FFI that fills the role of a phase space density in classical Hamiltonian mechanics is the Wigner function [76], of which versions exist for both classical and quantum systems.^2^ It is defined in terms of the representation of unity in Eq. (58), as

59 $$\begin{aligned} w_t \! \left( {\phi }^{\dagger }_{\ddagger } , {\phi }_{\ddagger } \right)&\equiv \frac{1}{{\pi }^D} \int _{t + \delta t}^T {\mathcal {D}}^D \! {\phi }^{\dagger } {\mathcal {D}}^D \! {\phi } \, e^{ \left( z - {\phi }^{\dagger }_T \right) {\phi }_T - S } e^{ \left( {\phi }^{\dagger }_{t + \delta t} - {\phi }^{\dagger }_{\ddagger } \right) {\phi }_{\ddagger } } e^{ {\psi }_t \left( \log {\phi }^{\dagger }_{\ddagger } \right) } \end{aligned}$$

Eq. (59) implies that, for $w_t \! \left( {\phi }^{\dagger }_{\ddagger } , {\phi }_{\ddagger } \right)$ at any time,

60 $$\begin{aligned} e^{{\psi }_T \left( \theta \right) } = \int d^D \! {\phi }^{\dagger }_{\ddagger } \, d^D \! {\phi }_{\ddagger } \, w_t \! \left( {\phi }^{\dagger }_{\ddagger } , {\phi }_{\ddagger } \right) \end{aligned}$$

In a saddle-point approximation, one identifies arguments $\left( {\bar{\phi }}^{\dagger }_{\ddagger } , {\bar{\phi }}_{\ddagger } \right)$ for which, to leading exponential order,

61 $$\begin{aligned} e^{{\psi }_T \left( \theta \right) } \sim w_t \! \left( {\bar{\phi }}^{\dagger }_{\ddagger } , {\bar{\phi }}_{\ddagger } \right) \end{aligned}$$

Since Eq. (61) approximates the same function at any time *t*, its total time derivative along the sequence of stationary points must vanish,

62 $$\begin{aligned} 0&= \frac{d}{dt} w_t \! \left( {\bar{\phi }}^{\dagger }_{\ddagger } , {\bar{\phi }}_{\ddagger } \right) = \left( \frac{\partial }{\partial t} + \frac{d{\bar{\phi }}^{\dagger }_{\ddagger }}{dt} \frac{\partial }{\partial {\bar{\phi }}^{\dagger }_{\ddagger }} + \frac{d{\bar{\phi }}_{\ddagger }}{dt} \frac{\partial }{\partial {\bar{\phi }}_{\ddagger }} \right) w_t \! \left( {\bar{\phi }}^{\dagger }_{\ddagger } , {\bar{\phi }}_{\ddagger } \right) \end{aligned}$$

Moreover, the stationary points should coincide with values along the stationary trajectories (47) of the functional integral (58), which satisfy

63 $$\begin{aligned} { \left. \left( \sum _i \frac{ \partial {\dot{\phi }}^{\dagger }_i }{ \partial {\phi }^{\dagger }_i } + \sum _i \frac{ \partial {\dot{\phi }}_i }{ \partial {\phi }_i } \right) \right| }_{{\bar{\phi }}^{\dagger } , \bar{\phi }} = 0 \end{aligned}$$

Equation (62) may thus be recast as the conservation law for a 2*D*-dimensional current $\left( {\dot{\phi }}^{\dagger } w , {\dot{\phi }} w \right)$,

64 $$\begin{aligned} 0 = \frac{\partial w_t}{\partial t} + \sum _i \frac{\partial }{\partial {\phi }^{\dagger }_i} \left( {\dot{\phi }}^{\dagger }_i w_t \right) + \sum _i \frac{\partial }{\partial {\phi }_i} \left( {\dot{\phi }}_i w_t \right) \end{aligned}$$

which is Liouville’s theorem.

$w_t$ is a density of rays for joint base distributions and likelihood ratios that is conserved along the Doi-Peliti stationary trajectories. $\log w_t$ is the leading exponential approximation to the value of the CGF. It therefore integrates information along the trajectory from the final-time imposed value of *z* and the initial-time structure of the generating function ${\psi }_0 \! \left( \log {\phi }^{\dagger }_0 \right)$.

The indirect definition (59) of the Wigner function in terms of the functional integral is convenient to manipulate but perhaps not very self-explanatory. Appendix E gives a direct construction of the stationary-point approximation in terms of a density $\rho \! \left( \theta \right)$ over the basis of coherent states $\left| \phi \right)$ and their Laplace transforms, and verifies that the sequence of stationary points do indeed coincide with the equations of motion (47).

#### Constraints and conserved current flows in reduced dimensions

Often systems of interest will evolve under constraints arising from conservation laws, such as conserved quantities of the stoichiometry in chemical reaction networks [44, 60, 71]. Conserved quantities result in flat directions in the CGF and zero eigenvalues of the Fisher metric. Since generally the constraints will involve multiple species, and because the logarithmic canonical transform (45) is defined in the species basis, it will not be possible to factor out non-dynamical combinations. Then the transport equation (64) for the current of the Wigner density will occupy only a sub-manifold of the 2*D*-dimensional Doi-Peliti coordinate space needed to define the system.

A convenient way to handle constraints is to work in the eigenbasis of the Fisher metric which we will index with subscript $\alpha$, where a number-potential counterpart to the transport equation (64) reads

65 $$\begin{aligned} 0&= \frac{\partial w_t}{\partial t} + \sum _{\alpha } \frac{\partial }{\partial {\theta }^{\alpha }} \left( {\dot{\theta }}^{\alpha } w_t \right) + \sum _{\alpha } \frac{\partial }{\partial n_{\alpha }} \left( {\dot{n}}_{\alpha } w_t \right) \end{aligned}$$

The picture of the Liouville equation as implying a conserved volume element

66 $$\begin{aligned} \frac{d}{dt} \left( \prod _{\alpha } \delta {\theta }^{\alpha } \delta n_{\alpha } \right) = 0 \end{aligned}$$

with the product index $\alpha$ taken only over nonzero eigenvalues of the Fisher metric, remains nondegenerate and has a direct interpretation in terms of the product of eigenvectors of the Fisher inner product in independent dimensions.

### The Fisher metric and cubic tensor in dual canonical coordinates

The leading-exponential equivalence of the Wigner density to the CGF from Eq. (61) suggests that the 2*D*-dimensional differential of the stationary-point CGF should likewise obey a symplectic transport law, implying a transport law for the Fisher metric. To derive those results we return to the expression of the differential of the CGF in terms of the generalized Pythagorean theorem (12), and derive the Fisher metric from the $\psi$-divergence following Amari [2, Sec. 6.2].

Base distributions corresponding to points along stationary paths under the action (44) form exponential families, because they are in the class of coherent-state distributions described in Appendix A.2. Therefore label importance distributions (6) symmetrically as $\tilde{\rho } \! \left( \theta , \eta \right)$ with the exponential coordinates in the two logarithmic transformations (45) and (55). To study their independent variations about a reference value $\left( {\theta }_R , {\eta }_R \right)$, introduce two distinct exponential families, labeled

67 $$\begin{aligned} \rho&\equiv { \tilde{\rho } \! \left( \theta , \eta \right) }_{\eta = {\eta }_R}&{\rho }^{\prime }&\equiv { \tilde{\rho } \! \left( \theta , \eta \right) }_{\theta = {\theta }_R} \end{aligned}$$

The $\psi$-divergence $D_{\psi } \! \left( \theta : \eta \right) = \psi \! \left( \theta \right) + {\psi }^{*} \! \left( n \right) - n \theta$, a Bregman divergence of the CGF, is related to the Kullback–Leibler divergence of ${\rho }^{\prime }$ from $\rho$ as

68 $$\begin{aligned} D_{\psi } \! \left( \theta : \eta \right)&= D_\textrm{KL} \! \left( {\rho }^{\prime } \parallel \rho \right) \nonumber \\&= \sum _{\textrm{n}} {\tilde{\rho }}_{\textrm{n}} \! \left( {\theta }_R , \eta \right) \log \left( \frac{ {\tilde{\rho }}_{\textrm{n}} \! \left( {\theta }_R , \eta \right) }{ {\tilde{\rho }}_{\textrm{n}} \! \left( \theta , {\eta }_R \right) } \right) \end{aligned}$$

The mixed second partial derivative of $- D_{\psi }$ gives the same variance that defines the Fisher metric. At general $\theta$, $\eta$, it is labeled

69 $$\begin{aligned} g^D_{ij}&= - \frac{\partial }{\partial {\theta }^i} \frac{\partial }{\partial {\eta }^j} D_{\psi } \! \left( \theta : \eta \right) \nonumber \\&= \sum _{\textrm{n}} \frac{ \partial {\tilde{\rho }}_{\textrm{n}} \! \left( {\theta }_R , \eta \right) }{ \partial {\eta }^j } \frac{ \partial \log {\tilde{\rho }}_{\textrm{n}} \! \left( \theta , {\eta }_R \right) }{ \partial {\theta }^i } \nonumber \\&= \sum _{\textrm{n}} {\tilde{\rho }}_{\textrm{n}} \! \left( {\theta }_R , \eta \right) \left( {\textrm{n}}_j - \frac{\partial \psi }{\partial {\eta }^j} \right) \left( {\textrm{n}}_i - \frac{\partial \psi }{\partial {\theta }^i} \right) \end{aligned}$$

At $\theta = {\theta }_R$, $\eta = {\eta }_R$, the second line of Eq. (69) recovers exactly the differential form of the Pythagorean theorem of Eq. (12) in dual exponential coordinates.

Two third-order mixed partials define the connection coefficients for Amari’s dually-flat connections on exponential and mixture coordinates. Written in all-contravariant indices,^3^ these are given by

70 $$\begin{aligned} {\Gamma }^D_{kij}&= \frac{\partial }{\partial {\theta }^k} g^D_{ij} \nonumber \\&= - \frac{ {\partial }^2 \psi }{ \partial {\theta }^k \partial {\theta }^i } \sum _{\textrm{n}} {\tilde{\rho }}_{\textrm{n}} \! \left( {\theta }_R , \eta \right) \left( {\textrm{n}}_j - \frac{\partial \psi }{\partial {\eta }^j} \right) \nonumber \\ {\Gamma }^{D*}_{kij}&= \frac{\partial }{\partial {\eta }^k} g^D_{ij} \nonumber \\&= \sum _{\textrm{n}} {\tilde{\rho }}_{\textrm{n}} \! \left( {\theta }_R , \eta \right) { \left( {\textrm{n}}_j - \frac{\partial \psi }{\partial {\eta }^j} \right) }^2 \left( {\textrm{n}}_i - \frac{\partial \psi }{\partial {\theta }^i} \right) \nonumber \\ {}&\quad - \frac{ {\partial }^2 \psi }{ \partial {\eta }^k \partial {\eta }^j } \sum _{\textrm{n}} {\tilde{\rho }}_{\textrm{n}} \! \left( {\theta }_R , \eta \right) \left( {\textrm{n}}_i - \frac{\partial \psi }{\partial {\theta }^i} \right) \end{aligned}$$

Evaluated at $\theta = {\theta }_R$ and $\eta = {\eta }_R$,

71 $$\begin{aligned} g^D_{ij} \! \left( {\theta }_R , {\eta }_R \right)&= g_{ij} = { \left. \frac{ {\partial }^2 \psi }{ \partial {\theta }^i \partial {\theta }^j } \right| }_{{\theta }_R, {\eta }_R} \nonumber \\ {\Gamma }^{D}_{kij} \! \left( {\theta }_R , {\eta }_R \right)&= 0 \nonumber \\ {\Gamma }^{D*}_{kij} \! \left( {\theta }_R , {\eta }_R \right)&= T_{kij} = { \left. \frac{ {\partial }^3 \psi }{ \partial {\theta }^k \partial {\theta }^i \partial {\theta }^j } \right| }_{{\theta }_R , {\eta }_r} \end{aligned}$$

$g_{ij}$ is the Fisher metric introduced in Eq. (4), and $T_{kij}$ is the cubic tensor, also called the Amari–Chentsov tensor [2]. Below we remove the subscript *R* and write $\theta$ and $\eta$ as the arguments of these tensors.

### The dual vector fields induced by base-distribution initial conditions, and final-time tilts

From the construction of $g^D$ in Sect. 3.3, we can see how to use the stationary-path equations of motion to induce two mappings of vector fields that respect the dual arguments of the $\psi$-divergence. Variations in the likelihood act on the $\rho$ argument, while variations in the base distribution act on the ${\rho }^{\prime }$ argument, in Eq. (67). The stationary-path equations are then used to define a 1-parameter family of maps of any basis of dual variations in initial base and final tilt parameters to intermediate times. Conservation of the Liouville volume then translates to a conserved inner product of pairs of vector fields transported respectively under the two branches of the dual mapping. Conservation of the inner product will imply a transport equation for the Fisher metric corresponding to the Eq. (65) for the Wigner function.

Introduce two vector fields corresponding to variations in $\theta$ at the final time *T*, and to variations in $\eta$ at the initial time 0. The first can be independently imposed through the arguments in ${\Psi }_T \! \left( z \right)$, while the second can be independently imposed in the initial data. Fields $\delta {\theta }_T$ and $\delta {\eta }_0$ are written in components as

72 $$\begin{aligned} \delta {\theta }_T&\equiv \delta {\theta }_T^i \frac{\partial }{\partial {\theta }_T^i}&\delta {\eta }_0&\equiv \delta {\eta }_0^i \frac{\partial }{\partial {\eta }_0^i} \end{aligned}$$

The stationary-path conditions map the dual initial and final coordinates to pairs of coordinates at any intermediate time, which we denote $\theta \! \left( {\theta }_T, {\eta }_0, t \right)$, $\eta \! \left( {\theta }_T, {\eta }_0, t \right)$. A one-parameter family of vector fields is defined by assigning to each such coordinate image $\left( \theta \! \left( {\theta }_T, {\eta }_0, t \right) , \eta \! \left( {\theta }_T, {\eta }_0, t \right) \right)$ the field values

73 $$\begin{aligned} \delta \theta \! \left( \theta , \eta , t \right)&\equiv \delta {\theta }_T^i \frac{\partial }{\partial {\theta }_T^i} \theta \! \left( {\theta }_T, {\eta }_0, t \right)&\delta \eta \! \left( \theta , \eta , t \right)&\equiv \delta {\eta }_0^i \frac{\partial }{\partial {\eta }_0^i} \eta \! \left( {\theta }_T, {\eta }_0, t \right) \end{aligned}$$

Under the change of coordinates from $\left( {\theta }_T, {\eta }_0 \right)$ to $\left( \theta , \eta \right)$ at each time *t*, the vector fields (73) may be written in terms of the local coordinate differentials as

74 $$\begin{aligned} \delta \theta \! \left( \theta , \eta , t \right)&\equiv \delta {\theta }^j \! \left( \theta , \eta , t \right) \frac{\partial }{\partial {\theta }^j}&\delta \eta \! \left( \theta , \eta , t \right)&\equiv \delta {\eta }^j \! \left( \theta , \eta , t \right) \frac{\partial }{\partial {\eta }^j} \end{aligned}$$

Below we suppress the explicit $\left( \theta , \eta , t \right)$ coordinate and time arguments of $\delta {\theta }^j \! \left( \theta , \eta , t \right)$ and $\delta \eta \! \left( \theta , \eta , t \right)$, and indicate the time *t* in a subscript only where it is needed to avoid confusion.

The vector fields (74) have a time dependence that can be defined through the dependences of $\left( \theta \! \left( {\theta }_T, {\eta }_0, t \right) , \eta \! \left( {\theta }_T, {\eta }_0, t \right) \right)$ on the boundary coordinates and then transformed to the local coordinate system, becoming

75 $$\begin{aligned} { \left( \frac{d}{dt} \delta \theta \right) }^j&= \delta {\theta }_T^i \frac{\partial }{\partial {\theta }_T^i} {\dot{\theta }}^j \! \left( {\theta }_T, {\eta }_0, t \right) \nonumber \\&= \delta {\theta }_t^i \frac{\partial }{\partial {\theta }_t^i} {\dot{\theta }}^j \! \left( {\theta }_T, {\eta }_0, t \right) \nonumber \\&= \delta {\theta }_t^i \frac{ {\partial }^2 \mathcal {L} }{ \partial {\theta }^i \partial n_j } \nonumber \\ { \left( \frac{d}{dt} \delta \eta \right) }^j&= \delta {\eta }_0^i \frac{\partial }{\partial {\eta }_0^i} {\dot{\eta }}^j \! \left( {\theta }_T, {\eta }_0, t \right) \nonumber \\&= \delta {\eta }_t^i \frac{\partial }{\partial {\eta }_t^i} {\dot{\eta }}^j \! \left( {\theta }_T, {\eta }_0, t \right) \nonumber \\&= - \delta {\eta }_t^i \frac{ {\partial }^2 \mathcal {\tilde{L}} }{ \partial {\eta }^i \partial n_j } \end{aligned}$$

Eq. (48) is used to arrive at the third form of each equation in terms of mixed partials of $\mathcal {L}$ and $\mathcal {\tilde{L}}$.

The coordinate transformation (7) from contravariant exponential coordinates to covariant mixture coordinates may be used in two ways to write the inner product of vector fields $\delta \theta$ and $\delta \eta$ in mixed form. From the definition of the inner product in terms of $g^D$ in Eq. (69) and its equivalence to the Hessian definition of *g* in Eq. (71),

76 $$\begin{aligned} {\left( \delta \theta \right) }^i g_{ij} {\left( \delta \eta \right) }^j&\equiv {\left( \delta \theta \right) }^i {\left( {\delta }_{\eta } n \right) }_i \equiv {\left( {\delta }_{\theta } n \right) }_j {\left( \delta \eta \right) }^j \end{aligned}$$

Although the field variable *n* is the same in either log-transform (45) or (55), the two displacements ${\delta }_{\eta } n$ and ${\delta }_{\theta } n$ are independent vector fields.

#### The conserved inner product of dual vector fields, and directional transport of the metric

Equation (75) has a symmetric form but evolves $\delta \theta$ and $\delta \eta$ respectively using $\mathcal {L}$ and $\mathcal {\tilde{L}}$, making it not immediately apparent that the inner product is preserved. Writing the field $\delta \eta$ in its dual mixture coordinate as in the first line of Eq. (76) the time derivative becomes

77 $$\begin{aligned} { \left( \frac{d}{dt} \delta n \right) }_i&= \delta n_{0j} \frac{\partial }{\partial n_{0j}} {\dot{n}}_i \! \left( {\theta }_T, n_0, t \right) \nonumber \\&= \delta n_{tj} \frac{\partial }{\partial n_{tj}} {\dot{n}}_i \! \left( {\theta }_T, n_0, t \right) \nonumber \\&= - \delta n_{tj} \frac{ {\partial }^2 \mathcal {L} }{ \partial n_j \partial {\theta }^i } \end{aligned}$$

The condition (18) is met and we have

78 $$\begin{aligned} { \left( \frac{d}{dt} \delta {\theta } \right) }^j {\left( \delta n \right) }_j + {\left( \delta \theta \right) }^i { \left( \frac{d}{dt} \delta n \right) }_i = 0 \end{aligned}$$

Using Eq. (78) to evaluate the change in the inner product written as ${\left( \delta \theta \right) }^i g_{ij} {\left( \delta \eta \right) }^j$, substituting the derivatives (48) for $\dot{\theta }$ and $\dot{\eta }$, and grouping terms, we obtain the transport equation for the metric along stationary paths

79 $$\begin{aligned} 0&= {\dot{\theta }}^k \frac{\partial g_{ij}}{\partial {\theta }^k} + \frac{ {\partial }^2 \mathcal {L} }{ \partial {\theta }^i \partial n_k } g_{kj} + \frac{\partial g_{ij}}{\partial {\eta }^k} {\dot{\eta }}^k - g_{ik} \frac{ {\partial }^2 \mathcal {\tilde{L}} }{ \partial n_k \partial {\eta }^j } \nonumber \\&= \left( \frac{ \partial \mathcal {L} }{ \partial n_k } \frac{\partial g_{ij}}{\partial {\theta }^k} + \frac{ {\partial }^2 \mathcal {L} }{ \partial {\theta }^i \partial n_k } g_{kj} \right) - \left( \frac{\partial g_{ij}}{\partial {\eta }^k} \frac{ \partial \mathcal {\tilde{L}} }{ \partial n_k } + g_{ik} \frac{ {\partial }^2 \mathcal {\tilde{L}} }{ \partial n_k \partial {\eta }^j } \right) \nonumber \\&= \frac{ \partial }{ \partial {\theta }^i } \left( {\dot{\theta }}^k g_{kj} \right) + \frac{ \partial }{ \partial {\eta }^j } \left( g_{ik} {\dot{\eta }}^k \right) \end{aligned}$$

The tensor transport equation from Eq. (79) can be compared to Eq. (65) for the transport of the Wigner density.

### Dual connections respecting the symplectic structure of canonical transformations in the two-field system

The transport relations derived so far make use of the symplectic structure of maps generated by time-translation along Doi-Peliti stationary paths, but they are not specifically geometric. We now turn to geometric constructions that respect the symplectic structure, render its maps coordinate invariant under canonical transformations, and express the special roles of affine transport in some coordinates such as coherent states through the definition of appropriate Riemannian connections.

#### Conservation of the inner product through the combined effects of two maps

The inner product (76) is preserved through the complementary action of two maps, one generated by the time-dependence of $\theta$, and the other by the time-dependence of $\eta$. By construction, $\delta \theta$ depends on time only through $\dot{\theta }$, and $\delta \eta$ only through $\dot{\eta }$, while the metric has no explicit time dependence but changes under both maps as the location $\left( \theta , \eta \right)$ changes. Denoting by ${ \left. d / dt \right| }_{\dot{\theta }}$ and ${ \left. d / dt \right| }_{\dot{\eta }}$ these separate components of change, the time derivative of the inner product can be partitioned into two canceling terms:

80 $$\begin{aligned} { \left( { \left. \frac{d}{dt} \right| }_{\dot{\theta }} {\delta }_{\theta } n \right) }_j {\left( \delta \eta \right) }^j&\equiv \left[ { \left( \frac{d}{dt} \delta \theta \right) }^i g_{ij} + {\left( \delta \theta \right) }^i {\dot{\theta }}^k \frac{\partial g_{ij}}{\partial {\theta }^k} \right] {\left( \delta \eta \right) }^j \nonumber \\ {\left( \delta \theta \right) }^i { \left( { \left. \frac{d}{dt} \right| }_{\dot{\eta }} {\delta }_{\eta } n \right) }_i&\equiv {\left( \delta \theta \right) }^i \left[ g_{ij} { \left( \frac{d}{dt} \delta \eta \right) }^j + {\dot{\eta }}^k \frac{\partial g_{ij}}{\partial {\eta }^k} {\left( \delta \eta \right) }^j \right] \end{aligned}$$

Connection coefficients may be added within either ${\left( { \left. d / dt \right| }_{\dot{\theta }} \, {\delta }_{\theta } n \right) }_j$ or ${\left( { \left. d / dt \right| }_{\dot{\eta }} \, {\delta }_{\eta } n \right) }_i$ to make the components of change in the vector field and metric coordinate-invariant, without altering the duality between independent variations in the base distribution and in the likelihood ratio.

To introduce a connection we first replace the total derivative *d*/*dt* with a partial-derivative decomposition expressing the same transformation as a flow:

81 $$\begin{aligned} { \left( \frac{d}{dt} \delta \theta \right) }^i&\equiv { \left( \frac{\partial }{\partial t} \delta \theta \right) }^i + {\dot{\theta }}^k \frac{\partial }{\partial {\theta }^k} {\left( \delta \theta \right) }^i + {\dot{\eta }}^k \frac{\partial }{\partial {\eta }^k} {\left( \delta \theta \right) }^i \nonumber \\ { \left( \frac{d}{dt} \delta \eta \right) }^j&\equiv { \left( \frac{\partial }{\partial t} \delta \eta \right) }^j + {\dot{\theta }}^k \frac{\partial }{\partial {\theta }^k} {\left( \delta \eta \right) }^j + {\dot{\eta }}^k \frac{\partial }{\partial {\eta }^k} {\left( \delta \eta \right) }^j \end{aligned}$$

Connection coefficients are defined from the pullbacks ${\left( \partial / \partial {\theta }^k \right) }^{\prime }$ or ${\left( \partial / \partial {\eta }^k \right) }^{\prime }$ of infinitesimally transformed basis vectors in the tangent spaces to the two exponential families,

82 $$\begin{aligned} \frac{\partial }{\partial {\theta }^k} { \left( \frac{\partial }{\partial {\theta }^j} \right) }^{\prime }&\equiv {{\Gamma }^{\left( \theta \right) }_{kj}}^i \left( \frac{\partial }{\partial {\theta }^i} \right)&\frac{\partial }{\partial {\theta }^k} { \left( \frac{\partial }{\partial {\eta }^j} \right) }^{\prime }&\equiv {{\Gamma }^{\left( \eta \right) }_{kj}}^i \left( \frac{\partial }{\partial {\eta }^i} \right) \nonumber \\ \frac{\partial }{\partial {\eta }^k} { \left( \frac{\partial }{\partial {\eta }^j} \right) }^{\prime }&\equiv {{\Gamma }^{\left( \eta \right) *}_{kj}}^i \left( \frac{\partial }{\partial {\eta }^i} \right)&\frac{\partial }{\partial {\eta }^k} { \left( \frac{\partial }{\partial {\theta }^j} \right) }^{\prime }&\equiv {{\Gamma }^{\left( \theta \right) *}_{kj}}^i \left( \frac{\partial }{\partial {\theta }^i} \right) \end{aligned}$$

Superscripts ${\Gamma }^{\left( \theta \right) }$ or ${\Gamma }^{\left( \eta \right) }$ refer to the subspace of basis vectors $\partial / \partial \theta$ or $\partial / \partial \eta$ being pulled back, and the designation $\Gamma$ or ${\Gamma }^{*}$ distinguishes the connection associated with $\theta$ displacement or $\eta$ displacement, respectively. Because the $\dot{\eta }$ component of time translation does not act in $\delta \theta$ and vice versa, we set connection coefficients ${{\Gamma }^{\left( \eta \right) }_{kj}}^i$ and ${{\Gamma }^{\left( \theta \right) *}_{kj}}^i$ to zero.

Covariant derivatives of the vector fields $\delta \theta$ and $\delta \eta$ in the connections $\Gamma$, ${\Gamma }^{*}$ of Eq. (82) are defined as

83 $$\begin{aligned} { \left( {\nabla }^{\left( \theta \right) }_k \delta \theta \right) }^i&= \frac{\partial }{\partial {\theta }^k} { \left( \delta \theta \right) }^i + {{\Gamma }^{\left( \theta \right) }_{kj}}^i { \left( \delta \theta \right) }^j&{ \left( {\nabla }^{\left( \eta \right) }_k \delta \theta \right) }^i&= \frac{\partial }{\partial {\eta }^k} { \left( \delta \theta \right) }^i \nonumber \\ { \left( {\nabla }^{\left( \theta \right) *}_k \delta \eta \right) }^j&= \frac{\partial }{\partial {\theta }^k} { \left( \delta \eta \right) }^j&{ \left( {\nabla }^{\left( \eta \right) *}_k \delta \eta \right) }^j&= \frac{\partial }{\partial {\eta }^k} { \left( \delta \eta \right) }^j + {{\Gamma }^{\left( \eta \right) *}_{ki}}^j { \left( \delta \eta \right) }^i \end{aligned}$$

The covariant part of the flow decomposition in Eq. (81) is defined by subtraction of the nonzero connection coefficients from the total derivatives (75), as

84 $$\begin{aligned} { \left( \frac{\partial }{\partial t} \delta \theta \right) }^j + {\dot{\theta }}^k { \left( {\nabla }^{\left( \theta \right) }_k \delta \theta \right) }^j + {\dot{\eta }}^k { \left( {\nabla }^{\left( \eta \right) }_k \delta \theta \right) }^j&\equiv { \left( \frac{d}{dt} \delta \theta \right) }^j + {\dot{\theta }}^k {{\Gamma }^{\left( \theta \right) }_{ki}}^j { \left( \delta \theta \right) }^i \nonumber \\ { \left( \frac{\partial }{\partial t} \delta \eta \right) }^j + {\dot{\theta }}^k { \left( {\nabla }^{\left( \theta \right) *}_k \delta \eta \right) }^j + {\dot{\eta }}^k { \left( {\nabla }^{\left( \eta \right) *}_k \delta \eta \right) }^j&\equiv { \left( \frac{d}{dt} \delta \eta \right) }^j + {\dot{\eta }}^k {{\Gamma }^{\left( \eta \right) *}_{ki}}^j { \left( \delta \eta \right) }^i \end{aligned}$$

Compensating covariant derivatives of the metric are

85 $$\begin{aligned} {\nabla }^{\left( \theta \right) }_k g_{ij}&= \frac{\partial }{\partial {\theta }^k} g_{ij} - {{\Gamma }^{\left( \theta \right) }_{ki}}^l g_{lj}&{\nabla }^{\left( \eta \right) *}_k g_{ij}&= \frac{\partial }{\partial {\eta }^k} g_{ij} - {{\Gamma }^{\left( \eta \right) *}_{kj}}^l g_{il} \end{aligned}$$

Equation (84) extracts a coordinate-invariant component of the time derivative of vector fields $\delta \theta$ and $\delta \eta$ under canonical transformations, while Eq. (85) extracts the corresponding coordinate-invariant part of the change in the Fisher metric.

#### Referencing arbitrary dual connections to dually flat connections in the exponential family

The manifold for a Doi-Peliti system with *D* independent components has dimension 2*D*, with parallel subspaces for the base distribution and likelihood ratio. The dual connections (82) act within these two independent subspaces, in contrast to the dually-flat connections ${\Gamma }^D$ and ${\Gamma }^{D*}$ of Eq. (70), which act within the same *D*-dimensional exponential family. Although the Fisher metric is a function only of the overall importance distribution, which aggregates dependence from the base distribution and likelihood, the symplectic transformations from translation along stationary paths separate components of variation from within the two independent subspaces. The subspace decomposition cannot be recovered from the importance distribution alone, and thus no connection defined only from the properties of the Fisher metric is sufficient to identify the dual symplectic connections for a Doi-Peliti system.

Nonetheless, we may relate the symplectic dual connections to Amari’s dually flat connections and the Amari–Chentsov tensor through the relation (see [2, Eq. (6.27)])

86 $$\begin{aligned} {\partial }_k g^D_{ij} = {\Gamma }^D_{kij} + {\Gamma }^{D *}_{kji} = 0 + T_{kji} \end{aligned}$$

Substituting Eq. (86) into Eq. (85) gives expressions for the dual covariant derivatives of the Fisher metric

87 $$\begin{aligned} {\nabla }^{\left( \theta \right) }_k g_{ij}&= T_{kji} - {\Gamma }^{\left( \theta \right) }_{kij}&{\nabla }^{\left( \eta \right) *}_k g_{ij}&= T_{kji} - {\Gamma }^{\left( \eta \right) *}_{kji} \end{aligned}$$

#### Flat connections for coherent-state coordinates

Of particular interest in Doi-Peliti theory will be the canonical transformations (45) and (55) between coherent-state and number-potential coordinates. We note that the forms of the connection coefficients for which affine transport in fields ${\phi }^{\dagger }$ is flat in the likelihood subspace, and affine transport in fields $\varphi$ is flat in the base-distribution subspace, are^4^

89 $$\begin{aligned} {{\Gamma }^{\left( \theta \right) }_{kj}}^i&= \frac{\partial {\theta }^i}{\partial {\phi }^{\dagger l}} \frac{\partial }{\partial {\theta }^k} \left( \frac{\partial {\phi }^{\dagger l}}{\partial {\theta }^j} \right)&{{\Gamma }^{\left( \eta \right) }_{kj}}^i&= 0 \nonumber \\ {{\Gamma }^{\left( \theta \right) *}_{kj}}^i&= 0&{{\Gamma }^{\left( \eta \right) *}_{kj}}^i&= \frac{\partial {\eta }^i}{\partial {\varphi }^l} \frac{\partial }{\partial {\eta }^k} \left( \frac{\partial {\varphi }^l}{\partial {\eta }^j} \right) \end{aligned}$$

### On the roles of coherent-state versus number-potential coordinates in the Doi-Peliti representation

The Doi-Peliti solution method is almost always introduced through the coherent-state representation [9, 42, 53], and for many applications such as chemical reaction networks [8, 10, 44, 71] or evolutionary population processes [70], coherent states are also the “native” representation in the sense that the Liouville operator is a finite-order (generally low-order) polynomial in fields. Moreover, for the importance-sampling interpretation emphasized in this paper, the coherent-state representation separates the nominal distribution and likelihood ratio.

On the other hand, Legendre duality is defined with respect to potential fields, which are the tilt coordinates $\theta$ in the exponential family of importance distributions, and it is in these coordinates, not the coherent-state coordinates, that the Fisher metric corresponds to the Hessian of the CGF. Indeed, it is not generally possible to define a dual coordinate system from the Hessian of the CGF in coherent-state fields, as we illustrate for the worked example in Sect. 4.5.

The use of Riemannian connections neatly expresses the role of each coordinate system. The elementary eigenvalues of divergence or convergence of bases and tilts, and of information susceptibilities, are often simple in coherent-state coordinates, where they are eigenvalues of coordinate divergence or convergence. In the dual connections (90), covariant derivatives retain these elementary eigenvalues, while inheriting from the exponential family the Fisher geometry that defines contravariant/covariant coordinate duality. A concrete example is given in the next section.

## A worked example: the two-state linear system

The foregoing constructions are nicely illustrated in minimal form in a simple, exactly solvable model. It is the stochastic process for *N* independent random walkers on a network with two states and bidirectional hopping between them. The statistical mechanics of transients, time-dependent generating functionals, and large deviations for this system has been didactically covered within the Doi-Peliti framework in [68]. Though simple, the model is nonetheless rich enough to illustrate the complementary roles of coherent states and number-potential coordinates in Doi-Peliti theory—the former as the “native” coordinates in which the system is simple, and the latter as the coordinate system carrying the Fisher geometry—and the way this relation is captured by the dual coherent-state connection (90) different from both the Levi-Civita connection and the dually-flat connections (71) of Nagaoka and Amari [3, 54].

### Two-argument and one-argument generating functions on distributions with a conserved quantity

The two-state model describes (for example) a one-particle chemical reaction in a well-mixed reactor with the schema

91 $$\begin{aligned} a \overset{ {\varvec{k}}_{+} }{ \underset{ {\varvec{k}}_{-} }{ \rightleftharpoons } } b \end{aligned}$$

The probability per unit time for a reaction event is given by rate constants ${\varvec{k}}_{+}$ and ${\varvec{k}}_{-}$, and proportional sampling (the microphysics underlying mass-action rate laws).

A distribution initially in binomial form (36) will retain that form at all times under the master equation for the schema (91), even with time-dependent coefficients. Here for simplicity we will take ${\varvec{k}}_{+}$ and ${\varvec{k}}_{-}$ to be fixed. Therefor the distribution at any time is specified by descaled mean values ${\nu }_a = \left\langle {\textrm{n}}_a \right\rangle / N$, ${\nu }_b = \left\langle {\textrm{n}}_b \right\rangle / N$, with ${\nu }_a + {\nu }_b = 1$.

Although the system has only one dynamical degree of freedom, it is instructive to compute both the two-argument generating function with independent weights $z_a$ on ${\textrm{n}}_a$ and $z_b$ on ${\textrm{n}}_b$, and the 1-argument generating function for the difference coordinate $\textrm{n}\equiv \left( {\textrm{n}}_b - {\textrm{n}}_a \right) / 2$, to illustrate the role of conservation laws and the geometry of the coherent-state connection. The two-argument CGF (1) for the binomial distribution is

92 $$\begin{aligned} e^{\psi \left( \log z \right) } \equiv \sum _{\textrm{n}} z_a^{{\textrm{n}}_a} z_b^{{\textrm{n}}_b} {\rho }_{{\textrm{n}}_a , {\textrm{n}}_b} = { \left[ z_a {\nu }_a + z_b {\nu }_b \right] }^N \end{aligned}$$

Because the total number $N = \left( {\textrm{n}}_b + {\textrm{n}}_a \right)$ is fixed, the normalized 1-variable distribution may be written

93 $$\begin{aligned} {\rho }_{\textrm{n}} = {\sqrt{{\nu }_b {\nu }_a}}^N \left( \begin{array}{c} N \\ n \end{array} \right) { \left( \frac{{\nu }_b}{{\nu }_a} \right) }^{\textrm{n}} \end{aligned}$$

and the terms in the generating function (92) regrouped as

94 $$\begin{aligned} e^{\psi \left( \log z \right) }&= {\sqrt{z_b z_a}}^N \sum _{\textrm{n}} {\rho }_{\textrm{n}} { \left( \frac{z_b}{z_a} \right) }^{\textrm{n}} \end{aligned}$$

Introducing rotated coordinates on the exponential family of tilts

95 $$\begin{aligned} h&\equiv \left( {\theta }_b + {\theta }_a \right) / 2&\theta&\equiv \left( {\theta }_b - {\theta }_a \right) \end{aligned}$$

and dividing the two-argument MGF (94) by ${\sqrt{z_a z_b}}^N$, we obtain an expression for the one-argument MGF in the difference coordinate $\textrm{n}$:

96 $$\begin{aligned} e^{ \psi \left( \log z \right) - N h }&= \sum _{\textrm{n}} {\rho }_{\textrm{n}} e^{\theta \textrm{n}} \end{aligned}$$

In what follows, $\psi \left( \log z \right)$ will always be used to refer to the two-argument CGF (92), and the 1-argument generating function, when needed, will be written out explicitly as $\psi \! \left( \log z \right) - N h$, as in Eq. (96).

### Generator and conserved volume element in coherent-state coordinates

The master equation for the two-state system is developed in [68], but introduces further notation, and will not be needed here. We move directly to the expression for the Liouville function of Eq. (44) after conversion to field variables, which is

97 $$\begin{aligned} \mathcal {L} = {\varvec{k}}_{+} \left( {\phi }^{\dagger }_a - {\phi }^{\dagger }_b \right) {\phi }_a + {\varvec{k}}_{-} \left( {\phi }^{\dagger }_b - {\phi }^{\dagger }_a \right) {\phi }_b \end{aligned}$$

In what follows, math boldface will be reserved for parameters in the generator such as ${\varvec{k}}_{\pm }$ or functions of these such as the associated steady states used in Eq. (53).

Two descalings reduce the problem to parameters which are dimensionless ratios. The first defines a time coordinate $\tau$ in units of the sum of rate parameters,

98 $$\begin{aligned} \frac{d\tau }{dt} \equiv {\varvec{k}}_{+} + {\varvec{k}}_{-} \end{aligned}$$

The second expresses the equilibrium steady state under generator (97) in terms of relative hopping rates,

99 $$\begin{aligned} \frac{{\varvec{k}}_{+}}{{\varvec{k}}_{+} + {\varvec{k}}_{-}}&= \frac{{\varvec{n}}_b}{N} \equiv {\varvec{\nu }}_b&\frac{{\varvec{k}}_{-}}{{\varvec{k}}_{+} + {\varvec{k}}_{-}}&= \frac{{\varvec{n}}_a}{N} \equiv {\varvec{\nu }}_a \end{aligned}$$

As for the discrete index $\textrm{n}$, define ${\varvec{\nu }} \equiv \left( {\varvec{\nu }}_b - {\varvec{\nu }}_a \right) / 2$.

Conservation of total number *N* results in a generator $\mathcal {L}$ that is a function only of the difference variable $\left( {\phi }^{\dagger }_b - {\phi }^{\dagger }_a \right)$. Therefore it is natural to rotate the coherent-state fields to components corresponding to conserved *N* and dynamical $\textrm{n}/ N$, and their dual coordinates in the generating-function argument *z*:

100 $$\begin{aligned} {\phi }^{\dagger }&\equiv {\phi }^{\dagger }_b - {\phi }^{\dagger }_a&\hat{\phi }&\equiv \left( {\phi }_b - {\phi }_a \right) / 2N \nonumber \\ {\Phi }^{\dagger }&\equiv \left( {\phi }^{\dagger }_b + {\phi }^{\dagger }_a \right) / 2 .&\hat{\Phi }&\equiv \left( {\phi }_b + {\phi }_a \right) / N \end{aligned}$$

In rotated fields (100) the action (44) becomes

101 $$\begin{aligned} S= & {} N \int d\tau \left[ - {\partial }_{\tau } {\Phi }^{\dagger } \hat{\Phi } - {\partial }_{\tau } {\phi }^{\dagger } \hat{\phi } + {\phi }^{\dagger } \left( \hat{\phi } - {\varvec{\nu }} \hat{\Phi } \right) \right] \nonumber \\\equiv & {} N \int d\tau \left( - {\partial }_{\tau } {\Phi }^{\dagger } \hat{\Phi } - {\partial }_{\tau } {\phi }^{\dagger } \hat{\phi } + \hat{\mathcal {L}} \right) \end{aligned}$$

A descaled Liouville function has been introduced as $N \left( {\varvec{k}}_{+} + {\varvec{k}}_{-} \right) \hat{\mathcal {L}} \equiv \mathcal {L}$. Absence of the field ${\Phi }^{\dagger }$ from $\hat{\mathcal {L}}$ implies constancy of the expectation for $\hat{\Phi }$.^5^

#### Splitting the symplectic structure between coherent-state conjugate field pairs

Although $\hat{\Phi }$ obeys certain time-translation invariances in correlation functions, its value even along stationary paths will not generally be 1. Therefore the coherent-state variables cannot directly be interpreted as mean values of number variables in the nominal distribution or mean weights in its dual likelihood ratio. To express the functions that are these expectation values, we recall the mean number components in the importance distribution, which are bilinear quantities in ${\phi }^{\dagger }$ and $\phi$, and then introduce a pair of dual number coordinates that, while not linear functions of the coherent-state fields, are functions respectively of ${\phi }^{\dagger }$ or of $\phi$ extracted by making use of the steady-state measure under the instantaneous value ${\varvec{\nu }}$ in the generator (97). (Along stationary paths, where some components of ${\phi }^{\dagger }$ or $\phi$ are invariant, these dual number fields will become linear functions of the remaining dynamical components of ${\phi }^{\dagger }$ or $\phi$, as we show below.)

The two components of the normalized number field in number-potential coordinates (45) are given by

102 $$\begin{aligned} \frac{1}{N} {\phi }^{\dagger }_b {\phi }_b&\equiv {\nu }_b \equiv \left( \frac{1}{2} + \nu \right)&\frac{1}{N} {\phi }^{\dagger }_a {\phi }_a&\equiv {\nu }_a \equiv \left( \frac{1}{2} - \nu \right) \end{aligned}$$

Recall that the instantaneous steady state under the generating process is the scale variable for the dualizing canonical transform (53). To see how this reference steady state is used to separate the two conjugate variables (base and tilt) in the symplectic transformations, it is helpful to recast Eq. (102) as

103 $$\begin{aligned} \frac{1}{2} \frac{ \left( {\phi }^{\dagger }_b {\phi }_b - {\phi }^{\dagger }_a {\phi }_a \right) }{ \left( {\phi }^{\dagger }_b {\phi }_b + {\phi }^{\dagger }_a {\phi }_a \right) }&\equiv \frac{ \left( {\nu }_b - {\nu }_a \right) }{ 2 } \equiv \nu \end{aligned}$$

The action of the tilt alone can be isolated, without regard to the underlying nominal distribution, by referencing the action of the ${\phi }^{\dagger }$ fields to the steady state rather than to $\phi$, defining an offset $\underline{\nu }$ as

104 $$\begin{aligned} \frac{1}{2} \frac{ \left( {\phi }^{\dagger }_b {\varvec{\nu }}_b - {\phi }^{\dagger }_a {\varvec{\nu }}_a \right) }{ \left( {\phi }^{\dagger }_b {\varvec{\nu }}_b + {\phi }^{\dagger }_a {\varvec{\nu }}_a \right) }&\equiv \frac{ \left( {\underline{\nu }}_b - {\underline{\nu }}_a \right) }{ 2 } \equiv \underline{\nu } \end{aligned}$$

Likewise, the mean value $\bar{\nu }$ of $\textrm{n}/ N$ in the base (nominal) distribution is isolated by referencing the value of $\phi$ to the uniform measure 1 instead of the dynamic measure ${\phi }^{\dagger }$, as

105 $$\begin{aligned} \frac{1}{2} \frac{ \left( {\phi }_b - {\phi }_a \right) }{ \left( {\phi }_b + {\phi }_a \right) }&\equiv \frac{ \left( {\bar{\nu }}_b - {\bar{\nu }}_a \right) }{ 2 } \equiv \bar{\nu } \end{aligned}$$

#### Stationary-path solutions and Liouville volume element

Solutions to the stationary-path equations of motion (47) for the Liouville function (97) are evaluated in Appendix F.1.

Stationary-path approximations to the time-dependent density $\rho$ would be binomial distributions even if the exact $\rho$ were not (the stationary point is always a pure coherent state), so the CGF at any time has the form (92), with fields *z* replaced by the stationary-path values of ${\phi }^{\dagger }$ and the mean values $\nu$ from Eq. (93) replaced by corresponding components of $\phi$.

In particular, the initial-time generating function ${\psi }_0 \left( \log {\phi }^{\dagger }_{a0} , \log {\phi }^{\dagger }_{b0} \right)$ appearing in Eq. (43) carries the mean value ${\bar{\nu }}_0$ in the starting density ${\rho }_{0}$, imposed as an initial-data parameter. It is through this function that the final-time tilt data in the form of the parameter ${\underline{\nu }}_T$, propagated backward to the stationary-path values of ${\phi }^{\dagger }_{a0}$ and ${\phi }^{\dagger }_{b0}$, determines the stationary path values for the fields $\phi$ of the base distribution, establishing the potential for information coupling between initial properties of the base distribution and final-time queries in the generating function ${\psi }_T$.

${\psi }_0$ is evaluated in Eq. (190), and the value is shown to depend only on an overlap parameter between initial and final data which we denote

106 $$\begin{aligned} \Lambda \equiv \frac{ \left( {\bar{\nu }}_0 - {\varvec{\nu }} \right) \left( {\underline{\nu }}_T - {\varvec{\nu }} \right) }{ \left( \frac{1}{4} - {\varvec{\nu }}^2 \right) } \end{aligned}$$

The stationary-path values of the displacement coordinates (104) and (105) are shown in Eq. (194) to follow simple exponential laws

107 $$\begin{aligned} \bar{\nu } - {\varvec{\nu }}&= \left( {\bar{\nu }}_0 - {\varvec{\nu }} \right) e^{-\tau }&\underline{\nu } - {\varvec{\nu }}&= \left( {\underline{\nu }}_T - {\varvec{\nu }} \right) e^{\tau - T} \end{aligned}$$

**Fig. 2:**
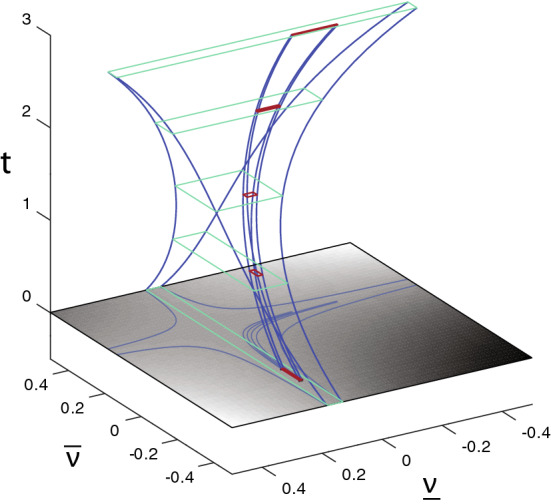
Four trajectories (heavy blue contours), plotted in coordinates $\left( \underline{\nu } , \bar{\nu } \right)$, that bound a region specified by ${\underline{\nu }}_T = -1/6 \pm 0.075$, ${\bar{\nu }}_0 = -1/4 \pm 0.05$. The steady state under the generating process sets ${\varvec{\nu }} = 1/6$. A time interval $T = 3$ between the input distribution and the final-time generating function is shown. Small rectangles (heavy dark red) show the area $\delta \underline{\nu } \, \delta \bar{\nu }$ at five equally spaced times from start to end. The outer four trajectories (thin blue) show the possible range of joint images of $-$ and . Large rectangles (thin green) show the constriction of the possible range $\propto e^{-T}$. Projections of the total range and the inner trajectories are shown in thin lines on the base plane. Shading of the base plane is a grayscale plot of ${\hat{\Phi }}_0^2$, which is constant along trajectories but variable over the $\left( \underline{\nu } , \bar{\nu } \right)$ coordinate range. Min and max of ${\hat{\Phi }}_0^2$ are respectively 0.83 and 1.1

Thus under independent variations of ${\bar{\nu }}_0$ and ${\underline{\nu }}_T$ as described in Sect. 3.4, the trajectories of the coherent state fields ${\phi }^{\dagger }$ and $\phi$ trace out an invariant volume, illustrated in Fig. 2.

#### Invariant cumulant-generating function and the incompressible phase-space density

The stationary value of ${\hat{\Phi }}_0$, obtained from the gradient of ${\psi }_0$ with respect to the components ${\phi }^{\dagger }_{0a}$ and ${\phi }^{\dagger }_{0b}$, is computed in Eq. (193). It differs from unity—the reason constructions (104) and (105) were needed—and it is equal to the stationary value of $\hat{\Phi }$ at all times as a consequence of conservation of total number *N*. The value depends only on $\Lambda$ and *T* in the combination

108 $$\begin{aligned} {\hat{\Phi }}_0 = \frac{ 1 }{ 1 + \Lambda e^{-T} } \end{aligned}$$

Moreover, as a consequence of the conserved Liouville volume element from Eq. (107), the stationary-point evaluation of the CGF at all times takes the same form as Eq. (92) and evaluates to the constant

109 $$\begin{aligned} \frac{\psi }{N}&= \log \left[ \frac{{\underline{\nu }}_a}{{\varvec{\nu }}_a} {\bar{\nu }}_a + \frac{{\underline{\nu }}_b}{{\varvec{\nu }}_b} {\bar{\nu }}_b \right] = - \log {\hat{\Phi }}_0 \end{aligned}$$

${\hat{\Phi }}_0$ in Eq. (108) is the basis for all information densities in this simple linear system. Through the stationary-point relation (61) between the Wigner function and the CGF, $-\log {\hat{\Phi }}_0$ is the incompressible phase-space density convected along stationary trajectories by Eq. (64). As shown below, it is also the geometrically invariant part of sole nonzero eigenvalue of the Fisher metric.

### Fisher metric

The Fisher metric (5) for the two-state system evaluates, along the stationary path at any time, to

110 $$\begin{aligned} \frac{g}{N}&= \frac{\partial \nu }{\partial \theta } \left[ \begin{array}{r} 1 \\ -1 \end{array} \right] \! \! \begin{array}{c} \left[ \begin{array}{cc} 1 &{} -1 \end{array} \right] \\ \end{array} \end{aligned}$$

The nonzero eigenvalue comes from the single-argument generating function in Eq. (96) for the difference coordinate $\textrm{n}$, and the zero eigenvalue comes from the linear CGF *hN* for the conserved quantity *N*.

The term $\partial \nu / \partial \theta$ in Eq. (110) may be converted, after some algebra, to the form

111 $$\begin{aligned} \frac{\partial \nu }{\partial \theta }&= \frac{ \left( \frac{1}{4} - {\underline{\nu }}^2 \right) \left( \frac{1}{4} - {\bar{\nu }}^2 \right) }{ \left( \frac{1}{4} - {\varvec{\nu }}^2 \right) } {\hat{\Phi }}_0^2 \end{aligned}$$

The measure terms  and  appearing in Eq. (111) follow the divisions (104) and (105) into independent dimensions of base and tilt variation, and we will show that their effects are canceled in an appropriate covariant derivative. The remaining dependence of the eigenvalue on the initial and final data is all carried in ${\hat{\Phi }}_0^2$.

### Dual coordinates for base and tilt, and the additive exponential family

To relate the Fisher metric in Eq. (110) to the construction of Sect. 3.3 from the $\psi$-divergence and to dually-symplectic parallel transport, we first express the base and tilt displacements (104) and (105) in terms of the coordinates in their respective exponential families.

Introduce reference values for the fields $\theta$ and *h* defined in Eq. (95), corresponding to the steady-state measure under the parameters of the generating process, denoted

112 $$\begin{aligned} {\varvec{\theta }}&\equiv \log \left( \frac{ \frac{1}{2} + {\varvec{\nu }} }{ \frac{1}{2} - {\varvec{\nu }} } \right) \nonumber \\ {\varvec{h}}&\equiv \frac{1}{2} \log \left( \frac{1}{4} - {\varvec{\nu }}^2 \right) = - \log \left[ 2 {{\,\textrm{ch}\,}}\frac{{\varvec{\theta }}}{2} \right] \end{aligned}$$

It is clear, in the two-argument generating function (92), that one component of variation in *z* couples only to the conserved quantity *N* and is not needed. It is sufficient therefore to vary along an affine coordinate in *z* that couples to the dynamical argument $\textrm{n}$, and the natural choice is to fix the component of *z* corresponding to the component of ${\phi }^{\dagger }$ that is invariant under the stationary-path equations of motion, given in Eq. (189). The resulting contour for *z* at final time *T* becomes

113 $$\begin{aligned} z_b {\varvec{\nu }}_b + z_a {\varvec{\nu }}_a&= {\Phi }^{\dagger }_T + {\varvec{\nu }} {\phi }^{\dagger }_T = 1&z_b - z_a&= {\phi }^{\dagger }_T = \frac{ {\underline{\nu }}_T - {\varvec{\nu }} }{ \frac{1}{4} - {\varvec{\nu }}^2 } \end{aligned}$$

The quantity in the first line of Eq. (113) is preserved at all times by Eq. (189), and the quantity in the second line obeys the exponential law of Eq. (194) repeated as (107).

By the definition (104), the contour (113) which is affine in coherent-state fields ${\phi }^{\dagger }$ is written in the coordinates on the exponential family of tilts as

114 $$\begin{aligned} \underline{\nu }&\equiv \frac{1}{2} {{\,\textrm{th}\,}}\left( \frac{ \theta + {\varvec{\theta }} }{ 2 } \right) \nonumber \\ 0&\equiv h + {\varvec{h}} + \log \left[ 2 {{\,\textrm{ch}\,}}\left( \frac{ \theta + {\varvec{\theta }} }{ 2 } \right) \right] \nonumber \\&= h - \frac{1}{2} \log \left( \frac{1}{4} - {\underline{\nu }}^2 \right) + \frac{1}{2} \log \left( \frac{1}{4} - {\varvec{\nu }}^2 \right) \end{aligned}$$

Likewise, in the dual exponential representation (55) of the family of base distributions, the definition (105) giving the mean number offset in the nominal distribution is expressed

115 $$\begin{aligned} \bar{\nu }&= \frac{1}{2} {{\,\textrm{th}\,}}\left( \frac{ \eta + {\varvec{\theta }} }{ 2 } \right) \end{aligned}$$

in which $\eta \equiv \left( {\eta }_b - {\eta }_a \right)$ in the dual number-potential system (55), analogously to $\theta$ in Eq. (95).

Because the two exponential coordinates (base and tilt) are additive, the mean of samples in the importance distribution can likewise be written

116 $$\begin{aligned} \nu&= \frac{1}{2} {{\,\textrm{th}\,}}\left( \frac{ \theta + \eta + {\varvec{\theta }} }{ 2 } \right) \end{aligned}$$

It follows that the eigenvalue (111) in the Fisher metric also has the simple expression

117 $$\begin{aligned} \frac{ \partial \nu }{ \partial \theta } = \frac{ \partial \nu }{ \partial \eta } = \frac{1}{4} - {\nu }^2 \end{aligned}$$

exhibiting the equivalence of the $\psi$-divergence expression (69) and the Hessian (71) for this quantity.

### Why coherent-state fields do not generally produce invertible coordinate transformations

The Hessian matrix is not a tensor under coordinate transform, so it is clear that the Hessian of $\psi$ with respect to the argument *z* equivalent to the coherent-state response field ${\phi }^{\dagger }$ will not be the Fisher metric. However, since coherent states are in many ways a native basis for Doi-Peliti theory, as noted in Sect. 3.6, we may ask whether some other coordinate duality can be defined from the coherent-state Hessian of $\psi$. In fact such a duality cannot generally be defined, and it is instructive to see where it fails, to better understand why the affine connection (90) and not the Fisher geometry captures the special role of coherent states.

A divergence under the Hessian of $\psi$ in coherent-state variables, which we will denote $\Delta \delta s^2$ for reasons to become clear in a moment, if converted from the coordinates $\underline{\nu }$ to coordinates $\theta$ along the *z*-affine contour (114), evaluates as

118 $$\begin{aligned} \frac{1}{N} \Delta ds^2&\equiv { \left( \delta \theta \right) }^2 { \left( \frac{1}{4} - {\underline{\nu }}^2 \right) }^2 \frac{ {\partial }^2 }{ \partial {\underline{\nu }}^2 } \left( \frac{ \psi }{ N } \right) \equiv - { \left( \delta \theta \right) }^2 { \left( \nu - \underline{\nu } \right) }^2 \end{aligned}$$

Unlike the Fisher metric, Eq. (118) is negative-semidefinite, and degenerates if $\nu = \underline{\nu }$, which is shown in Eq. (197) to hold for all *z* if ${\bar{\nu }}_0 = {\varvec{\nu }}$. At degenerate solutions, we cannot use the Hessian of $\psi$ to define a base-field variation $\delta \phi$ as a dual coordinate for a variation produced by a field $\delta {\phi }^{\dagger }$, as we *could* use the Hessian in the exponential family to produce a variation $\delta n$ as a dual coordinate to a variation $\delta \theta$.

The source of the degeneration has a nice description in terms of intrinsic and extrinsic curvatures, and advection, in the natural geometry on the exponential family. The geometric distance element (4), with $\theta$ and *h* varied independently, is

119 $$\begin{aligned} \frac{1}{N} \delta s^2&= {\left( \delta \theta \right) }^2 \frac{{\partial }^2}{\partial {\theta }^2} \left( \frac{\psi }{N} - h \right) + {\left( \delta h \right) }^2 \frac{{\partial }^2}{\partial h^2} h \nonumber \\&= {\left( \delta \theta \right) }^2 \frac{\partial \nu }{\partial \theta } + {\left( \delta h \right) }^2 0 \end{aligned}$$

The *z*-affine contour (114) specifies a function $h \! \left( \theta \right)$ with extrinsic curvature in the affine coordinate manifold of the exponential family, along which the distance element is

120 $$\begin{aligned} \frac{1}{N} \delta s^2_\mathrm{CS-ext}&= {\left( \delta \underline{\nu } \right) }^2 \frac{ d^2 }{ d {\underline{\nu }}^2 } \left( -h \! \left( \theta \right) \right) = {\left( \delta \theta \right) }^2 \left( \frac{1}{4} + {\underline{\nu }}^2 \right) \end{aligned}$$

The second coherent-state coordinate derivative of $\psi$ along the contour (114) can be decomposed as

121 $$\begin{aligned} \frac{1}{N} \Delta \delta s^2&= {\left( \delta \underline{\nu } \right) }^2 \frac{ d^2 }{ d {\underline{\nu }}^2 } \left( \frac{ \psi }{ N } \right) \nonumber \\&= {\left( \delta \underline{\nu } \right) }^2 \left[ \frac{ d^2 }{ d {\underline{\nu }}^2 } \left( \frac{ \psi }{ N } - h \right) + \frac{ d^2 }{ d {\underline{\nu }}^2 } h \right] \nonumber \\&= {\left( \delta \theta \right) }^2 \left( \frac{ d^2 }{ d {\theta }^2 } + 2 \underline{\nu } \frac{ d }{ d \theta } \right) \left( \frac{ \psi }{ N } - h \right) + {\left( \delta \underline{\nu } \right) }^2 \frac{ d^2 }{ d {\underline{\nu }}^2 } h \nonumber \\&= {\left( \delta \theta \right) }^2 \left[ \frac{\partial \nu }{\partial \theta } + 2 \underline{\nu } \nu \right] + {\left( \delta \underline{\nu } \right) }^2 \frac{ d^2 }{ d {\underline{\nu }}^2 } h \nonumber \\&= \frac{1}{N} \left( \delta s^2 - \delta s^2_\mathrm{CS-ext} \right) + {\left( \delta \theta \right) }^2 2 \underline{\nu } \nu \end{aligned}$$

With some algebra, the expression (121) is shown to equal that in Eq. (118). The extrinsic curvature of the embedded contour $h \! \left( \theta \right)$ and the convected quantity $- 2 \underline{\nu } \nu$ cancel against the intrinsic Fisher-Rao curvature, rendering the duality invisible to the fields ${\phi }^{\dagger }$ at degenerate points.

### Flat transport in the coherent-state connection

The correct way to capture the simplifying role of coherent-state coordinates for simple models such as the two-state system is with the dual connections of Sect. 3.5.

We first recognize, from the forms (106) or (195) of $\Lambda$, a completely-descaled coordinate system for the dynamical parts of the coherent-state fields, defined by

122 $$\begin{aligned} v&\equiv \frac{ \left( \underline{\nu } - {\varvec{\nu }} \right) }{ \sqrt{ \frac{1}{4} - {\varvec{\nu }}^2 } }&u&\equiv \frac{ \left( \bar{\nu } - {\varvec{\nu }} \right) }{ \sqrt{ \frac{1}{4} - {\varvec{\nu }}^2 } } \end{aligned}$$

The eigenvalue of the Fisher metric in Eq. (111) then reduces to

123 $$\begin{aligned} \frac{ {\partial }^2 }{ \partial {\theta }^2 } \! \left( \frac{{\psi }}{N} \right) = \frac{ \left( dv / d\theta \right) \left( du / d\eta \right) }{ { \left[ 1 + u v \right] }^2 } \end{aligned}$$

The role of the factors  and  in Eq. (111) as measure terms is now explicit, and they can be absorbed by a change of variables to *u* and *v*. By Eq. (195) and the definitions (122) and (108), $\left[ 1 + u v \right] = \left[ 1 + \Lambda e^{-T} \right] = 1/{\hat{\Phi }}_0$, so the Fisher inner product (12) may be written

124 $$\begin{aligned} \delta \theta \, {\delta }_{\eta } n \left\langle \frac{\partial }{\partial \theta } , \frac{\partial }{\partial n} \right\rangle = \delta \underline{\nu } \, \delta \bar{\nu } \left\langle \frac{\partial }{\partial \underline{\nu }} , \frac{\partial }{\partial \bar{\nu }} \right\rangle&= \delta u \, \delta v \, {\hat{\Phi }}_0^2 \end{aligned}$$

#### Connection coefficients and absorption of measure terms

In this linear model, time evolution of ${\phi }^{\dagger }$ and $\phi$ has no cross-dependence once the initial values have been fixed through the gradients of ${\psi }_0$ as explained in Sect. 4.2.2. Thus ${ \left( {\nabla }^{\left( \eta \right) }_k \delta \theta \right) }^j = 0$ and ${\left( {\nabla }^{\left( \theta \right) *}_k \delta \eta \right) }^j = 0$.

Appendix F.4 computes connection coefficients and covariant derivatives for the vector fields corresponding to Eq. (84), and for the metric tensor corresponding to Eq. (85). Eq. (205) in the appendix gives the covariant part of the time derivatives of $\delta \theta$ and $\delta \eta$ as

125 $$\begin{aligned} \left( \frac{\partial }{\partial \tau } \delta \theta \right) + \dot{\theta } \left( {\nabla }_{\theta } \delta \theta \right)&= \delta \theta&\left( \frac{\partial }{\partial t} \delta \eta \right) + \dot{\eta } \left( {\nabla }^{*}_{\eta } \delta \eta \right)&= - \delta \eta \end{aligned}$$

capturing the simple exponential scaling (107) of the coherent-state fields in the exponential-family coordinates.

The covariant part of the change in the Fisher metric, from Eq. (85) is computed in Eq. (207) to be

126 $$\begin{aligned} \dot{\theta } {\nabla }_{\theta } g&= \left( \dot{v} \frac{\partial }{\partial v} \log {\hat{\Phi }}_0^2 \right) g&\dot{\eta } {\nabla }^{*}_{\eta } g&= \left( \dot{u} \frac{\partial }{\partial u} \log {\hat{\Phi }}_0^2 \right) g \end{aligned}$$

Only the dependence in the Fisher eigenvalue ${\hat{\Phi }}_0^2$ from Eq. (124) appears.

The two lines of Eq. (126) (which are equal and opposite) scale as $\sim e^{-T}$, and have an interpretation similar to that of a Le Chatelier principle. The term $\Lambda e^{-T}$ in Eq. (108) for ${\hat{\Phi }}_0$ is a susceptibility of the initial stationary value ${\phi }_0$ to the perturbation by the tilt variable ${\phi }^{\dagger }_T = z$, attenuated exponentially from time *T* to time 0. The role of this attenuation, which takes ${\hat{\Phi }}_0 \rightarrow 1$ as $T \rightarrow \infty$, becomes clearer as a constraint on the total extractable information when we consider in Sect. 4.7 the range of all initial distributions ${\rho }_0$ and all tilts *z*.

#### Duality of dynamics and inference in Doi-Peliti theory

The natural separation of the coordinate transformation of the inner product of vector fields $\delta \theta$ and $\delta \eta$ generated by time translation is not between exponential and mixture coordinates, as in the dually-flat connections of Amari [2], but rather between the symplectically dual contributions from changes in $\theta$ and in $\eta$. The two contributions group as

127 $$\begin{aligned} 0&= \frac{d}{dt} \left( \delta \theta \, g \, \delta \eta \right) \nonumber \\&= \left( \frac{\partial }{\partial t} \delta \theta \right) {\delta }_{\eta } \nu + \dot{\theta } {\nabla }^{\left( \theta \right) }_{\theta } \! \left( \delta \theta \, g \right) \delta \eta + {\delta }_{\theta } \nu \left( \frac{\partial }{\partial t} \delta \eta \right) + \delta \theta \, \dot{\eta } {\nabla }^{\left( \eta \right) *}_{\eta } \! \left( g \, \delta \eta \right) \end{aligned}$$

The two rows of Eq. (127) add covariant contributions from Eq. (125) and Eq. (126) in the combinations

128 $$\begin{aligned} \left( \frac{\partial }{\partial t} \delta \theta \right) {\delta }_{\eta } \nu + \dot{\theta } {\nabla }^{\left( \theta \right) }_{\theta } \! \left( \delta \theta \, g \right) \delta \eta&= \left( \delta \theta \, {\delta }_{\eta } \nu \right) \left( 1 + \dot{v} \frac{\partial }{\partial v} \log {\hat{\Phi }}_0^2 \right) \nonumber \\ {\delta }_{\theta } \nu \left( \frac{\partial }{\partial t} \delta \eta \right) + \delta \theta \, \dot{\eta } {\nabla }^{\left( \eta \right) *}_{\eta } \! \left( g \, \delta \eta \right)&= \left( {\delta }_{\theta } \nu \, \delta \eta \right) \left( - 1 + \dot{u} \frac{\partial }{\partial u} \log {\hat{\Phi }}_0^2 \right) \end{aligned}$$

Eq. (128) captures in the clearest way possible the symplectic balance of distribution dynamics (through $\eta$) and inference (through $\theta$) in Doi-Peliti theory, through both the direct effects of the exponential growth and decay eigenvalues $\left( \pm 1 \right)$ and the Le Chatelier-like susceptibility of the density ${\hat{\Phi }}_0$.

### The Fisher information density and large-deviation ratios as sample estimators

The interpretation of the vector inner product as a convected density of information can be illustrated by using ratios of large-deviation probabilities to define a sample estimator for differences in the tilt coordinate $\eta$ between two base distributions.

Suppose that we sample from a binomial nominal distribution at a parameter $\eta$ that is to be estimated. Recall from Eq. (24) that the probability for the value $\textrm{n}$ of a sample to exceed a threshold *n* is given in terms of the large-deviation function by

129 $$\begin{aligned} P \! \left( \textrm{n}\ge n \mid \eta \right) \sim e^{ - {\psi }^{*} \left( n ; \eta \right) } \end{aligned}$$

In a 1-dimensional system,^6^ for two threshold values $n_B > n_A$, the conditional probability for $\textrm{n}$ to surpass $n_B$ given that it has surpassed $n_A$ is the ratio

130 $$\begin{aligned} P \! \left( n_B \mid n_A ; \eta \right)&\equiv \frac{ P \! \left( \textrm{n}\ge n_B \mid \eta \right) }{ P \! \left( \textrm{n}\ge n_A \mid \eta \right) } \sim e^{ - \left[ {\psi }^{*} \left( n_B ; \eta \right) - {\psi }^{*} \left( n_A ; \eta \right) \right] } \end{aligned}$$

The ratio (130) can be estimated from samples of the indicator function $h_{\textrm{n}}$ for thresholds *n* as described in Sect. 2.2.

Appendix F.5 shows that if two such conditional probabilities are compared from distributions at unknown parameters ${\eta }_2$ and ${\eta }_1$, the log ratio is related to the large-deviation thresholds and the $\eta$ values as

131 $$\begin{aligned} \log \left( \frac{ P \! \left( n_B \mid n_A ; {\eta }_2 \right) }{ P \! \left( n_B \mid n_A ; {\eta }_1 \right) } \right)&\sim \int _{{\eta }_1}^{{\eta }_2} \! \! \int _{n_A}^{n_B} d_{\theta } n \, d\eta = \left( n_B - n_A \right) \left( {\eta }_2 - {\eta }_1 \right) \end{aligned}$$

where $d_{\theta } n \, d\eta$ is one of the two forms of the (differential) inner product appearing in Eq (76).

Thus

132 $$\begin{aligned} \frac{ \log \left( \frac{ P \! \left( n_B \mid n_A ; {\eta }_2 \right) }{ P \! \left( n_B \mid n_A ; {\eta }_1 \right) } \right) }{ \left( n_B - n_A \right) } \sim \left( {\eta }_2 - {\eta }_1 \right) \end{aligned}$$

is a sample estimator for the difference of exponential parameters in the two underlying distributions.

The quantity (131) may be computed at any time, for instance the final time *T* when the thresholds $n_B$ and $n_A$ are imposed as experimental conditions, and ${\eta }_2$ and ${\eta }_1$ characterize evolved nominal distributions at time *T* from any pair of initial conditions at some earlier time $t = 0$. If we use the stationary-path conditions to propagate values of $\theta$ and $\eta$ through time, and define $V \! \left( \tau \right)$ to be the area inside the image of the rectangle in Eq. (131) along these stationary trajectories, time-invariance of the inner product, and the Liouville conservation of volume elements in dual coordinates, implies that

133 $$\begin{aligned} \frac{d}{d\tau } \int _{V} d_{\theta } n \, d\eta = 0 \end{aligned}$$

Note that, with a coordinate transform to coherent-state variables and a corresponding redefinition of the boundary of *V*, the relation (133) could be recast using Eq. (124) as

134 $$\begin{aligned} \frac{d}{d\tau } \int _{V} dv \, du \, {\hat{\Phi }}_0^2 = 0 \end{aligned}$$

which is the conserved integral graphed in Fig. 2.

In Eq. (134) ${\hat{\Phi }}_0^2$, the 2-dimensional differential of the scaled CGF $\psi / N = - \log {\hat{\Phi }}_0$, appears explicitly as the density of overlap of *dv* with *du* that, like $\psi$ itself, is constant along stationary paths. ${\hat{\Phi }}_0^2$ is not independent of the position $\left( v , u \right)$ within the volume *V*, but because the volume element moves along with the conserved density, the integral measures a fixed quantity of Fisher information as it is transported through different domains of base and tilt.

Although the limits of integration for $\int dv \, du$ in Eq. (134) are bounded, the limits on $\left( {\eta }_2 - {\eta }_1 \right)$ in Eq. (131) are not, so formally the range of the sample estimator (132) remains unbounded over any duration *T*. However, for any *fixed* values of ${\left( n_B - n_A \right) }_{t = T}$ and starting uncertainty ${\left( {\eta }_2 - {\eta }_1 \right) }_{t = 0}$, the total information obtainable from large-deviations sampling about differences in the initial conditions is finite and decreases as $e^{-T}$. In Fig. 2 this limit is seen in the way any fixed ranges are squeezed exponentially at the “waist” as $T \rightarrow \infty$. The contraction of boundaries, rather than the asymptotic behavior of the eigenvalue in the Fisher metric, measures the loss of information between initial distributions and final observations with increasing separation between the two.

## Conclusions: the duality of dynamics and inference for irreversible and reversible processes

The three-part structure of the Fisher metric, dual Riemannian connections, and symplectic parallel transport of the Wigner density, vector fields, and the metric tensor, elegantly expresses the transport properties along 2FFI stationary paths in terms of geometric invariants. It resolves a feature of two-field constructions that at first seems paradoxical: if memory of initial conditions is continuously lost to dissipation, what concept of time-reversal is implied by invertibility of the map along stationary rays? The answer from the perspective of importance sampling is that, even if samples are finite, their expectations are computed in continuous-valued distributions, and deformations of measure through the Radon–Nikodym derivative can locally compensate for concentration of measure in the nominal distribution by expanding sensitivity of likelihood ratios. Locally in sampling space, then, time is immaterial as it is in Hamiltonian mechanics; the mappings along stationary trajectories make it possible to interpret sampling protocols from different times in an evolving distribution simply as coordinate transformations of a fixed sampling protocol on the original distribution. On the other hand, for any *fixed* ranges of parameter variation in the initial conditions, and fixed large-deviation thresholds compared at late time, the integrated Liouville density contracts monotonically with the separation between the two times, reflecting the absolute loss of information that can be recovered.

We have wanted to establish a concrete interpretation of time-duality in 2FFI theories as a duality of dynamics and inference, to provide an alternative to the interpretation in terms of *physical* reversal of paths that is the starting point in most of the literature on fluctuation theorems in stochastic thermodynamics. Microscopic reversibility can always be added later to any class of 2FFI constructions as a restriction on the scope of phenomena under study, and both stronger conclusions and additional interpretations will then follow from the added constraints. Where the existence of a duality in the mathematics itself does not depend on any such additional assumptions, taking the inference interpretation to reflect the core concepts, directly expressing Kolmogorov’s forward/backward adjoint duality, frames the special case of microscopic reversibility as one in which the system’s own dynamics contains an image of certain sampling protocols over itself.

Even if one only cares about microscopically reversible processes, making explicit the step of self-modeling, and having a concrete interpretation of conserved densities such as the Fisher information constructed here, provides a bridge between trajectory reversal in low-level mechanics and operations for sample estimation of the kind that are used by control systems. Linking limitations from path probability in a system’s autonomous dynamics to concepts of information capacity in control loops [5, 6, 18] promises a way to study the limits on spontaneous emergence of dynamical hierarchy, which has been a desired application for stochastic thermodynamics [23, 59]. These are intended topics for future work.

## Fisher spherical embeddings

### The embedding for general distributions on finite state spaces

Equation (4) in the text can be written in the form

135 $$\begin{aligned} \delta s^2 = 4 \delta {\theta }^i \delta {\theta }^j \sum _{\textrm{n}} \frac{ \partial \sqrt{{\tilde{\rho }}_{\textrm{n}}^{\left( \theta \right) }} }{ \partial {\theta }^i } \frac{ \partial \sqrt{{\tilde{\rho }}_{\textrm{n}}^{\left( \theta \right) }} }{ \partial {\theta }^j } \end{aligned}$$

Let $\left| \left\{ \textrm{n}\right\} \right|$ be the cardinality of the set of states on which ${\rho }_{\textrm{n}}$ is defined (for example, in chemistry, only a sub-lattice of all integer-valued vectors in the positive orthant may ever be accessible as counts, given a system’s stoichiometry and conserved quantities). Suppose $\left| \left\{ \textrm{n}\right\} \right|$ is finite in order to illustrate the Fisher embedding geometry for distributions over finite state spaces. All possible base distributions $\rho$ fall within the simplex of dimension $\left| \left\{ \textrm{n}\right\} \right| - 1$.

Now let $\left\{ {\alpha }_1, {\alpha }_2, \ldots {\alpha }_{\left| \left\{ \textrm{n}\right\} \right| - 1} \right\}$ be angles associated with independent rotation axes in ${\mathbb {R}}^{\left| \left\{ \textrm{n}\right\} \right| }$. Any distribution can be embedded in ${\mathbb {R}}^{\left| \left\{ \textrm{n}\right\} \right| }$ by arranging the states $\textrm{n}$ in an (arbitrary) order ${\textrm{n}}_1 , {\textrm{n}}_2 , \ldots {\textrm{n}}_{\left| \left\{ \textrm{n}\right\} \right| }$, and writing

136 $$\begin{aligned} p_{{\textrm{n}}_1}&\equiv \cos ^2 {\alpha }_1 \nonumber \\ p_{{\textrm{n}}_2}&\equiv \sin ^2 {\alpha }_1 \cos ^2 {\alpha }_2 \nonumber \\ p_{{\textrm{n}}_3}&\equiv \sin ^2 {\alpha }_1 \sin ^2 {\alpha }_2 \cos ^2 {\alpha }_3 \nonumber \\&\, \, \vdots \nonumber \\ p_{{\textrm{n}}_{\left| \left\{ \textrm{n}\right\} \right| - 1}}&\equiv \sin ^2 {\alpha }_1 \sin ^2 {\alpha }_2 \cdots \sin ^2 {\alpha }_{\left| \left\{ \textrm{n}\right\} \right| - 2} \cos ^2 {\alpha }_{\left| \left\{ \textrm{n}\right\} \right| - 1} \nonumber \\ p_{{\textrm{n}}_{\left| \left\{ \textrm{n}\right\} \right| }}&\equiv \sin ^2 {\alpha }_1 \sin ^2 {\alpha }_2 \cdots \sin ^2 {\alpha }_{\left| \left\{ \textrm{n}\right\} \right| - 2} \sin ^2 {\alpha }_{\left| \left\{ \textrm{n}\right\} \right| - 1} \end{aligned}$$

A recursive calculation gives the line element (135) in terms of the angle coordinates on the radius-2 sphere as

137 $$\begin{aligned} \delta s^2&= 4 \left[ \delta {\alpha }_1^2 + \sin ^2 {\alpha }_1 \, \delta {\alpha }_2^2 + \cdots + \left( \sin ^2 {\alpha }_1 \ldots \sin ^2 {\alpha }_{\left| \left\{ \textrm{n}\right\} \right| - 2} \right) \delta {\alpha }_{\left| \left\{ \textrm{n}\right\} \right| - 1}^2 \right] \end{aligned}$$

### Embeddings in reduced dimension for exponential families on the multinomial

The Poisson (35) and multinomial (36) distributions are both in a class recognized by Anderson, Craciun, and Kurtz (ACK) [4] in connection with uniqueness of stationary solutions for chemical reaction networks. All factorial moments are powers of their first moments, causing the CGF for many particles to scale as a multiple of a single-particle CGF. It is not then surprising that the expression (4) for the Fisher metric in terms of a distribution ${\tilde{\rho }}_{\textrm{n}}$ with possibly indefinitely many independent terms, for the ACK distributions projects to a function of the same form in terms of expected numbers $n_i$ over the *D* independent species.

To see how this works for a distribution ${\rho }^{\left( n_0 \right) }_{\textrm{n}}$ with multinomial form (36), express the expected number fractions as

138 $$\begin{aligned} \frac{n_{0i}}{N} = \frac{ e^{{\eta }_i} }{ \sum _{j=1}^D e^{{\eta }_j} } \end{aligned}$$

Then the mean in the distribution ${\tilde{\rho }}_{\textrm{n}}^{\left( \theta ; n_0 \right) }$ is

139 $$\begin{aligned} \frac{n_i \! \left( \theta \right) }{N} = \frac{ e^{{\eta }_i + {\theta }_i} }{ \sum _{j=1}^D e^{{\eta }_j + {\theta }_j} } \equiv {\nu }_i \end{aligned}$$

and the CGF $\psi \! \left( \theta ; n_0 \right)$ evaluates (up to a constant offset) to

140 $$\begin{aligned} \psi \! \left( \theta ; n_0 \right) = N \log \left[ \sum _{j = 1}^D e^{{\eta }_j + {\theta }_j} \right] \end{aligned}$$

The Hessian giving the Fisher metric is

141 $$\begin{aligned} g_{ij} \! \left( \theta \right) = N \left\{ {\nu }_i {\delta }_{ij} - {\nu }_i {\nu }_j \right\} \end{aligned}$$

where ${\nu }_i$ is the function of $\eta + \theta$ in Eq. (139). Inverting Eq. (141), and projecting onto the $\sum _i {\theta }_i = 0$ plane to fix the undetermined component of $\theta$, gives the inverse

142 $$\begin{aligned} g^{ij} \! \left( n \right) = \frac{1}{N} \left\{ \frac{1}{{\nu }_i} {\delta }_{ij} - \frac{1}{D} \left[ \frac{1}{{\nu }_i} + \frac{1}{{\nu }_j} - \frac{1}{D} \sum _k \frac{1}{{\nu }_k} \right] \right\} \end{aligned}$$

One can check that both $g \! \left( \theta \right)$ and $g^{-1} \! \left( n \right)$ sum to zero on either index, and the product

143 $$\begin{aligned} g^{ij} \! \left( n \right) g_{jk} \! \left( \theta \right) = {\delta }^i_k - \frac{1}{D} \end{aligned}$$

is the identity on the subspace $\sum _{j=1}^D {\theta }^j = 0$ or $\sum _{j=1}^D n_j = N$.

If a shift of the tilted distribution in the exponential family is indexed with coordinate $\delta n$, with $\sum _{j=1}^D \delta n_j = 0$, the Fisher distance element from Eq. (11) becomes

144 $$\begin{aligned} \delta s^2 = \sum _{j=1}^D \frac{\delta n_j^2}{n_j} \end{aligned}$$

which is the same function of *n* as the function of $\tilde{\rho }$ in the third line of Eq. (4).

## Sample means and variances in the large-deviation approximation to threshold indicator expectations

The expectation of the tilted indicator function from Eq. (23) may be written in a series of inequalities culminating in the expression for the CGF, as

145 $$\begin{aligned} {\left\langle {\tilde{h}}^{\theta } \right\rangle }_{\left( \theta ; n_0 \right) }&= \sum _{\textrm{n}> \bar{n}} e^{ - \theta \textrm{n}} e^{ \theta \textrm{n}} {\rho }^{\left( n_0 \right) }_{\textrm{n}} \nonumber \\&\le e^{ - \theta \bar{n} } \sum _{\textrm{n}> \bar{n}} e^{ \theta \textrm{n}} {\rho }^{\left( n_0 \right) }_{\textrm{n}} \nonumber \\&\le e^{ - \theta \bar{n} } \sum _{\textrm{n}} e^{ \theta \textrm{n}} {\rho }^{\left( n_0 \right) }_{\textrm{n}} \nonumber \\&= e^{ \psi \left( \theta ; n_0 \right) - \theta \bar{n} } \end{aligned}$$

providing Eq. (24) in the text.

The variance of the same sample estimator has a corresponding bound

146 $$\begin{aligned} { \left\langle { \left( {\tilde{h}}^{\theta } \right) }^2 \right\rangle }_{\left( \theta ; n_0 \right) } - {\left\langle {\tilde{h}}^{\theta } \right\rangle }_{\left( \theta ; n_0 \right) }^2&= { \left\langle { \left( {\tilde{h}}^{\theta } \right) }^2 \right\rangle }_{\left( \theta ; n_0 \right) } - {\left\langle h \right\rangle }_{\left( n_0 \right) }^2 \nonumber \\&= { \left\langle e^{ \psi \left( \theta ; n_0 \right) - \theta \textrm{n}} {\tilde{h}}^{\theta } \right\rangle }_{\left( \theta ; n_0 \right) } - {\left\langle h \right\rangle }_{\left( n_0 \right) }^2 \nonumber \\&\le e^{ \psi \left( \theta ; n_0 \right) - \theta \bar{n} } { \left\langle {\tilde{h}}^{\theta } \right\rangle }_{\left( \theta ; n_0 \right) } - {\left\langle h \right\rangle }_{\left( n_0 \right) }^2 \nonumber \\&= e^{ \psi \left( \theta ; n_0 \right) - \theta \bar{n} } {\left\langle h \right\rangle }_{\left( n_0 \right) } - {\left\langle h \right\rangle }_{\left( n_0 \right) }^2 \end{aligned}$$

giving Eq. (25) in the text.

To estimate the tightness of the bounds, begin by observing that in the large-deviation scaling regime (27), with all cumulants generated as derivatives of $\psi \sim N$, the expansion of central moments in terms of cumulants bounds the scaling of the *k*th central moment as

147 $$\begin{aligned} \left\langle { \left( \textrm{n}- \left\langle \textrm{n}\right\rangle \right) }^k \right\rangle / {\left\langle \textrm{n}\right\rangle }^k \le \mathcal {O} \! \left( N^{ - \left\lceil k/2 \right\rceil } \right) \end{aligned}$$

The log ratio we wish to bound is the Bregman divergence

148 $$\begin{aligned} \log \left[ \frac{ \sum _{\textrm{n}> \bar{n}} e^{- \theta \left( \textrm{n}- \bar{n} \right) } e^{\theta \textrm{n}} {\rho }_{\textrm{n}} }{ \sum _{\textrm{n}} e^{\theta \textrm{n}} {\rho }_{\textrm{n}} } \right] = \theta \bar{n} - \psi \! \left( \theta , n_0 \right) + \log {\left\langle h^{\left( \bar{n} \right) } \right\rangle }_{\left( n_0 \right) } \end{aligned}$$

The maximum of Eq. (148) occurs at $\theta \! \left( \bar{n} \right)$ from Eq. (8), and the width of the transition for the log ratio to change by $\mathcal {O} \! \left( N^0 \right)$ is given by

149 $$\begin{aligned} \delta \theta \approx { \left( { \left. \frac{{\partial }^2 \psi }{{\partial \theta }^2} \right| }_{ \theta \left( \bar{n} \right) } \right) }^{-1} \sim N^{-1/2} \end{aligned}$$

To estimate its maximum value we write Eq. (148) as the sum of log ratios of the two inequalities in Eq. (145), and observe that they have boundary values

150 $$\begin{aligned} { \left. \log \left[ \frac{ \sum _{\textrm{n}> \bar{n}} e^{- \theta \left( \textrm{n}- \bar{n} \right) } e^{\theta \textrm{n}} {\rho }_{\textrm{n}} }{ \sum _{\textrm{n}> \bar{n}} e^{\theta \textrm{n}} {\rho }_{\textrm{n}} } \right] \right| }_{ \theta = 0 }&= 0&{ \left. \log \left[ \frac{ \sum _{\textrm{n}> \bar{n}} e^{\theta \textrm{n}} {\rho }_{\textrm{n}} }{ \sum _{\textrm{n}} e^{\theta \textrm{n}} {\rho }_{\textrm{n}} } \right] \right| }_{ \theta \rightarrow \infty }&= 0 \end{aligned}$$

The values of these ratios at intermediate $\theta$ are then obtained by integrating the derivatives

151 $$\begin{aligned} \frac{\partial }{\partial \theta } \log \left[ \frac{ \sum _{\textrm{n}> \bar{n}} e^{- \theta \left( \textrm{n}- \bar{n} \right) } e^{\theta \textrm{n}} {\rho }_{\textrm{n}} }{ \sum _{\textrm{n}> \bar{n}} e^{\theta \textrm{n}} {\rho }_{\textrm{n}} } \right]&= - \frac{ \sum _{\textrm{n}> \bar{n}} \left( \textrm{n}- \bar{n} \right) e^{\theta \textrm{n}} {\rho }_{\textrm{n}} }{ \sum _{\textrm{n}> \bar{n}} e^{\theta \textrm{n}} {\rho }_{\textrm{n}} } \nonumber \\ \frac{\partial }{\partial \theta } \log \left[ \frac{ \sum _{\textrm{n}> \bar{n}} e^{\theta \textrm{n}} {\rho }_{\textrm{n}} }{ \sum _{\textrm{n}} e^{\theta \textrm{n}} {\rho }_{\textrm{n}} } \right]&= \frac{ \sum _{\textrm{n}> \bar{n}} \left( \textrm{n}- n \! \left( \theta \right) \right) e^{\theta \textrm{n}} {\rho }_{\textrm{n}} }{ \sum _{\textrm{n}> \bar{n}} e^{\theta \textrm{n}} {\rho }_{\textrm{n}} } \end{aligned}$$

Both log derivatives in Eq. (151) are monotone if $\psi$ is convex, and their values sum to $\bar{n} - n \! \left( \theta \right)$, the derivative of Eq (148), where $n \! \left( \theta \right) \equiv {\left\langle \textrm{n}\right\rangle }_{{\tilde{\rho }}^{\left( \theta , n_0 \right) }}$.

Next, observe the leading-order scaling of the expectation ${\left\langle {\left( \textrm{n}- \bar{n} \right) }^2 \right\rangle }_{\textrm{n}> \bar{n}} \approx {\left\langle \left( \textrm{n}- \bar{n} \right) \right\rangle }^2_{\textrm{n}> \bar{n}}$ on the half-line (as for any second moment), and likewise for $\left( \textrm{n}- n \! \left( \theta \right) \right)$.

At $\theta \! \left( \bar{n} \right)$, where $n \! \left( \theta \right) = \bar{n}$, the two derivatives (151) are equal and opposite, and the boundary of the half-line $\textrm{n}> \bar{n}$ is also the symmetry point of ${\left( \textrm{n}- \bar{n} \right) }^2$. Because the skewness and higher-order central moments grow more slowly than ${\bar{n}}^k$ by Eq. (147), ${\left\langle {\left( \textrm{n}- \bar{n} \right) }^2 \right\rangle }_{\textrm{n}> \bar{n}} \approx {\left\langle {\left( \textrm{n}- \bar{n} \right) }^2 \right\rangle }_{\textrm{n}} \times \left\{ 1 + \mathcal {O} \! \left( 1 / N \right) \right\}$, and given the large-deviations scaling of central moments (147), the derivatives in Eq. (151) scale as

152 $$\begin{aligned} { \left. \frac{ \sum _{\textrm{n}> \bar{n}} \left( \textrm{n}- \bar{n} \right) e^{\theta \textrm{n}} {\rho }_{\textrm{n}} }{ \sum _{\textrm{n}> \bar{n}} e^{\theta \textrm{n}} {\rho }_{\textrm{n}} } \right| }_{ n \left( \theta \right) = \bar{n} } \sim \sqrt{ \left\langle { \left( \textrm{n}- \bar{n} \right) }^2 \right\rangle } \sim N^{1/2} \end{aligned}$$

Over the range $\pm \delta \theta$ from Eq. (149), where the total log ratio changes by $\mathcal {O} \! \left( N^0 \right)$, the integral of the first derivative (152) saturates the lower limit in the first line of Eq. (150), and the upper limit in the second line of Eq. (150), to within $\le \mathcal {O} \! \left( N^{1/2} \right)$, implying that the log-ratios themselves at the midpoint scale as $\le \mathcal {O} \! \left( N^{1/2} \right)$. Hence also the total log ratio that is their sum scales as $\log \left[ {\left\langle h^{\left( \bar{n} \right) } \right\rangle }_{\left( n_0 \right) } / e^{- {\psi }^{*} \left( \bar{n} \right) } \right] \le \mathcal {O} \! \left( N^{1/2} \right)$, the result used in the text.

## Review of Doi Hilbert space and Peliti functional integral constructions

### Doi operator algebra and inner product

The main constructs in the Doi operator formulation [21, 22] of moment-generating functions as formal power series are as follows:

The identification (30) of *z* and $\partial / \partial z$ with raising and lowering operators $a^{\dagger }$ and *a* allows the commutator algebra

153 $$\begin{aligned} \left[ a_i , a^{\dagger }_j \right] = {\delta }_{ij} . \end{aligned}$$

to stand for the commutator algebra between components of $\partial / \partial z$ and factors of *z*, applied by function composition acting to the right on MGFs.

Monomials $z^{\textrm{n}}$ in Eq. (1) are basis elements in a linear space of MGFs, built up by multiplication on the number 1. A bracket notation for states and an inner product are introduced by the pair of denotations

154 $$\begin{aligned} 1&\rightarrow \left| 0 \right)&\int d^D \! z \, {\delta }^D \! \left( z \right)&\rightarrow \left( 0 \right| \end{aligned}$$

Each monomial $z^{\textrm{n}}$ is denoted as a *number state*

155 $$\begin{aligned} \prod _{i = 1}^D z_i^{{\textrm{n}}_i} \times 1&\rightarrow \prod _{i = 1}^D { a_i^{\dagger } }^{{\textrm{n}}_i} \left| 0 \right) \equiv \left| \textrm{n}\right) . \end{aligned}$$

The number states are eigenstates of the set of *number operators* ${\hat{n}}_i \equiv a^{\dagger }_i a^i$ (no Einstein sum):

156 $$\begin{aligned} {\hat{n}}_i \left| \textrm{n}\right) = {\textrm{n}}_i \left| \textrm{n}\right) . \end{aligned}$$

Dual to each number state is a conjugate projection operator

157 $$\begin{aligned} \left( \textrm{m} \right|&\equiv \left( 0 \right| \prod _{i = 1}^D \frac{ { a_i^{\dagger } }^{\textrm{m}_i} }{ \textrm{m}_i ! } \leftarrow \int d^D \! z \, {\delta }^D \! \left( z \right) \prod _{i = 1}^D \frac{ { \left( \partial / \partial z_i \right) }^{ \textrm{m}_i } }{ \textrm{m}_i ! } \end{aligned}$$

From the commutation relations of variables and their derivatives it follows that the number states and projectors have overlap

158 $$\begin{aligned} \left( \textrm{m} \, \right| \! \left. \textrm{n}\right) = {\delta }^D_{\textrm{m} \textrm{n}} \end{aligned}$$

the Kronecker $\delta$ symbol on *D* indices. The number states and projectors are complete, and a sum of Eq. (158) on $\textrm{m}$ is the *Glauber norm*

159 $$\begin{aligned} \left( 0 \right| e^{\sum _i a_i} \left| \textrm{n}\right) = 1 , \quad \forall \textrm{n}, \end{aligned}$$

which defines the asymmetric inner product on the Hilbert space of generating functions.

Replacing the uniform measure $\sum _i a_i \equiv 1^T a$ in Eq. (159) with the scalar product *za* gives the map (33) to $\Psi \! \left( z \right)$ in the main text. $\Psi \! \left( 1 \right) \equiv 1$; the Glauber norm of the Laplace transform of any normalized distribution is the trace of the probability distribution $\sum _{\textrm{n}} {\rho }_{\textrm{n}}$.

### Coherent states and Peliti functional integral

The uniform measure in the 2D-dimensional integral for the representation of unity (42) in the main text is known as the Haar measure. Using the definition (38) for coherent states and (39) for their dual projectors, and expanding the exponential functions as sums,

160 $$\begin{aligned} \int \frac{ d^D \! {\phi }^{\dagger } d^D \! \phi }{ {\pi }^D } \left| \phi \right) \left( \phi \right|= & {} \int \frac{ d^D \! {\phi }^{\dagger } d^D \! \phi }{ {\pi }^D } \prod _{i = 1}^D e^{- {\phi }^{\dagger }_i {\phi }_i} \sum _{{\textrm{n}}_i} \sum _{\textrm{m}_i} \frac{ {\phi }_i^{{\textrm{n}}_i} {{\phi }^{\dagger }_i}^{\textrm{m}_i} }{ {\textrm{n}}_i ! } \left| \textrm{n}\right) \left( \textrm{m} \right| \nonumber \\= & {} \sum _{\textrm{n}} \left| \textrm{n}\right) \left( \textrm{n}\right| = I \end{aligned}$$

The phase component in each integral $d{\phi }^{\dagger }_i \, d{\phi }_i$ vanishes unless ${\textrm{n}}_i = \textrm{m}_i$, and the remaining modulus component produces a Gamma-function canceling the ${\textrm{n}}_i !$. Thus the Haar measure on coherent states is equivalent to the uniform measure on states $\textrm{n}$ of the classical probability distribution.^7^

Mapping backward through the correspondences between analytic functions and Doi state vectors from Appendix C.1, an evaluation of the integral in terms of Dirac $\delta$-functions^8^ shows that the effect of the representation of unity is the map

161 $$\begin{aligned} \int \frac{ d^D {\phi }^{\dagger } d^D \phi }{ {\pi }^D } \left| \phi \right) \left( {\phi }^{\dagger } \right|\leftarrow & {} \int \frac{ d^D {\phi }^{\dagger } d^D \phi }{ {\pi }^D } e^{z^{\prime } \phi } 1 \int d^D \! z \, {\delta }^D \! \left( z \right) e^{ {\phi }^{\dagger } \left( \partial / \partial z - \phi \right) } \nonumber \\= & {} \int d^D \! z \, {\delta }^D \! \left( z \right) e^{z^{\prime } \partial / \partial z} = z \mapsto z^{\prime } \end{aligned}$$

To keep track of the scoping rules for application of complex functions and derivatives would require introducing a distinct set of variables $z_{k \delta t}$ for each interval in the quadrature (34). In the Doi operator algebra this scoping is handled by the bracket inner product, and the map (161) becomes simply the identity map on $a^{\dagger }$ and *a*.

The integration measure that results from inserting a copy of the representation of unity (42) between each interval of time evolution in Eq. (34) is called a skeletonized measure. Its limit as the interval length $\delta t \rightarrow 0$

162 $$\begin{aligned} \int _0^T {\mathcal {D}}^D \! {\phi }^{\dagger } {\mathcal {D}}^D \! {\phi } \equiv \lim _{\delta t \rightarrow 0} \prod _{k = 1}^{T / \delta t} \int \frac{ d^D \! {\phi }^{\dagger }_{k \delta t} d^D \! {\phi }_{k \delta t} }{ {\pi }^D } \end{aligned}$$

defines the functional integration measure used in Eq. (43) and elsewhere.

## Gaussian-order fluctuations, diffusions, and stochastic differential equations

The Doi-Peliti construction used in this paper affords a point of departure to many other representations of perturbed dynamical systems. It is important to emphasize that the motivation and form of 2FFI representations does not depend on the integer-lattice state space and exponential generating function that enable the specific Doi-Peliti construction. To show this we reproduce from [42, 68] a variety of reductions from the full field theory to the limit of Gaussian-order fluctuations that is specified by a drift velocity and diffusion tensor that are functions only of the configuration variable *n*. These are “classical” representations of perturbed dynamical systems ostensibly lacking two-field structure. We will show how the inverse of the steps reducing Doi-Peliti to these classical limits has been used in [30, 52] to construct two-field theories or their large-deviation asymptotics in the form produced by the Doi-Peliti 2FFI. These classical descriptions from functions on *n* no longer reference lattice state spaces or population processes, and the 2FFI theories to which they correspond, like the operator formulation of [30, 52] are expected to apply to much more general perturbed dynamical systems.

The Gaussian-order reduction of the general action (46) is obtained by writing the pair $\left( \theta , n \right) = \left( \bar{\theta }, \bar{n} \right) + \left( {\theta }^{\prime }, n^{\prime } \right)$, where $\left( \bar{\theta }, \bar{n} \right)$ are a stationary trajectory, and expanding *S* to quadratic order in primed fields as

163 $$\begin{aligned}{} & {} S - \bar{S} \approx \int _0^T dt \left\{ {\theta }^{\prime } \left( d_t + \overline{ \frac{ {\partial }^2 \mathcal {L} }{ \partial \theta \, \partial n } } \right) n^{\prime } + \frac{1}{2} {\theta }^{\prime } \overline{ \frac{ {\partial }^2 \mathcal {L} }{ \partial \theta \, \partial {\theta } } } {{\theta }^{\prime }}^T + \frac{1}{2} {n^{\prime }}^T \overline{ \frac{ {\partial }^2 \mathcal {L} }{ \partial n^T \partial n } } n^{\prime } \right\} . \end{aligned}$$

Overbar in Eq. (163) indicates evaluation of *S* or the derivatives of $\mathcal {L}$ at $\left( \bar{\theta }, \bar{n} \right)$.

The Hessian $\overline{ {\partial }^2 \mathcal {L} / \partial \theta \, \partial {\theta } }$ is negative-definite and time-local, so the functional determinant of its time integral may be written as a Gaussian integral over a newly-introduced auxiliary field $\xi$ with integrand $e^{- S_\textrm{aux}}$, where

164 $$\begin{aligned} S_\textrm{aux}&\approx \int _0^T dt \left\{ \frac{1}{2} \left( {\xi }^T - {\theta }^{\prime } \overline{ \frac{ {\partial }^2 \mathcal {L} }{ \partial \theta \, \partial {\theta } } } \right) { \left( - \overline{ \frac{ {\partial }^2 \mathcal {L} }{ \partial \theta \, \partial {\theta } } } \right) }^{-1} \left( \xi - \overline{ \frac{ {\partial }^2 \mathcal {L} }{ \partial {\theta } \partial \theta } } {{\theta }^{\prime }}^T \right) \right\} . \end{aligned}$$

We may insert the number 1 into the path integral (43) by inserting the explicit functional integral over $\xi$ and dividing by the determinant that is its evaluation, producing a new functional integral with integrand $e^{- \left( S + S_\textrm{aux} \right) }$. The quadratic-order expansion of *S* shifted by the auxiliary-field action satisfies

165 $$\begin{aligned}&S - \bar{S} + S_\textrm{aux} \nonumber \\ {}&\quad \approx \int _0^T dt \left\{ {\theta }^{\prime } \left[ \left( d_t + \overline{ \frac{ {\partial }^2 \mathcal {L} }{ \partial \theta \, \partial n } } \right) n^{\prime } - \xi \right] + \frac{1}{2} \xi { \left( - \overline{ \frac{ {\partial }^2 \mathcal {L} }{ \partial \theta \, \partial {\theta } } } \right) }^{-1} {\xi }^T + \frac{1}{2} {n^{\prime }}^T \overline{ \frac{ {\partial }^2 \mathcal {L} }{ \partial n^T \partial n } } n^{\prime } \right\} . \end{aligned}$$

The action (165) is linear in ${\theta }^{\prime }$, meaning that the functional integral over ${\theta }^{\prime }$ along the imaginary axis at each time-index *t* produces a functional Dirac $\delta$-function for the quantity multiplying ${\theta }^{\prime }$. The remaining terms are quadratic forms in $\xi$ and $n^{\prime }$. In general, the $\delta$-functional produced by integration over ${\theta }^{\prime }$ connects these two fields by a time derivative, so the implied fluctuation distribution is not simple to compute. However, about ordinary relaxation trajectories that are the classical solutions to the equations of motion, for which $\bar{\theta } = 0$, both $\bar{S} = 0$ and also $\overline{ {\partial }^2 \mathcal {L} / \partial n^T \partial n } = 0$. (See [42] for extensive explanations of why this is the case in general, and of its interpretation in terms of causality.) For this case the functional integral (43) (which we evaluate at $z = 1$ for simplicity), becomes

166 $$\begin{aligned} 1&= \int _0^T {\mathcal {D}}^D \! \xi \, {\mathcal {D}}^D \! n \, e^{ \left( e^{-{\theta }_T} - 1 \right) n_T } \nonumber \\ {}&\quad \times \exp \left( \frac{1}{2} \int _0^T dt \xi { \left( - \overline{ \frac{ {\partial }^2 \mathcal {L} }{ \partial \theta \, \partial {\theta } } } \right) }^{-1} {\xi }^T \right) \delta \! \left[ \left( d_t + \overline{ \frac{ {\partial }^2 \mathcal {L} }{ \partial \theta \, \partial n } } \right) n^{\prime } - \xi \right] e^{ {\psi }_0 \left( \log {\phi }^{\dagger }_0 \right) } . \end{aligned}$$

The integral (166) is over an ensemble of trajectories each satisfying

167 $$\begin{aligned} \left( d_t + \overline{ \frac{ {\partial }^2 \mathcal {L} }{ \partial \theta \, \partial n } } \right) n^{\prime }&= \xi . \end{aligned}$$

Eq. (167) is the approximation to linear order in the perturbing field $\xi$ of the stochastic differential equation

168 $$\begin{aligned} \frac{dn}{dt} + { \left. \frac{ \partial \mathcal {L} }{ \partial \theta } \right| }_{\theta = 0}&= \xi . \end{aligned}$$

Because the sole remaining Gaussian kernel in Eq. (166) is time-local, $\xi$ is a Langevin white noise with correlation function

169 $$\begin{aligned} \left\langle {\xi }_t {\xi }^T_{t^{\prime }} \right\rangle&= \left( - { \left. \frac{ {\partial }^2 \mathcal {L} }{ \partial \theta \, \partial {\theta } } \right| }_{\theta = 0} \right) \delta \! \left( t - t^{\prime } \right) . \end{aligned}$$

The noise covariance (169), although expressed in terms of the value $\bar{n}$ along a stationary trajectory at $\theta = 0$, may be understood as a function of the local coordinates *n* in the underlying manifold, since for classical solutions stationary trajectories form a foliation of the configuration manifold.

The Gaussian-order fluctuation limit corresponding to the Langevin equation (168, 169) produces, for the continuum limit of the lattice of states $\textrm{n} = \left( \textrm{n}_i \right)$, the diffusion equation for a density indexed by continuous index $n = \left( n_i \right)$, the master equation

170 $$\begin{aligned} \left\{ \frac{\partial }{\partial t} - \frac{\partial }{\partial n_i} \left( { \left. \frac{ \partial \mathcal {L} }{ \partial {\theta }_i } \right| }_{\theta = 0} \right) + \frac{1}{2} \frac{{\partial }^2}{\partial n_i \partial n_j} \left( { \left. \frac{ {\partial }^2 \mathcal {L} }{ \partial {\theta }_j \partial {\theta }_i } \right| }_{\theta = 0} \right) \right\} {\rho }_n \end{aligned}$$

known as the *Fokker–Planck equation* [74]. (This form may be obtained directly from the transition matrix corresponding to the Liouville operator—for example, as written in [44, 69, 71] for chemical reaction networks—by writing discrete shifts of the index $\textrm{n}_i$ as if they were exponentiated operators $e^{\partial / \partial n_i}$ acting on a continuously-indexed density ${\rho }_n$, and then “expanding the Taylor’s series for the exponential” to second order.)

Here we have derived the three equivalent representations of the Gaussian-fluctuation limit specified entirely by a drift velocity and diffusion kernel as functions of *n*—the quadratic action (163), the Langevin equation (168, 169), and the Fokker–Planck equation (170)—from a Doi-Peliti starting point. The reverse of the derivation from Eqs. (163–170) may be followed to arrive at either two-field forms or their large-deviation asymptotics starting from the stochastic differential equation or Fokker–Planck equation. Kamenev [42] has shown that the “tri-diagonal” arrangement of response and correlation functions in Eq. (4.1) of [52], equivalent to the quadratic action (163) here, is in fact the common motif in 2FFI for stochastic classical mechanics, and for the Schwinger-Keldysh time-loop in quantum mechanics [43, 63], allowing us to bypass the operator formalism altogether and proceed directly from stochastic differential equations (168, 169) to the path integral up to Gaussian order. For a development of correspondence principles, where both the quantum Schwinger-Keldysh time loop and the stochastic two-field integral appear, see [67].

It is central to the aims of [52] that the tri-diagonal response and correlation functions are not limited to the “bare” constitutive parameters of the underlying stochastic differential equation, but rather are a general form for Gaussian order fluctuations in an *effective theory* where the drift velocity and fluctuation covariance are generally renormalized quantities, in this way subsuming higher-order fluctuation effects, which may appear explicitly in the Doi-Peliti action. Renormalization of the stochastic action as an effective action may also be done directly from the path integral with diagrammatic methods of the type appearing in [52]. For an application to evolutionary games see [70, Ch. 7].

Alternatively, the large-deviation asymptotics for excursions within, or for escapes from, a domain, may be derived directly from the stochastic differential equation, and related to boundary-value problems for solutions of diffusions such as the Fokker–Planck equation. This program, starting from representations identical to Eqs. (168–170) for Gaussian fluctuations, and covering several other classes of perturbations as well, is carried out by Freidlin and Wentzell [30], and has been applied to a wide range of first-passage and escape problems [48–51].

The connection to 2FFI methods is that an action arises as their large-deviation function from the drift and diffusion functions of the Gaussian limit, which is simply obtained from the Doi-Peliti action. By completing the square in ${\theta }^{\prime }$ (which is $\theta$ in a neighborhood of $\bar{\theta } = 0$), the quadratic action (163) is put into the form

171 $$\begin{aligned} S \rightarrow&\rightarrow \frac{1}{2} \int _0^T dt \left\{ { \left[ \left( d_t + \overline{ \frac{ {\partial }^2 \mathcal {L} }{ \partial \theta \, \partial n } } \right) n^{\prime } \right] }^T { \left( - \overline{ \frac{ {\partial }^2 \mathcal {L} }{ \partial \theta \, \partial {\theta } } } \right) }^{-1} \left[ \left( d_t + \overline{ \frac{ {\partial }^2 \mathcal {L} }{ \partial \theta \, \partial n } } \right) n^{\prime } \right] \right\} \nonumber \\&+ \text{ quadratic } \text{ in } \theta \end{aligned}$$

The quadratic form in $\theta$ is integrated out in the functional integral to produce a functional determinant that depends on the background $\bar{n}$ only logarithmically. Again, the term in $n^{\prime }$ in Eq. (171) is the leading-linear expansion of the equations of motion in small fluctuations, so at the Gaussian order to which it is defined, Eq. (171) may be approximated as

172 $$\begin{aligned} S \rightarrow&\approx \frac{1}{2} \int _0^T dt \left\{ { \left( \frac{dn}{dt} + { \left. \frac{ \partial \mathcal {L} }{ \partial \theta } \right| }_{\theta = 0} \right) }^T { \left( - { \left. \frac{ {\partial }^2 \mathcal {L} }{ \partial \theta \, \partial {\theta } } \right| }_{\theta = 0} \right) }^{-1} \left( \frac{dn}{dt} + { \left. \frac{ \partial \mathcal {L} }{ \partial \theta } \right| }_{\theta = 0} \right) \right\} \end{aligned}$$

a form first due to Onsager and Machlup [55], and appearing in Theorem 1.1 on p. 86 of [30].

Two-field constructions originating in classical perturbed dynamical systems (168–170) in the continuous variable *n* may readily be extended to continuously-indexed state spaces with topologies, on which the drift velocity may include spatial gradients, as in the case of reaction-diffusion systems, without altering the program for construction of the corresponding two-field representation.

## Stationary-point approximations to the Wigner function

The functional integral provides the most direct route to the current conservation law (62) for the Wigner function. It is possible, with somewhat more work, to derive the same relations directly from stationary variation of the generating function, and in the process to gain some more intuition for what the Wigner function quantifies.

### The Wigner function in terms of an explicit density over coherent-state parameters

Begin by writing any state vector as the integral of a density in the coherent-state basis:

173 $$\begin{aligned} \left| \Psi \right) \equiv \int d^D \! \phi \, \left| \phi \right) \rho \! \left( \phi \right) \end{aligned}$$

The generalized Glauber norm (33) returns the analytic representation of the MFG:

174 $$\begin{aligned} \Psi \! \left( z \right)&= \left( 0 \right| e^{z a} \left| \Psi \right) \nonumber \\&= \int d^D \! \phi \, \left( 0 \right| e^{z a} \left| \phi \right) \rho \! \left( \phi \right) \nonumber \\&= \int d^D \! \phi \, e^{\left( z - 1 \right) \phi } \rho \! \left( \phi \right) \end{aligned}$$

Now evaluate the integral in Eq. (59) at time $t \rightarrow T$, where the stationary value of the field ${\phi }^{\dagger }$ will coincide with the imposed argument *z*,

175 $$\begin{aligned} w_T \! \left( {\phi }^{\dagger }_{\ddagger } , {\phi }_{\ddagger } \right)&= \frac{1}{{\pi }^D} \int d^D \! \phi \, e^{ \left( {\phi }^{\dagger }_{\ddagger } - z \right) \left( \phi - {\phi }_{\ddagger } \right) } e^{\left( z - 1 \right) \phi } \rho \! \left( \phi \right) \end{aligned}$$

It follows then that

176 $$\begin{aligned} \int d^D \! {\phi }^{\dagger }_{\ddagger } \, w_T \! \left( {\phi }^{\dagger }_{\ddagger } , {\phi }_{\ddagger } \right) = e^{\left( z - 1 \right) {\phi }_{\ddagger }} \rho \! \left( {\phi }_{\ddagger } \right) \end{aligned}$$

A second integral over the ${\phi }_{\ddagger }$ fields yields the two equivalent expressions (60) and (174).

### Stationary-point approximations

The stationary value $\bar{\phi } \! \left( z \right)$ of the tilted density $e^{\left( z-1 \right) \phi } \rho \! \left( \phi \right)$ is given by

177 $$\begin{aligned} { \left. \frac{\partial \log \rho }{\partial \phi } \right| }_{\bar{\phi } \left( z \right) } = 1 - z \end{aligned}$$

The two stationarity conditions on the arguments of $w_T$ follow from Eq. (175) as

178 $$\begin{aligned} { \left. \frac{\partial }{\partial {\phi }_{\ddagger }} w_T \! \left( {\phi }^{\dagger }_{\ddagger } , {\phi }_{\ddagger } \right) \right| }_{{\phi }^{\dagger }_{\ddagger } = z}&= \left( z - {\phi }^{\dagger }_{\ddagger } \right) w_T \! \left( z, {\phi }_{\ddagger } \right) \nonumber \\ { \left. \frac{\partial }{\partial {\phi }^{\dagger }_{\ddagger }} w_T \! \left( {\phi }^{\dagger }_{\ddagger } , {\phi }_{\ddagger } \right) \right| }_{{\phi }^{\dagger }_{\ddagger } = z}&= \int d^D \! \phi \, \left( \phi - {\phi }_{\ddagger } \right) e^{\left( z - 1 \right) \phi } \rho \! \left( \phi \right) \sim \left( \bar{\phi } - {\phi }_{\ddagger } \right) w_T \! \left( z, {\phi }_{\ddagger } \right) \end{aligned}$$

where the stationary-point approximation (177) to the mean gives the leading exponential approximation in the second expression.

The Wigner function in Eq. (175), at argument *z*, exactly equals integral (174)

179 $$\begin{aligned} w_T \! \left( z, {\phi }_{\ddagger } \right) = \Psi \! \left( z \right) \sim { \left. e^{\left( z - 1 \right) \phi } \rho \! \left( \phi \right) \right| }_{\bar{\phi } \left( z \right) } \end{aligned}$$

independent of the value of ${\phi }_{\ddagger }$. While the first line of Eq. (178) shows that *z* is a stationary-point argument for ${\phi }^{\dagger }_{\ddagger }$, the second line shows that only when ${\phi }_{\ddagger } = \bar{\phi } \! \left( z \right)$ is the other argument also a stationary value.

### Time dependence along a stationary path

Suppose now that from such a compatible pair $\left( z , \bar{\phi } \! \left( z \right) \right)$, we wish to extend *z* and $\bar{\phi }$ along a trajectory that preserves stationarity. The total time derivative of $w_T$ with respect to its final-time argument is given by

180 $$\begin{aligned} \frac{d}{dT} w_T \! \left( {\phi }^{\dagger }_{\ddagger } , {\phi }_{\ddagger } \right)&= \left\{ \frac{dz}{dT} {\phi }_{\ddagger } + \frac{d {\phi }^{\dagger }_{\ddagger } }{dT} \frac{ \partial }{ \partial {\phi }^{\dagger }_{\ddagger } } + \frac{d {\phi }_{\ddagger } }{dT} \frac{ \partial }{ \partial {\phi }_{\ddagger }} \right\} w_T \! \left( {\phi }^{\dagger }_{\ddagger } , {\phi }_{\ddagger } \right) \nonumber \\&\quad - \frac{1}{{\pi }^D} \int d^D \! \phi \, e^{ \left( {\phi }^{\dagger }_{\ddagger } - z \right) \left( \phi - {\phi }_{\ddagger } \right) } \mathcal {L} \! \left( {\phi }^{\dagger }_{\ddagger } , \phi \right) e^{\left( z - 1 \right) \phi } \rho \! \left( \phi \right) \end{aligned}$$

(Note that Eq. (180) includes only contributions to the derivative of $w_T$ from quantities defined before time *T*; this derivative is different from the total derivative *d*/*dt* of $w_t$ in the functional integral (59), which also includes effects of the functional integral after time *t*.)

Ensuring that $z + dt \left( dz / dt \right)$ is a stationary value if *z* is one requires that

181 $$\begin{aligned} 0&= { \left. \frac{\partial }{\partial {\phi }_{\ddagger }} \frac{d}{dt} w_T \! \left( {\phi }^{\dagger }_{\ddagger } , {\phi }_{\ddagger } \right) \right| }_{{\phi }^{\dagger }_{\ddagger } = z} \sim \left\{ \frac{dz}{dt} - { \left. \frac{\partial }{\partial \phi } \mathcal {L} \! \left( z , \phi \right) \right| }_{\bar{\phi } \left( z \right) } \right\} w_T \! \left( z , {\phi }_{\ddagger } \right) \end{aligned}$$

The term $\partial \mathcal {L} / \partial \phi$ is obtained by an integration by parts over $d^D \! \phi$, and evaluated in the stationary-point approximation. All other terms from Eq. (180) vanish at ${\phi }^{\dagger }_{\ddagger } = z$. Thus preservation of the stationary-argument condition for ${\phi }_{\ddagger }$ gives the stationary-path equation for *dz*/*dt*.

To identify the time-dependence of the stationary argument ${\phi }_{\ddagger }$, we work directly from the stationary-point condition (177). The total time derivative of that equation is

182 $$\begin{aligned} - { \left. \frac{\partial \mathcal {L}}{\partial \bar{\phi }} \right| }_z&= \frac{d}{dt} \left( { \left. \frac{\partial \log \rho }{\partial \phi } \right| }_{\bar{\phi } \left( z \right) } \right) \nonumber \\&= \frac{d \bar{\phi }}{dt} \left( { \left. \frac{{\partial }^2 \log \rho }{\partial {\phi }^2} \right| }_{\bar{\phi }} \right) + { \left. \frac{\partial }{\partial \bar{\phi }} \right| }_t \left( { \left. \frac{ \partial \log \rho \! \left( \phi \right) }{ \partial t } \right| }_{\bar{\phi } \left( z \right) } \right) \nonumber \\&= - \frac{d \bar{\phi }}{dt} { \left. \frac{\partial z}{\partial \bar{\phi }} \right| }_t - { \left. \frac{\partial }{\partial \bar{\phi }} \right| }_t { \left. \mathcal {L} \! \left( z , \bar{\phi } \right) \right| }_{z \left( \bar{\phi } \right) } \nonumber \\&= - { \left. \frac{\partial z}{\partial \bar{\phi }} \right| }_t \left( \frac{d \bar{\phi }}{dt} + { \left. \frac{ \partial \mathcal {L} }{ \partial z } \right| }_{\bar{\phi }} \right) - { \left. \frac{ \partial \mathcal {L} }{ \partial \bar{\phi } } \right| }_z \end{aligned}$$

In passing from the second to the third line of Eq. (182), to obtain an explicit expression for ${\left. \partial \log \rho \! \left( \phi \right) / \partial t \right| }_{\bar{\phi }}$ in terms of $\mathcal {L} \! \left( z , \bar{\phi } \right)$, we evaluate *z* as an inverse function of $\bar{\phi }$ from Eq. (177). This functional dependence contributes the term $\partial \mathcal {L} / \partial z$ in the final line, from which we obtain the stationary-path equation for the trajectory of $\bar{\phi } \! \left( z \right)$ along which ${\phi }_{\ddagger }$ is to be evaluated:

183 $$\begin{aligned} \frac{d\bar{\phi }}{dt}&= - \frac{\partial }{\partial z} \mathcal {L} \! \left( z , \bar{\phi } \right) \end{aligned}$$

Equations (181) and (183) imply the 2FFI counterpart to conservation of energy in Hamiltonian mechanics: $d \mathcal {L} / dt = 0$ along the stationary path if $\partial \mathcal {L} / \partial t = 0$.

### The stochastic effective action in stationary-path evaluations

Note from Eq. (180) that along the contour identified to preserve stationarity of $w_T$,

184 $$\begin{aligned} \frac{d}{dT} \left( { \left. w_T \! \left( z , \bar{\phi } \! \left( z \right) \right) \right| }_{z \left( T \right) } \right)&= \frac{dz}{dT} \bar{\phi } - \mathcal {L} \! \left( z , \bar{\phi } \right) w_T \! \left( z , \bar{\phi } \right) \nonumber \\&= \frac{d}{dT} \int _T dt \left\{ - \frac{dz}{dt} \bar{\phi } + \mathcal {L} \! \left( z , \bar{\phi } \right) \right\} \nonumber \\&\equiv \frac{d}{dT} {\bar{S}}_T \end{aligned}$$

Therefore the extension of the Wigner function to times $t > T$ must include the stationary-path contribution from the action, which was present for $t < T$ in the functional integral definition (59). $w_T$ thus extended satisfies

185 $$\begin{aligned} \frac{d}{dt} \left( e^{- {\bar{S}}_T} w_T \! \left( z , \bar{\phi } \right) \right) \sim 0 \end{aligned}$$

recovering Eq. (62).

We have termed the stationary-path evaluation of *S* the *stochastic effective action* [68]. It is the functional Legendre transform of the large-deviation functional for trajectories in Doi-Peliti integrals. The approximation (179), with ${\phi }_{\ddagger }$ set equal to $\bar{\phi } \! \left( z \right)$ given $\rho \! \left( \phi \right)$, together with the contribution from $S_T$ in Eq. (185), provides the desired interpretation of the Wigner function in terms of densities in the statistical model provided by coherent states, and their exponential tilts by likelihood functions.

## Stationary-path solutions for the two-state system

The stationary-path equations and both initial and final values for fields are obtained from vanishing of all terms in the variational derivative of the exponential argument in Eq. (43). We begin with solutions in coherent-state variables, and then present the forms for the descaled number coordinates $\nu$, $\underline{\nu }$, and $\bar{\nu }$.

### Coherent-state and number-potential solutions

#### Stationary-path equations and final-time conditions for response fields

The stationary-path equations of motion for the components of the field ${\phi }^{\dagger }$ from Eq. (47), in the rotated basis (100), evaluate to

186 $$\begin{aligned} {\partial }_{\tau } {\Phi }^{\dagger }&= \frac{\partial \hat{\mathcal {L}}}{\partial \hat{\Phi }} = - {\varvec{\nu }} {\phi }^{\dagger }&{\partial }_{\tau } {\phi }^{\dagger }&= \frac{\partial \hat{\mathcal {L}}}{\partial \hat{\phi }} = {\phi }^{\dagger } \end{aligned}$$

The final-time values ${\phi }^{\dagger }_T$ are given by variation of ${\hat{\phi }}_T$, as

187 $$\begin{aligned} {\phi }^{\dagger }_a&= z_a&{\phi }^{\dagger }_b&= z_b \end{aligned}$$

Fixing the magnitude of the combination ${\phi }^{\dagger }_b {\varvec{\nu }}_b + {\phi }^{\dagger }_a {\varvec{\nu }}_a$ in Eq. (104) requires varying *z* along the contour

188 $$\begin{aligned} z_a&\equiv \frac{{\underline{\nu }}_{Ta}}{{\varvec{\nu }}_a}&z_b&\equiv \frac{{\underline{\nu }}_{Tb}}{{\varvec{\nu }}_b} \end{aligned}$$

giving Eq. (113) in the main text. The remaining time-dependent solutions, with time argument denoted explicitly here by subscript $\tau$, are given by

189 $$\begin{aligned} {\phi }^{\dagger }_{\tau }&= \frac{ \left( {\underline{\nu }}_T - {\varvec{\nu }} \right) }{ \left( \frac{1}{4} - {\varvec{\nu }}^2 \right) } e^{\tau - T}&{\Phi }^{\dagger }_{\tau }&= 1 - {\varvec{\nu }} {\phi }^{\dagger }_{\tau } \end{aligned}$$

Initial data are specified in the generating function ${\psi }_0$, which when evaluated at the solutions for ${\phi }^{\dagger }_0$ become

190 $$\begin{aligned} \frac{1}{N} {\psi }_0 \left( \log {{\phi }^{\dagger }_a}_0 , \log {{\phi }^{\dagger }_b}_0 \right)&= \log \left( {{\phi }^{\dagger }_a}_0 {\underline{\nu }}_{0a} + {{\phi }^{\dagger }_b}_0 {\underline{\nu }}_{0b} \right) \nonumber \\&= \log \left( {\Phi }^{\dagger }_0 + {\bar{\nu }}_0 {\phi }^{\dagger }_0 \right) \nonumber \\&= \log \left( 1 + \Lambda e^{-T} \right) \end{aligned}$$

The final line of Eq. (190) is obtained by combining the two solutions (189) at $\tau = 0$, and introduces the combination $\Lambda$ defined in Eq. (106).

#### Stationary-path equations and initial-time conditions for observable fields

The stationary-path equations of motion for the components of the field $\phi$ from Eq. (47) are obtained by removing a total derivative $d_t \left( {\phi }^{\dagger } \phi \right)$ from the action (44) to shift the derivative onto $\phi$. In the rotated basis (100), they evaluate to

191 $$\begin{aligned} {\partial }_{\tau } \hat{\Phi }&= - \frac{\partial \hat{\mathcal {L}}}{\partial {\Phi }^{\dagger }} = 0&{\partial }_{\tau } \hat{\phi }&= - \frac{\partial \hat{\mathcal {L}}}{\partial {\phi }^{\dagger }} = - \left( \hat{\phi } - {\varvec{\nu }} \hat{\Phi } \right) \end{aligned}$$

The total derivative cancels the final-time term ${\left( {\phi }^{\dagger } \phi \right) }_T$ from the exponential in Eq. (43) and introduces an initial-time term ${\left( {\phi }^{\dagger } \phi \right) }_0$. Variation of this term against ${\psi }_0$ with respect to ${\phi }^{\dagger }_0$ gives the initial-value conditions for the components of ${\hat{\phi }}_0$, as

192 $$\begin{aligned} {\hat{\phi }}_{0a}&= \frac{ \partial {\psi }_0 }{ \partial {\phi }^{\dagger }_{0a} }&{\hat{\phi }}_{0b}&= \frac{ \partial {\psi }_0 }{ \partial {\phi }^{\dagger }_{0b} } \end{aligned}$$

Solutions to the equations of motion (191) from these initial conditions are then

193 $$\begin{aligned} {\hat{\Phi }}_t&= {\hat{\Phi }}_0 = \frac{ 1 }{ 1 + \Lambda e^{-T} }&{\hat{\phi }}_t&= {\hat{\Phi }}_0 \left[ {\varvec{\nu }} + \left( {\bar{\nu }}_0 - {\varvec{\nu }} \right) e^{- \tau } \right] \end{aligned}$$

The two displacements defined in Eqs. (104) and (105), characterizing respectively the mean in the nominal distribution and the likelihood ratio applied to the stationary measure, evaluate to

194 $$\begin{aligned} {\bar{\nu }}_{\tau }&= {\varvec{\nu }} + \left( {\bar{\nu }}_0 - {\varvec{\nu }} \right) e^{-\tau }&{\underline{\nu }}_{\tau }&= {\varvec{\nu }} + \left( {\underline{\nu }}_T - {\varvec{\nu }} \right) e^{\tau - T} \end{aligned}$$

These results are reproduced (dropping the explicit subscripts $\tau$) as Eq. (107) in the text. It follows from Eq. (194) that the combination

195 $$\begin{aligned} \frac{ \left( {\underline{\nu }}_{\tau } - {\varvec{\nu }} \right) \left( {\bar{\nu }}_{\tau } - {\varvec{\nu }} \right) }{ \left( \frac{1}{4} - {\varvec{\nu }}^2 \right) } = \Lambda e^{-T} \end{aligned}$$

is invariant at its initial value.

The CGF for a binomial distribution at any time retains the form (92), with ${\underline{\nu }}_a / {\varvec{\nu }}_a$ and ${\underline{\nu }}_b / {\varvec{\nu }}_b$ replacing $z_a$ and $z_b$, and ${\bar{\nu }}_a$ and ${\bar{\nu }}_b$ replacing ${\nu }_a$, and ${\nu }_b$. From Eq. (190) and the invariant form (195), it follows that

196 $$\begin{aligned} \frac{{\psi }_{\tau }}{N} = \log \left[ 1 + \Lambda e^{-T} \right] = -\log {\hat{\Phi }}_0 \end{aligned}$$

giving Eq. (109) in the text.

Finally, the non-linear mean of sample values (102) in the importance distribution can be shown to evaluate to

197 $$\begin{aligned} {\nu }_{\tau }&= {\underline{\nu }}_{\tau } + \frac{ \left( \frac{1}{4} - {\underline{\nu }}_{\tau }^2 \right) }{ \left( \frac{1}{4} - {\varvec{\nu }}^2 \right) } \left[ \left( {\bar{\nu }}_{\tau } - {\varvec{\nu }} \right) {\hat{\Phi }}_0 \right] \end{aligned}$$

from which the form (111) for $\partial \nu / \partial \theta$ can be derived. The ratio of measures  in Eq. (197), by which the importance distribution responds to variations in the initial data, is the familiar scaling of response functions [68] in the Fluctuation-Dissipation Theorem, because expressions of the form  are the variance of fluctuations in the binomial.

### Fisher spherical embedding

In one dimension, the Pythagorean theorem (12) for K-L divergences loses the interpretation of a direction cosine between vector fields, but still reflects scale changes between coherent-state or exponential families and the geometric coordinate.

The mean-value Fisher-sphere construction of Sect. A.2, for one variable, is the embedding on a circle:

198 $$\begin{aligned} \cos ^2 \alpha&\equiv \frac{1}{2} + \nu&\sin ^2 \alpha&\equiv \frac{1}{2} - \nu \end{aligned}$$

The coordinate differential is

199 $$\begin{aligned} 2 \frac{d \alpha }{d \theta } = \sqrt{ \frac{1}{4} - {\nu }^2 } \end{aligned}$$

and the geometric distance element is then

200 $$\begin{aligned} 4 { \left( \delta \alpha \right) }^2 = \left( \frac{1}{4} - {\nu }^2 \right) { \left( \delta \theta \right) }^2 = \frac{\partial \nu }{\partial \theta } { \left( \delta \theta \right) }^2 \end{aligned}$$

The ${\hat{\Phi }}_0^2$ term in Eq. (124) becomes

201 $$\begin{aligned} {\hat{\Phi }}_0^2 = \frac{ 1 }{ { \left[ 1 + u v \right] }^2 }&= \frac{ \left( \frac{1}{4} - {\nu }^2 \right) }{ \left( \frac{1}{4} - {\underline{\nu }}^2 \right) \left( \frac{1}{4} - {\bar{\nu }}^2 \right) } \nonumber \\&= 4 \frac{ \left( \partial \alpha / \partial \theta \right) \left( \partial \alpha / \partial \eta \right) }{ \left( \partial v / \partial \theta \right) \left( \partial u / \partial \eta \right) } \nonumber \\&= 4 \frac{\partial \alpha }{\partial v} \frac{\partial \alpha }{\partial u} \end{aligned}$$

the invariant Fisher information in coherent-state coordinates. The equivalence between the two forms (69) and (71) for the Fisher metric is again recovered as

202 $$\begin{aligned} \frac{ {\partial }^2 }{ \partial {\theta }^2 } \left( \frac{{\psi }}{N} \right) = 4 \frac{\partial \alpha }{\partial \theta } \frac{\partial \alpha }{\partial \eta } \end{aligned}$$

showing the variation of the embedding coordinate of the importance distribution with the tilt multiplied by its variation with the base.

### Evaluation of the Amari–Chentsov tensor

From the form (122) of the Fisher metric in coordinates $\left( v , u \right)$, the Amari–Chentsov tensor on all contravariant indices can be computed:

203 $$\begin{aligned} \frac{T}{N}&\equiv \frac{ {\partial }^3 }{ \partial {\theta }^3 } \! \left( \frac{{\psi }}{N} \right) \nonumber \\&= - 2 \frac{ \left( dv / d\theta \right) \left( du / d\eta \right) }{ { \left[ 1 + u v \right] }^3 } \left[ {\varvec{\nu }} \left( 1 - u v \right) + \sqrt{ \frac{1}{4} - {\varvec{\nu }}^2 } \left( u + v \right) \right] \end{aligned}$$

*T* is symmetric under $u \leftrightarrow v$, but its magnitude is not conserved along the stationary-path trajectories. The measure changes $\left( dv / d\theta \right)$ and $\left( du / d\eta \right)$ are the same as those in the Fisher metric, but in addition to these the term $u + v$ is not invariant.

### Connection coefficients in the coherent-state connection

Among the nonzero connection coefficients for the dual coherent-state connections (90), the only independent components are for the rotated variables $\theta$ of Eq. (95) and $\eta$ of Eq. (115). They are

204 $$\begin{aligned} {{\Gamma }^{\left( \theta \right) }_{\theta \theta }}^{\theta }&= \frac{\partial }{\partial \theta } \log \left( \frac{\partial \underline{\nu }}{ \partial \theta } \right)&{{\Gamma }^{\left( \eta \right) *}_{\eta \eta }}^{\eta }&= \frac{\partial }{\partial \eta } \log \left( \frac{\partial \bar{\nu }}{ \partial \eta } \right) \end{aligned}$$

The covariant components of the time derivative of vector fields $\delta \theta$ and $\delta \eta$ defined in Eq. (84), for transport respectively along $\dot{\theta }$ and $\dot{\eta }$, evaluate to

205 $$\begin{aligned} \left( \frac{\partial }{\partial \tau } \delta \theta \right) + \dot{\theta } \left( {\nabla }_{\theta } \delta \theta \right)&= \left( \frac{d}{d\tau } \delta \theta \right) + \delta \theta \, \dot{\theta } \frac{\partial }{\partial \theta } \log \left( \frac{\partial \underline{\nu }}{ \partial \theta } \right) \nonumber \\&= \delta \theta \left[ \frac{ {\partial }^2 \mathcal {\hat{L}} }{ \partial \theta \partial \nu } + \frac{d}{d\tau } \log \left( \frac{1}{4} - {\underline{\nu }}^2 \right) \right] \nonumber \\&= \delta \theta \nonumber \\ \left( \frac{\partial }{\partial t} \delta \eta \right) + \dot{\eta } \left( {\nabla }^{*}_{\eta } \delta \eta \right)&= \left( \frac{d}{d\tau } \delta \eta \right) + \delta \eta \, \dot{\eta } \frac{\partial }{\partial \eta } \log \left( \frac{\partial \bar{\nu }}{ \partial \eta } \right) \nonumber \\&= - \delta \eta \left[ \frac{ {\partial }^2 \mathcal {\tilde{\hat{L}}} }{ \partial \nu \partial \eta } - \frac{d}{d\tau } \log \left( \frac{1}{4} - {\bar{\nu }}^2 \right) \right] \nonumber \\&= - \delta \eta \end{aligned}$$

The corrections from the coherent-state connection coefficients (204) may be understood immediately by using the first line of Eq. (114) and Eq. (115) together with the time dependencies (107), to write

206 $$\begin{aligned} \delta {\theta }_{\tau }&= \delta {\theta }_T \frac{ \left( \frac{1}{4} - {\underline{\nu }}_T^2 \right) }{ \left( \frac{1}{4} - {\underline{\nu }}_{\tau }^2 \right) } e^{\tau - T}&\delta {\eta }_{\tau }&= \delta {\eta }_0 \frac{ \left( \frac{1}{4} - {\bar{\nu }}_0^2 \right) }{ \left( \frac{1}{4} - {\bar{\nu }}_{\tau }^2 \right) } e^{-\tau } \end{aligned}$$

The geometric invariants (205) capture the exponential growth or decay from Eq. (206), while connection terms remove measure factors ,  for exponential relative to coherent-state coordinates.

The Fisher metric *g*, unlike $\delta \theta$ and $\delta \eta$, has no intrinsic time dependence and changes only due to change in the net binomial parameter of the importance distribution. Its covariant derivatives (85), with connection coefficients (204), then produce the two independent components of variation from $\dot{\theta }$ and $\dot{\eta }$ of

207 $$\begin{aligned} \dot{\theta } {\nabla }_{\theta } g&= \dot{\theta } \left[ \frac{\partial g}{\partial \theta } - g \frac{\partial }{\partial \theta } \log \left( \frac{\partial \underline{\nu }}{ \partial \theta } \right) \right] \nonumber \\&= \dot{\theta } \frac{\partial g}{\partial \theta } - g \frac{d}{d\tau } \log \left( \frac{1}{4} - {\underline{\nu }}_{\tau }^2 \right) \nonumber \\&= \left( \dot{v} \frac{\partial }{\partial v} \log {\hat{\Phi }}_0^2 \right) g \nonumber \\ -\dot{\eta } {\nabla }^{*}_{\eta } g&= - \dot{\eta } \left[ \frac{\partial g}{\partial \eta } - g \frac{\partial }{\partial \eta } \log \left( \frac{\partial \bar{\nu }}{ \partial \eta } \right) \right] \nonumber \\&= -\dot{\eta } \frac{\partial g}{\partial \eta } + g \frac{d}{d\tau } \log \left( \frac{1}{4} - {\bar{\nu }}_{\tau }^2 \right) \nonumber \\&= - \left( \dot{u} \frac{\partial }{\partial u} \log {\hat{\Phi }}_0^2 \right) g \end{aligned}$$

These are reproduced as Eq. (126) in the text.

### Two-dimensional divergences of the large-deviation function and their integrals

In the exponential family of tilted distributions with tilting parameter $\theta$, over a base distribution with exponential parameter $\eta$, the large-deviation function of two arguments is constructed as

208 $$\begin{aligned} {\psi }^{*} \! \left( n ; \eta \right) = \theta \! \left( n ; \eta \right) n - \psi \! \left( \theta \! \left( n ; \eta \right) ; \eta \right) \end{aligned}$$

Its variation with $\eta$ at fixed *n* is given by

209 $$\begin{aligned} { \left. \frac{\partial {\psi }^{*}}{\partial \eta } \right| }_{n} = { \left. \frac{\partial \theta }{\partial \eta } \right| }_{n} \left( n - { \left. \frac{\partial \psi }{\partial \theta } \right| }_{\eta } \right) - { \left. \frac{\partial \psi }{\partial \eta } \right| }_{\theta } \end{aligned}$$

By definition of $\theta \! \left( n ; \eta \right)$ as the inverse function of $n \! \left( \theta ; \eta \right) \equiv \partial \psi \! \left( \theta ; \eta \right) / \partial \theta$, the term $\left( n - \partial \psi / \partial \theta \right) \equiv 0$ at all *n*. Therefore the second derivative

210 $$\begin{aligned} \frac{ {\partial }^2 {\psi }^{*} }{ \partial n \, \partial \eta }&= - { \left. \frac{\partial \theta }{\partial n} \right| }_{\eta } \frac{ {\partial }^2 \psi }{ \partial \theta \, \partial \eta } \nonumber \\&= - \frac{ {\partial }^2 {\psi }^{*} }{ \partial n^2 } \frac{ {\partial }^2 \psi }{ \partial \theta \, \partial \eta } \nonumber \\&= - { \left( \frac{ {\partial }^2 \psi }{ \partial {\theta }^2 } \right) }^{-1} \frac{ {\partial }^2 \psi }{ \partial \theta \, \partial \eta } \nonumber \\&= -1 \end{aligned}$$

The third line of Eq. (210) uses additivity of $\theta$ and $\eta$ to cancel the two factors, which equal respectively $g^{-1}$ and *g*.

The log-ratio of the conditional large-deviation probabilities in Eq. (130) may be written as the integral

211 $$\begin{aligned} \log \left( \frac{ P \! \left( n_B \mid n_A ; {\eta }_2 \right) }{ P \! \left( n_B \mid n_A ; {\eta }_1 \right) } \right)&= \int _{{\eta }_1}^{{\eta }_2} d\eta \frac{\partial }{\partial \eta } \log P \! \left( n_B \mid n_A ; \eta \right) \nonumber \\&= - \int _{{\eta }_1}^{{\eta }_2} d\eta \frac{\partial }{\partial \eta } \left[ {\psi }^{*} \! \left( n_B ; \eta \right) - {\psi }^{*} \! \left( n_A ; \eta \right) \right] \nonumber \\&= - \int _{{\eta }_1}^{{\eta }_2} \! \! \int _{n_A}^{n_B} d\eta \, dn \frac{ {\partial }^2 {\psi }^{*} }{ \partial n \, \partial \eta } \nonumber \\&= \int _{{\eta }_1}^{{\eta }_2} \! \! \int _{n_A}^{n_B} d\eta \, dn { \left. \frac{\partial \theta }{\partial n} \right| }_{\eta } \frac{ {\partial }^2 \psi }{ \partial \theta \, \partial \eta } \nonumber \\&= \int _{{\eta }_1}^{{\eta }_2} \! \! \int _{{\theta }_A}^{{\theta }_B} d\eta \, d\theta \frac{ {\partial }^2 \psi }{ \partial \theta \, \partial \eta } \nonumber \\&= \int _{{\eta }_1}^{{\eta }_2} \! \! \int _{n_A}^{n_B} d\eta \, d_{\theta } n \nonumber \\&= \left( n_B - n_A \right) \left( {\eta }_2 - {\eta }_1 \right) \end{aligned}$$

giving Eq. (131) in the text. The conversion from the fifth to the sixth line in Eq. (211) makes use of the two alternative ways of expressing the inner product in contravariant/covariant coordinates given in Eq (76).

## Footnotes

1 It is impossible to fairly represent the motivations and scope of what has now become a significant fraction of work spanning dynamical systems and statistical mechanics. The study of generating functions for reverse-time trajectories began in dynamical systems [16, 25, 31, 32], and was later taken up in similar form for non-equilibrium stochastic processes [14, 19, 20, 24, 26, 38–41, 45, 46]. Reviews of parts of this literature from different stages in its development and from different domain perspectives include [15, 27, 37, 64].

2 Wigner introduced this function to treat quantum density matrices, such as those arising in the Schwinger-Keldysh time-loop 2FFI [43, 63], and it is closely related to the Glauber–Sudarshan “*P*-representation” [17, 34, 72]. Equivalent constructions are widely applied to problems in time-frequency analysis or optics [12, 75], and an example of the map from quantum to classical 2FFI Wigner functions is given in [67].

3 Note that it is the dual connection ${{\Gamma }^{D*}}^{kij} \equiv 0$, written in all-*covariant* indices, which vanishes as the affine connection on the mixture family.

4 In the basis of the original species counts, by Eqs. (45) and (55), the measures between number-potential and coherent-state basis vectors are

90 $$\begin{aligned} \frac{\partial }{\partial {\theta }^i}&= {\phi }^{\dagger i} \frac{\partial }{\partial {\phi }^{\dagger i}}&\frac{\partial }{\partial {\phi }^{\dagger i}}&= e^{- {\theta }^i} \frac{\partial }{\partial {\theta }^i} \nonumber \\ \frac{\partial }{\partial {\eta }^i}&= {\varphi }^i \frac{\partial }{\partial {\varphi }^i}&\frac{\partial }{\partial {\varphi }^i}&= e^{- {\eta }^i} \frac{\partial }{\partial \eta } \end{aligned}$$

Therefore the connection coefficients (90) in the species basis are

88 $$\begin{aligned} {{\Gamma }^{\left( \theta \right) }_{ki}}^j&= {\delta }_{ki} {\delta }_i^j&{{\Gamma }^{\left( \eta \right) *}_{ki}}^j&= {\delta }_{ki} {\delta }_i^j \end{aligned}$$

For various reasons it will, however, not be convenient to work in the species basis, and the resulting connection coefficients will not generally be constant.

5 It implies constancy of a tower of higher-order correlation functions expressing exact conservation of the underlying variable *N*, though we do not develop the 2FFI representation of correlation functions in this paper.

6 In one dimension, the conditional probability is a ratio because the only way to escape beyond $n_B$ is to have also exceeded $n_A < n_B$. In higher dimensions, a similar construction of the conditional can be made, but escapes must be computed along the local least-action trajectories under the action (44), and conditions computed for thresholds that lie in sequence along those trajectories. The leading exponential approximations to such probabilities are the standard first-passage constructions of Freidlin–Wentzell theory [30].
